# A π-extended β-diketiminate ligand *via* a templated Scholl approach†

**DOI:** 10.1039/d4cc01627k

**Published:** 2024-06-06

**Authors:** Lars Killian, Martin Lutz, Arnaud Thevenon

**Affiliations:** a Organic Chemistry and Catalysis, Institute for Sustainable and Circular Chemistry, Faculty of Science, Utrecht University Universiteitsweg 99 3584 CG Utrecht The Netherlands a.a.thevenon-kozub@uu.nl; b Structural Biochemistry, Bijvoet Centre for Biomolecular Research, Faculty of Science, Utrecht University Universiteitsweg 99 3584 CG Utrecht The Netherlands

## Abstract

We report a templated Scholl oxidation strategy for the preparation of the first β-diketiminate (BDI) ligands embedded within a 24-electron π-system backbone. The resulting benzo[*f*,*g*]tetracene BDI ligand was coordinated to a zinc centre and electrochemical studies showed the redox active nature of the ligand.

## Abstract 2

We report a novel redox active β-diketiminate ligand embedded within a 24-electron π-system, prepared using a templated Scholl oxidation strategy.

## Graphical abstract

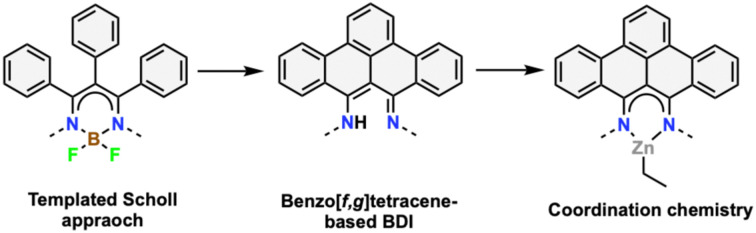

Polycylic (hetero-)aromatic hydrocarbons (PAHs) play a pivotal role in the field of organic optoelectronic materials due to their unique electronic, optical and self-assembly properties.^1–3^ Their interesting properties have also received attention from the field of coordination chemistry, notably with the investigations of “superbenzene”-like ligands,^4^ perylene-based ligands^5^ and extended π-conjugated porphyrins.^6^

From the perspective of ligand design in homogeneous catalysis, it is expected that a ligand embedded in a PAH could act as an electron reservoir to store and provide multiple electrons, on demand, to the active metal centre.^7^ However, the lack of suitable and easily accessible ligands hinder further development in the field. One of the reasons is the challenging “bottom-up” synthesis of the extended conjugated π-system, often involving cyclo-dehydrogenation methods such as the Scholl reaction.^8^ Another reason is the large number of steps often required to install solubilizing groups to prevent strong π–π stacking. In many cases, the final product is obtained only on a milligram scale. Even then, the properties of the resulting material often hinder solution state characterization and reactivity studies.

β-Diketiminates (BDIs) are a well-known class of ligands widely used in homogeneous catalysis and coordination chemistry.^9^ Despite being typically seen as spectator ligands, their non-innocent behaviour has been widely investigated, with examples such as chemical reactivity at the γ-position and redox non-innocence.^10^ Using a phenyl-substituted BDI, it was shown that the ligand system is capable of acting as an electron reservoir through conjugation to aromatic substituents (Fig. 1a).^10*d*^ Despite this, the incorporation of large, conjugated π-systems within BDI-type ligands has been largely limited to 2,2′-dipyrromethene-type ligands (Fig. 1b). Interestingly, few studies used the smallest polycyclic odd alternant hydrocarbon found in graphene sheets, phenalenyl,^11^ incorporated into BDI ligands.^12^ Complexes of these ligands were used as molecular conductor,^13^ and for various catalytic transformations.^12*a*,14^ The ligand's stability across three different oxidation states was further shown to modulate the activity of aluminium complexes in the hydroamination reaction.^15^

**Fig. 1:**
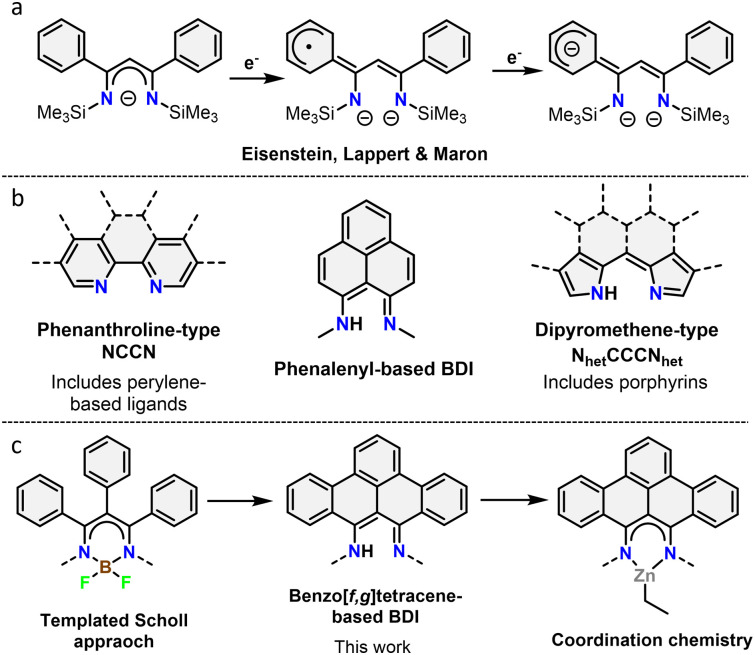
(a) Redox non-innocence on a phenyl substituted BDI ligand. (b) Overview of different binding pockets reported in nanographene ligands. (c) Overview of the work described herein.

In this context, we report the synthesis and characterization of the first example of BDI ligands embedded within a benzo[*f,g*]tetracene backbone. The ligand is isolated in relatively few steps and successfully coordinated to Zn demonstrating its potential as ligand for transition metals (Fig. 1c). Cyclic voltammetry was used to reveal the redox active nature of the ligand.

The 4-step synthesis of the β-,γ-*t*Bu-phenyl substituted BDI pre-ligand (^TBP^BDI, Scheme 1) starts with the synthesis of the corresponding 1,3-diketone ^TBP^AcAc. ^TBP^AcAc was prepared in 75% yield *via* soft-enolization of 4-*t*Bu-desoxybenzoin (^*t*Bu^DOB)^16^ with Hünig's base/MgBr_2_·Et_2_O and reaction with 4-*t*Bu-benzoyl chloride.^17^ Inspired by a reported procedure for the synthesis of bulky BDI ligands,^18^ double imine condensation between ^TBP^AcAc and 4-mesityl aniline was achieved using TiCl_4_ as a dehydration agent, giving pre-ligand ^TBP^BDI in 87% yield. The number of signals in both ^1^H and ^13^C NMR spectra of ^TBP^BDI suggests a *cis–trans* conformation of the diimine motif in the solution state, as opposed to a *C*_2v_ symmetric *cis–cis* or *trans–trans* conformation (Scheme 1).

**Scheme 1:**
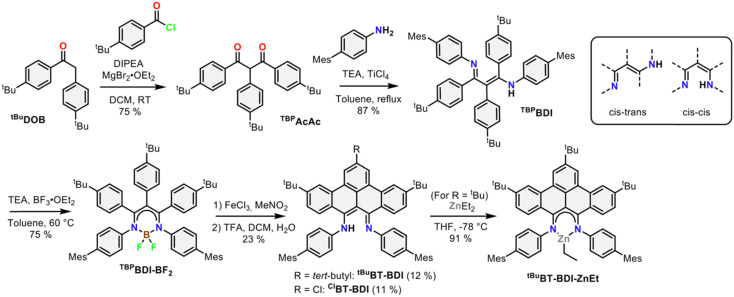
Synthetic route towards BT-BDI and its ZnEt complex.

With pre-ligand ^TBP^BDI in hand, several conditions were screened for the cyclodehydrogenation reaction to form a large, extended π-system (see ESI†). Interestingly, when using DDQ and methanesulfonic acid (MSA), full conversion was observed within an hour at room temperature by TLC. Dark crystals suitable for single-crystal XRD were grown from a toluene solution of the crude product at 5 °C and show the formation of a C–N bond as opposed to a C–C bond, leading to an indole-type framework (^TBP^Indole·HMeSO_3_, Scheme 2 and Fig. S70, ESI†). Similar oxidative C–N bond formation reactions are known in literature, for example using hypervalent iodide reagents as oxidants.^19^ We reason that the formation of a C–N bond over the desired C–C bonds is due to the solution-state *cis–trans* conformation of the diimine motive discussed above. This prompted us to investigate a novel templating approach to aid the favourable positioning of the aromatic rings for C–C bond formation.

**Scheme 2:**
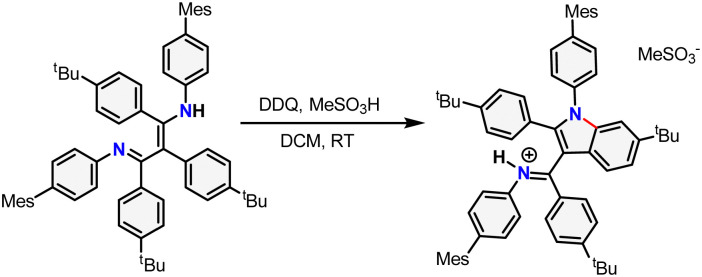
Synthesis of ^TBP^Indole·HMeSO_3_ from ^TBP^BDI by Scholl oxidation using DDQ and MSA. The newly formed bond is drawn in red.

Inspired by the synthesis of BODIPY-type compounds, as well as several examples of successful π-extension of such compounds using Scholl oxidation,^20^ we set out to explore the use of a BF_2_ template for the synthesis of a π-extended BDI from ^TBP^BDI. Reaction of pre-ligand ^TBP^BDI with BF_3_·OEt_2_ in the presence of NEt_3_^20*b*^ gave a boron complex in a 75% yield, showing *C*_2v_ symmetry in NMR spectroscopy as well as the incorporation of BF_2_ by ^19^F and ^11^B NMR spectroscopy. The structure of ^TBP^BDI-BF_2_ was further confirmed by single crystal X-ray structure determination (Fig. S71, ESI†).

With the boron complex in hand, Scholl oxidation conditions were investigated. Reaction with FeCl_3_/MeNO_2_ showed the best results with good conversion towards four main products (Fig. 2a). Two of them were isolated by two consecutive column chromatographic separations. Their ^1^H NMR spectra reveal highly similar patterns with multiple signals overlapping, pointing towards the formation of two closely related, yet distinct, *C*_2v_ symmetric compounds. The HRMS of the first compound showed a difference of 4 *m*/*z* compared to pre-ligand ^TBP^BDI, suggesting two oxidative dehydrogenations. This, combined with a more detailed analysis of the ^1^H NMR spectrum (Fig. 2d) confirmed the formation of a benzo[*f,g*]tetracene backbone on the BDI (^*t*Bu^BT-BDI). In addition, concomitant loss of BF_2_ is observed, as evidenced by the ^1^H-signal at 13.2 ppm corresponding to the amine proton (Fig. S28, ESI†). The aliphatic region of the ^1^H NMR spectrum of the other compound shows the absence of 9H, *i.e.* one of the *t*Bu-signals of the benzo[*f,g*]tetracene backbone (Fig. S33, ESI†). This, combined with HRMS data suggests the substitution of *t*Bu for a chloride (^Cl^BT-BDI). Although chlorination is commonly observed as a side-reaction in FeCl_3_ mediated Scholl reactions,^21^ this is, to the best of our knowledge, the first example of regioselective chloride-for-*t*Bu substitution.

**Fig. 2:**
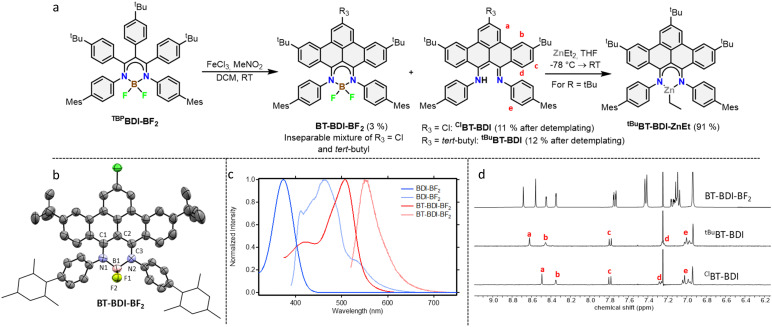
(a) Reaction scheme showing the reaction of ^TBP^BDI-BF_2_ to form BT-BDI-BF_2_, ^Cl^BT-BDI and ^*t*Bu^BT-BDI and further reaction of ^*t*Bu^BT-BDI to form ^*t*Bu^BT-BDI-ZnEt. (b) Displacement ellipsoid plot (50% probability) of the asymmetric unit of BT-BDI-BF_2_ in the crystal. Hydrogen atoms are omitted, and mesityl groups are shown as wireframe for clarity. Only the major disorder component is shown. Severely disordered methyl *t*-butyl ether molecules are omitted. There is substitutional disorder for the substituents at C47 between chlorine and *t*-butyl in a ratio of 57.9(4) : 42.1(4). (c) Comparison of the absorption (dark traces) and emission (light traces) spectra of ^TBP^BDI-BF_2_ and BT-BDI-BF_2_ measured in DCM (left). For the emission spectra, the sample was excited at the wavelength of the shown absorption maximum. (d) Comparison of the aromatic region of the ^1^H NMR spectrum of BT-BDI-BF_2_, ^*t*Bu^BT-BDI and ^Cl^BT-BDI with relevant assignments corresponding to (a) of this same figure measured in CDCl_3_ (right).

The two other products, isolated as one fraction in a 3% total yield, co-crystalized *via* slow evaporation of a solution in MTBE or Et_2_O to form orange blocks and were analysed by single-crystal X-ray structure determination. Interestingly, the crystal structure consists of the two previously described compounds – with chloride substitution and without – but coordinated to BF_2_ (Fig. 2b). This suggests partial deprotection of the BT-BDIs takes place during the reaction. The benzo[*f,g*]tetracene backbone is found to be curved (Fig. S74, ESI†) and the *p*-mesityl aniline arms bend out of the BDI plane (Fig. S75, ESI†), likely due to steric repulsion with the benzo[*f*,*g*]tetracene unit. The boron centre is nearly tetrahedral with angles around boron all close to 109° (Table S3, ESI†). The delocalization over the N–C–C–C–N binding pocket is clearly seen in the solid-state structure, with both C–N bonds and C–C bonds being of similar length (Table S3, ESI†). The solution state data is in agreement with the solid-state structure, with the ^1^H NMR spectrum indicating *C*_2v_ symmetric compounds. Efficient deprotection of BT-BDI-BF_2_ was achieved by treating the crude reaction mixture with 90/5/5 TFA/DCM/H_2_O,^22^ giving a total yield of 12% for ^*t*Bu^BT-BDI and 11% for ^Cl^BT-BDI.

The extension of the conjugated system leads to a bathochromic shift in the absorption and emission spectra (Fig. 2c). Comparing ^TBP^BDI-BF_2_ with BT-BDI-BF_2_, the highest wavelength absorption maximum – corresponding to the π → π* transition – shifts from 374 nm to 507 nm, a shift of 133 nm. The corresponding emission peaks shifts by 85 nm upon extension of the conjugated system. The smaller Stokes shift observed for BT-BDI-BF_2_ compared to ^TBP^BDI-BF_2_ is in agreement with the more rigid structure of the former. It is worth noting, however, that the BT-BDI-BF_2_ is a mixture of the chloride substituted and *t*Bu-substituted compounds.

To further probe the coordination chemistry behaviour of the BT-BDI framework, we reacted the new ligand ^*t*Bu^BT-BDI with 1.2 equivalents of diethylzinc in THF at −78 °C. Upon reaction, the colour of the solution gradually changed from dark red to dark blue/purple. After evaporation of the volatiles, ^*t*Bu^BT-BDI-ZnEt was obtained as a dark purple powder in 91% yield. The ^1^H NMR spectrum shows the absence of the –NH proton, as well as the presence of an aliphatic triplet (1.50 ppm) which is part of the same spin system of an aliphatic quartet (0.92 ppm), together corresponding to the ethyl ligand (Fig. S45, ESI†). Although a small amount of THF is still present in the sample according to ^1^H NMR, this is far below 1 equivalent, which suggests the absence of an additional THF ligand and a trigonal geometry around the zinc centre. Further spectroscopic evidence for the structure of ^*t*Bu^BT-BDI-ZnEt is given in the ESI† (Fig. S51 and S52, ESI†). The UV-vis spectrum of ^*t*Bu^BT-BDI-ZnEt shows a large bathochromic shift of 103 nm compared to the free ligand, shifting from 475 to 578 nm (*ε* = 9.9 × 10^3^ M^−1^ cm^−1^, Fig. S59, ESI†).

Cyclic voltammetry (CV) was used to study the redox activity of the new ligands and complexes. For BT-BDI-BF_2_, the first event when scanning cathodically (*E*_1/2_ = −1.61 V *vs.* Fc/Fc^+^) is fully reversible (Fig. 3, left and Fig. S60, ESI†). The following reversible reduction at approximately −2.2 V *vs.* Fc/Fc^+^, as well as the reversible oxidation event on the return scan at approximately 0.8 V *vs.* Fc/Fc^+^, each seem to be consisting of two overlapping events. This is confirmed by the DPV (Fig. 3, right), which shows two peaks in the regions of both these events. We attribute these overlapping events to the presence of both the chloride- and *t*Bu-substituted compounds. The observed redox non-innocence shows the relatively facile introduction of two electrons into the conjugated ligand, which is largely in line with what has been observed for phenalenyl-based BDIs.^15^ The electrochemical HOMO–LUMO gap, taken as the potential difference between the peak potentials in the anodic DPV at −1.58 V *vs.* Fc/Fc^+^ and the average peak potential of the two oxidative peaks at 0.77 V *vs.* Fc/Fc^+^ is 2.35 eV. This corresponds well with the optical HOMO–LUMO gap, taken from the onset of the absorbance maximum at 475 nm (553 nm), of 2.24 eV. The cyclic voltammetry of the free ligands (Fig. S62–S67, ESI†) is markedly different than that of BT-BDI-BF_2_. Interestingly, none of the observed electrochemical events are reversible. Furthermore, two extra reduction peaks are observed in the second scan, and additional measurements revealed that these events are both coupled to a single oxidation event at 0.35 V *vs.* Fc/Fc^+^ (Fig. S67, ESI†). The irreversibility of the oxidation events, combined with the fact that the oxidations are coupled to two reductive events implies the occurrence of (a) chemical reaction(s) at oxidative potentials for BT-BDI. We hypothesize that through further oxidative dehydrogenation steps, it is possible to form a larger extended π-system, with C–C bond formation between the benzo[*f,g*]tetracene unit and the rings originating from the 4-mesityl aniline. We are currently investigating this possibility.

**Fig. 3:**
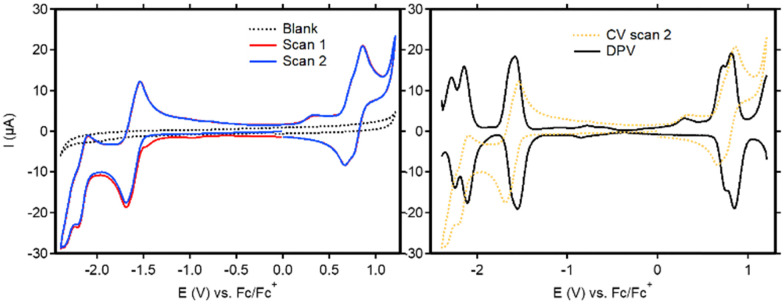
CV (left) and DPV (right) traces of BT-BDI-BF_2_. All measurements were performed on 1 mM solutions of analyte in DCM with 0.1 M *n*Bu_4_NPF_6_ using a glassy carbon WE, a Pt wire CE and a Ag/AgNO_3_ (10 mM) RE.

In conclusion, we have reported the facile synthesis of a BDI ligand bearing a π-extended benzo[*f,g*]tetracene (BT) backbone. Using a novel boron-templated strategy, this ligand is obtained together with a second BT-BDI compound, in which one *t*Bu-group has been selectively substituted for a chloride, which is uncommonly observed in Scholl oxidations. This strategy is, to the best of our knowledge, the first reported case in which coordination templating steers reactivity in Scholl oxidation and paves the way for the synthesis of other nanographene ligands. Electrochemical characterization shows the aptitude of the ligand framework to undergo multiple reduction steps, confirming the high redox activity of the ligand. Coordination to a Zn centre further shows that this new ligand framework can be used to explore the coordination chemistry of BDI ligands bearing large extended π-systems.

A. T. thanks the Dutch Research Council (NWO) for funding *via* Veni grant (Veni.212.039) and Utrecht University for financial support. The X-ray diffractometer has been financed by NWO.

## Conflicts of interest

There are no conflicts to declare.

## Supplementary Material

CC-060-D4CC01627K-s001

CC-060-D4CC01627K-s002

## References

[cit1] Wu J., Pisula W., Müllen K. (2007). Chem. Rev..

[cit2] Narita A., Wang X.-Y., Feng X., Müllen K. (2015). Chem. Soc. Rev..

[cit3] Borissov A., Maurya Y. K., Moshniaha L., Wong W.-S., Żyła-Karwowska M., Stępień M. (2022). Chem. Rev..

[cit4] Draper S. M., Gregg D. J., Schofield E. R., Browne W. R., Duati M., Vos J. G., Passaniti P. (2004). J. Am. Chem. Soc..

[cit5] Chouai A., Wicke S. E., Turro C., Bacsa J., Dunbar K. R., Wang D., Thummel R. P. (2005). Inorg. Chem..

[cit6] Lewtak J. P., Gryko D. T. (2012). Chem. Commun..

[cit7] Nie W., Tarnopol D. E., McCrory C. C. L. (2021). Curr. Opin. Electrochem..

[cit8] Jassas R. S., Mughal E. U., Sadiq A., Alsantali R. I., Al-Rooqi M. M., Naeem N., Moussa Z., Ahmed S. A. (2021). RSC Adv..

[cit9] Bourget-Merle L., Lappert M. F., Severn J. R. (2002). Chem. Rev..

[cit10] Camp C., Arnold J. (2016). Dalton Trans..

[cit11] Ahmed J., Mandal S. K. (2022). Chem. Rev..

[cit12] Mukherjee A., Sen T. K., Mandal S. K., Kratzert D., Stalke D., Döring A., Schulzke C. (2011). J. Chem. Sci..

[cit13] Mandal S. K., Itkis M. E., Chi X., Samanta S., Lidsky D., Reed R. W., Oakley R. T., Tham F. S., Haddon R. C. (2005). J. Am. Chem. Soc..

[cit14] Mukherjee A., Sen T. K., Kr. Ghorai P., Samuel P. P., Schulzke C., Mandal S. K. (2012). Chem. – Eur. J..

[cit15] Mukherjee A., Sen T. K., Ghorai P. K., Mandal S. K. (2013). Sci. Rep..

[cit16] Ren X., Du H. (2016). J. Am. Chem. Soc..

[cit17] Aderibigbe S. O., Coltart D. M. (2019). J. Org. Chem..

[cit18] Hill L. M. R., Gherman B. F., Aboelella N. W., Cramer C. J., Tolman W. B. (2006). Dalton Trans..

[cit19] Ban X., Pan Y., Lin Y., Wang S., Du Y., Zhao K. (2012). Org. Biomol. Chem..

[cit20] Hayashi Y., Obata N., Tamaru M., Yamaguchi S., Matsuo Y., Saeki A., Seki S., Kureishi Y., Saito S., Yamaguchi S., Shinokubo H. (2012). Org. Lett..

[cit21] Lewtak J. P., Gryko D., Bao D., Sebai E., Vakuliuk O., Ścigaj M., Gryko D. T. (2011). Org. Biomol. Chem..

[cit22] Yu M., Wong J. K.-H., Tang C., Turner P., Todd M. H., Rutledge P. J. (2015). Beilstein J. Org. Chem..

