# Supplementary material for: A π-extended β-diketiminate ligand via a templated Scholl approach
Source: Chem Commun (Camb). 2024 Jun 6;60(52):6663–6. doi: 10.1039/d4cc01627k (PMC11198738; doi:10.1039/d4cc01627k)
Supplement: CC-060-D4CC01627K-s001 [file CC-060-D4CC01627K-s001.pdf]

Electronic Supplementary Material (ESI) for ChemComm.
This journal is © The Royal Society of Chemistry 2024

Supplementary Information

A π-extended β-diketiminate ligand *via* a templated Scholl approach

Lars Killian[a], Martin Lutz[b], Arnaud Thevenon*[a]

[a] Organic Chemistry and Catalysis, Institute for Sustainable and Circular Chemistry, Faculty of Science, Utrecht University, Universiteitsweg 99, 3584 CG, Utrecht (The Netherlands)
[b] Structural Biochemistry, Bijvoet Centre for Biomolecular Research, Faculty of Science, Utrecht University, Universiteitsweg 99, 3584 CG, Utrecht (The Netherlands)

S1. General remarks ... 2
S2. Synthesis and characterization ... 3
1,2-bis(4-(tert-butyl)phenyl)ethan-1-one (^{tBu}DOB) ... 3
4-mesityl aniline ... 4
1,2,3-tris(4-(tert-butyl)phenyl)propane-1,3-dione (^{TBP}AcAc) ... 5
^{TBP}BDI ... 6
^{TBP}BDI-BF_2 ... 8
^{tBu}BT-BDI and ^{Cl}BT-BDI ... 9
BT-BDI-BF_2 ... 11
^{tBu}BT-BDI-ZnEt ... 13
^{TBP}Indole·$HMeSO_3$ ... 14
Attempted Scholl oxidation of ^{TBP}BDI using $FeCl_3$ ... 15
S3. NMR spectra ... 16
1,2-bis(4-(tert-butyl)phenyl)ethan-1-one (^{tBu}DOB) ... 16
4-mesityl aniline ... 18
1,2,3-tris(4-(tert-butyl)phenyl)propane-1,3-dione (^{TBP}AcAc) ... 21
^{TBP}BDI ... 23
^{TBP}BDI-BF_2 ... 26
^{tBu}BT-BDI ... 29
^{Cl}BT-BDI ... 32
BT-BDI-BF_2 ... 34
^{tBu}BT-BDI-ZnEt ... 38
^{TBP}Indole·$HMeSO_3$ ... 42
Attempted Scholl oxidation of ^{TBP}BDI using $FeCl_3$ ... 43
S4. UV-VIS and fluorescence ... 45
S5. Electrochemistry ... 47
S6. X-ray crystal structure determinations ... 52
^{TBP}Indole·$HMeSO_3$ ... 52
^{TBP}BDI-BF_2 ... 54
BT-BDI-BF_2 ... 56
S7. IR spectra ... 63
S8. HRMS spectra ... 67
References ... 70

S1. General remarks

Where necessary, manipulations were performed under inert N_2 atmosphere using standard Schlenk technique or in a N_2-filled M. Braun Glovebox. Glassware was dried at 130 °C over-night or flame-dried under dynamic vacuum. Unless otherwise stated, commercial reagents and solvents were used as received. Dichloromethane and toluene were collected from an M. Braun MB-SPS 800 solvent purification system and stored on 3 and 4 Å molecular sieves, respectively. Nitromethane was stored over 3 Å molecular sieves. Triethylamine and diisopropylethylamine were stored over 4 Å molecular sieves. THF was distilled over sodium/benzophenone and stored over 4 Å molecular sieves. HMDSO and C_6D_6 were degassed by sparging with N_2 for 30 minutes or three freeze-pump-thaw cycles and stored on 4 Å molecular sieves.

NMR measurements were performed at 25 °C on a Varian VNMRS400, a Varian MRF400 or a Jeol JNM-ECZL G 400 MHz spectrometer. Chemical shifts in ^1H and ^{13}C are reported relative to TMS with the residual solvent signal[1] as internal standard where the influence of analytes on the solvent can be assumed negligible. All NMR experiments involving air-sensitive compounds were conducted in J. Young NMR tubes under an N_2 atmosphere. Peak multiplicity is quoted as s (singlet), bs (broad singlet), d (doublet), t (triplet) and so on.

FT-IR data was recorded on a PerkinElmer SpectrumTwo Infrared Spectrophotometer with an ATR probe. For air/moisture sensitive compounds, the IR crystal was held under a flow of N_2 and the compound was applied to the crystal as a solution in a volatile solvent.

ESI-MS data was recorded on an Advion Expression L cms equipped with a TLC plate reader (Plate Express) using either MeCN or THF as carrier solvents. HRMS was measured on an Agilent Technologies 6560 ion mobility QTOF using direct infusion.

UV-VIS and Fluorescence date was recorded on a PerkinElmer Lambda950 and JASCO FP-8300, respectively. For the determination of the optical bandgap, the wavelength of the onset was determined by extrapolating the slope at the inflection point to the y-axis and then converted to eV.

Electrochemical measurements were performed in a N_2-filled MBraun labmaster dp glovebox, using an IVIUM potentiostat/galvanostat. A three-electrode set-up was used with Ag/$AgNO_3$ (10 mM) reference electrode, Pt wire counter electrode and glassy carbon (3 mm Ø) working electrode directly in solution. All electrochemical data is referenced to the ferrocene/ferrocenium couple, measured on the same day as the experiment.

Elemental analysis was performed by MEDAC ltd. in the United Kingdom.

S2. Synthesis and characterization

1,2-bis(4-(tert-butyl)phenyl)ethan-1-one (^{tBu}DOB)

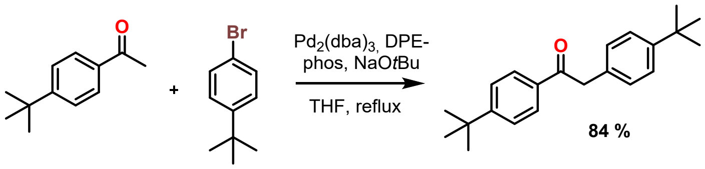

The compound was synthesized according to a modified literature procedure.[2] To a Schlenk flask charged with a magnetic stir bar, $Pd_2(dba)_3$ (888 mg, 0.97 mmol, 1.5 %), DPE-phos (1.26 g, 2.34 mmol, 3.6 %) and NaO^tBu (8.11 g, 84.4 mmol, 1.3 equiv.) was added THF (75 mL) forming a dark brown solution. Then, 1-bromo-4-tert-butylbenzene (11.3 mL, 64.9 mmol, 1 equiv.) was added, followed by 4-tert-butyl acetophenone (13.1 mL, 71.4 mmol, 1.1 equiv.). The mixture was heated to reflux for 3h with the formation of a white precipitate. After cooling to room temperature, water (200 mL) and Et_2O (200 mL) were added, and the organic phase was separated from the aqueous phase. The organics were extracted from the aqueous phase with Et_2O (3 x 100 mL). The resulting red solution was dried on $MgSO_4$, and volatiles were removed under reduced pressure. The residue was purified by column chromatography on silica using a gradient eluent (98/2 → 90/10 PE/ethyl acetate). 1,2-bis(4-(*tert*-butyl)phenyl)ethan-1-one (**^{tBu}DOB**) was isolated as an off-white powder (16.8 g, 84 %). NMR data is consistent with literature.[3]

^{1}H NMR (400 MHz, $CDCl_3$, 298 K): δ = 7.97 (d, $^3J_{H,H}$ = 8.4 Hz, 2H), 7.47 (d, $^3J_{H,H}$ = 8.4 Hz, 2H), 7.34 (d, $^3J_{H,H}$ = 8.3 Hz, 2H), 7.21 (d, $^3J_{H,H}$ = 8.2 Hz, 2H), 4.23 (s, 2H), 1.34 (s, 9H), 1.30 (s, 9H) ppm.

$^{13}C\{^1H\}$ NMR (101 MHz, $CDCl_3$, 298 K): δ = 197.6, 157.0, 149.7, 134.3, 131.8, 129.2, 128.8, 125.7, 45.0, 35.3, 34.6, 31.5, 31.2 ppm.

4-mesityl aniline

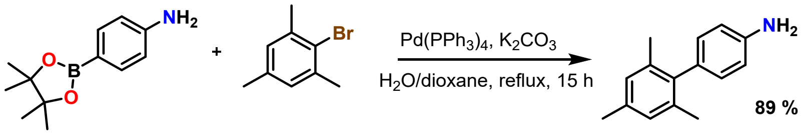

The compound was synthesized according to a literature procedure.[4] To a Schlenk flask equipped with a magnetic stir bar was added aqueous K_2CO_3 (17.7 g, 0.13 mol, 64 mL, 2 M), 1,4-dioxane (72 mL), 4-aminophenylboronic acid pinacol ester (10.2 g, 46.6 mmol, 1.1 equiv.) and 2-bromomesitylene (6.5 mL, 42.4 mmol, 1 equiv.). The resulting mixture was degassed by sparging the solution with N_2, before the addition of $Pd(PPh_3)_4$ (493 mg, 0.42 mmol, 1 mol%). The dark colored mixture was refluxed for 15 hours, after which it was poured into a beaker containing brine (100 mL). The organics were extracted from the aqueous phase with toluene (3 x 50 mL) and the combined organic phases were washed with brine (70 mL), dried with $MgSO_4$ and filtered before removing the solvent under reduced pressure. The residue was purified by column chromatography on neutral alumina (1/1 PE/DCM). **4-Mesityl aniline** was isolated as a yellow solid (7.93 g, 89 %). Over time, slight discoloration of the compound is observed, giving brown solids without notable loss of purity. NMR data is consistent with literature.[4]

^{1}H NMR (400 MHz, $CDCl_3$, 298 K): δ = 7.00 – 6.92 (m, 4H), 6.78 (d, $^3J_{H,H}$ = 8.4 Hz, 2H), 3.70 (bs, 2H), 2.37 (s, 3H), 2.08 (s, 6H) ppm.

^{13}C{^{1}H} NMR (101 MHz, $CDCl_3$, 298 K): δ = 144.8, 139.2, 136.7, 136.3, 131.3, 130.2, 128.1, 115.2, 21.1, 20.9 ppm.

1,2,3-tris(4-(tert-butyl)phenyl)propane-1,3-dione (^{TBP}AcAc)

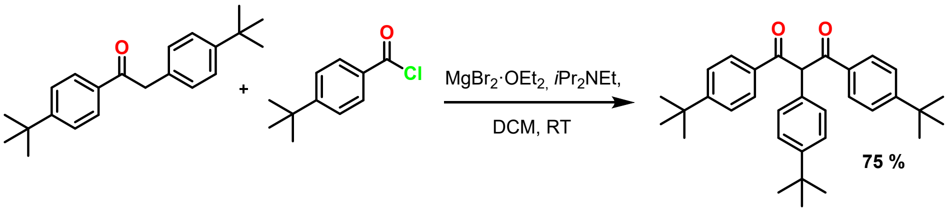

The compound was synthesized according to a modified literature procedure.[5] To a Schlenk flask charged with a magnetic stir bar and 1,2-bis(4-(tert-butyl)phenyl)ethan-1-one (**^{tBu}DOB**) (5.74 g, 18.6 mmol, 1 equiv.) in DCM (60 mL) was added 4-tert-butylbenzoyl chloride (4.0 mL, 20.5 mmol, 1.1 equiv.) followed by $MgBr_2{\cdot}OEt_2$ (12.01 g, 47 mmol, 2.5 equiv.). The yellow slurry was stirred for 15 minutes. Then, iPr_2NEt (9.7 mL, 56 mmol, 3 equiv.) was added over the course of 5 minutes. This led to warming of the solution, but refluxing was avoided. The orange suspension was stirred for 3 hours, before being carefully quenched with aqueous HCl (40 mL, 4 M). To this, DCM (40 mL) and water (40 mL) were added, and the organic phase was separated from the aqueous phase. The organics were further extracted from the aqueous phase with DCM (3 x 40 mL) and the combined organic phase was washed with water (2 x 40 mL) and brine (1 x 40 mL). The organic phase was dried on $MgSO_4$ and concentrated under reduced pressure to yield a yellow, oily solid. The solid was washed with 50 mL hexane, precipitating 1,2,3-tris(4-(tert-butyl)phenyl)propane-1,3-dione (**^{TBP}AcAc**) as a fine, white powder (6.54 g, 75 %).

^{1}H NMR (400 MHz, $CDCl_3$, 298 K): δ = 7.93 (d, $^{3}J_{H,H}$ = 8.8 Hz, 4H), 7.45 (d, $^{3}J_{H,H}$ = 8.7 Hz, 4H), 7.38 (d, $^{3}J_{H,H}$ = 8.7 Hz, 2H), 7.31 (d, $^{3}J_{H,H}$ = 8.6 Hz, 2H), 6.53 (s, 1H), 1.31 (s, 18H), 1.29 (s, 9H) ppm.

^{13}C{^{1}H} NMR (101 MHz, $CDCl_3$, 298 K): δ = 193.8, 157.3, 150.7, 133.5, 130.3, 128.9, 126.0 (overlapping signals), 62.4, 35.3, 34.7, 31.5, 31.2 ppm.

ATR-IR: ν = 3387 (w), 2962 (s), 2906 (m), 2869 (m), 1696 (s), 1663 (s), 1604 (s), 1556 (w), 1511 (w), 1476 (w), 1408 (m), 1364 (m), 1339 (w), 1321 (w), 1293 (m), 1267 (s), 1224 (m), 1211 (m), 1194 (s), 1182 (m), 1108 (m), 1018 (w), 995 (m), 902 (w), 872 (m), 828 (m), 800 (m), 701 (m), 652 (w), 638 (w), 523 (w), 576 (m), 549 (w) cm^{-1}.

HR-MS: m/z = 469.3052 {$[M+H]^+$, calc. 469.3107}.

Elemental analysis: Anal. Calcd. for $C_{33}H_{40}O_2$: C, 84.57; H, 8.60 %. Found: C, 84.44; H, 8.97 %.

^{TBP}BDI

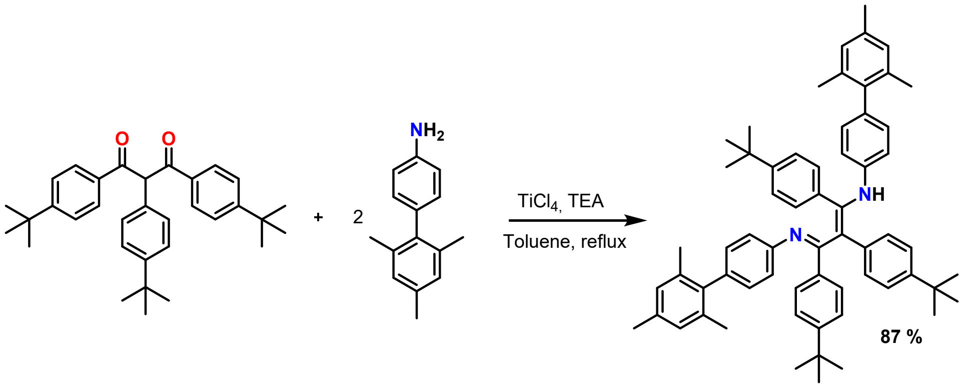

To a Schlenk flask charged with a magnetic stir bar and 4-mesityl aniline (4.06 g, 19.2 mmol, 2 equiv.) was added toluene (100 mL) followed by NEt_3 (10.7 mL, 77 mmol, 8 equiv.). The flask was cooled in a salt-ice bath at -10 °C. A second Schlenk flask was charged with toluene (40 mL) and $TiCl_4$ (2.1 mL, 19.2 mmol, 2 equiv.). The $TiCl_4$ solution was cannulated dropwise to the cooled 4-mesityl aniline solution, upon which the color turned to dark brown, and solids formed. The solution was stirred at room temperature for 1 hour, after which a solution of **^{TBP}AcAc** (4.48 g, 9.6 mmol, 1 equiv.) in toluene (40 mL) was cannulated dropwise to the mixture. The mixture was again stirred at room temperature for 1 hour, and subsequently heated to reflux for 3 more hours. Then, the mixture was filtered over celite and extracted with toluene (200 mL). To the filtrate was added water (100 mL) and the mixture was let to stir for a few minutes, during which the solution went from dark brown to orange. The organics were extracted from the aqueous phase with toluene (3 x 100 mL), and the combined organic phase was washed with water (2 x 100 mL) and brine (1 x 100 mL). The organic phase was dried on $MgSO_4$, and concentrated under reduced pressure to yield an oily orange solid. The residue was purified by column chromatography on silica using a gradient eluent (2/1 → 1/1 PE/DCM → 100 % DCM → 98/2 DCM/MeOH). The compound tends to streak on silica, with an unreliable R_f that is concentration dependent. **^{TBP}BDI** was isolated as an orange solid (7.14 g, 87 %).

^{1}H NMR (400 MHz, $CDCl_3$, 298 K): δ = 7.84 (d, $^3J_{H,H}$ = 8.5 Hz, 2H), 7.37 (d, $^3J_{H,H}$ = 8.5 Hz, 2H), 7.30 (d, $^3J_{H,H}$ = 8.5 Hz, 2H), 7.26 (d, $^3J_{H,H}$ = 8.4 Hz, 2H), 7.14 (d, $^3J_{H,H}$ = 8.4 Hz, 2H), 7.03 (d, $^3J_{H,H}$ = 8.6 Hz, 2H), 6.94 (s, 2H), 6.89 (s, 2H), 6.81 (overlapping doublets, $J_{apparent}$ = 8.3 Hz, 4H), 6.68 (d, $^3J_{H,H}$ = 8.3 Hz, 2H), 6.59 (d, $^3J_{H,H}$ = 8.5 Hz, 2H), 6.12 (s, 1H), 2.33 (s, 3H), 2.30 (s, 3H), 2.05 (s, 6H), 1.96 (s, 6H), 1.30 (s, 9H), 1.28 (s, 9H), 1.21 (s, 9H) ppm.

^{13}C{^{1}H} NMR (101 MHz, $CDCl_3$, 298 K): δ = 168.3, 152.7, 151.9, 149.6, 149.6, 142.9, 142.3, 139.4, 138.9, 138.5, 136.5, 136.5, 136.4, 136.4, 136.4, 135.8, 134.0, 133.1, 130.3, 129.8, 129.3, 129.1, 129.0, 128.8, 128.1, 126.0, 125.1, 125.0, 120.3, 118.7, 118.5, 115.3, 34.8, 34.7, 34.6, 31.4, 31.4, 31.3, 21.1, 21.1, 21.0, 20.9 ppm.

ATR-IR: ν = 3388 (w), 3034 (w), 2959 (s), 2906 (s), 2867 (m), 1587 (m), 1512 (m), 1476 (m), 1405 (w), 1362 (w), 1337 (w), 1267 (m), 1234 (w), 1203 (w), 1187 (w), 1110 (w), 1056 (w), 1015 (w), 1003 (w), 920 (w), 883 (w), 830 (m), 746 (w), 574 (w), 562 (w), 520 (w) cm^{-1}.

HR-MS: m/z = 855.5631 {$[M+H]^+$, calc. 855.5617}.

Elemental analysis: Anal. Calcd. for $C_{63}H_{70}N_2$: C, 88.48; H, 8.25; N, 3.27 %. Found: C, 87.80; H, 8.48; N, 3.23 %.

Note: Most aromatic signals in the ^{1}H and ^{13}C NMR could not be unambiguously assigned using the data collected. The number of peaks, their multiplicity and their integration are consistent with the expected values.

For small scale experiments, it was found that the use of the bench stable $TiCl_4(THF)_2$ was equally effective as $TiCl_4$ and in some cases easier to handle. $TiCl_4(THF)_2$ can be obtained in one step from $TiCl_4$ according to literature protocol.[6]

^{TBP}BDI-BF_2

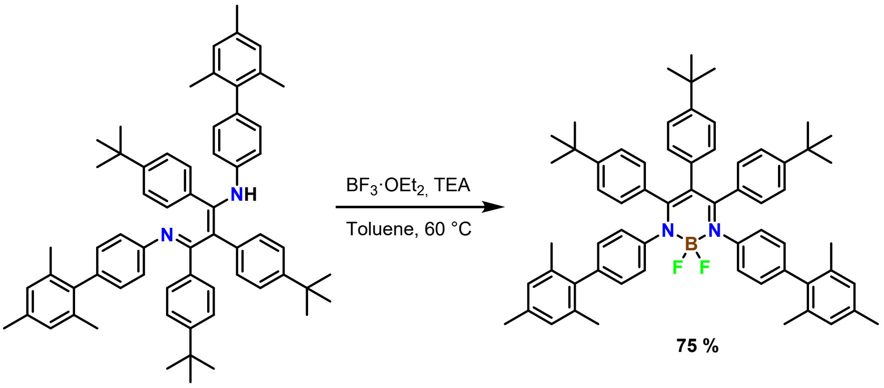

This compound was synthesized according to a modified literature procedure.[7] To a Schlenk flask charged with a magnetic stir bar and **^{TBP}BDI** (1.2 g, 1.4 mmol, 1 equiv.) in toluene (60 mL) was slowly added NEt_3 (12 mL), followed by the dropwise addition of $BF_3·OEt_2$ (12 mL). Upon addition, the color of the solution changed from yellow to dark red, with the formation of precipitate. The resulting suspension was heated to 60 °C for 2 hours, after which all volatiles were removed *in vacuo*. The resulting brown suspension was extracted with toluene, filtered, and concentrated again. The residue was purified by column chromatography on silica (90/10 PE/ethyl acetate). **^{TBP}BDI-BF_2** was isolated as an off-white powder (952 mg, 75 %). Crystals suitable for single-crystal XRD were grown from a solution in DCM/hexane at -25 °C.

^{1}H NMR (400 MHz, $CDCl_3$, 298 K): δ = 7.24 (d, $^3J_{H,H}$ = 8.2 Hz, 4H), 6.91 (d, $^3J_{H,H}$ = 8.7 Hz, 4H), 6.89 – 6.85 (m, 8H), 6.82 (d, $^3J_{H,H}$ = 8.5 Hz, 4H), 6.77 (d, $^3J_{H,H}$ = 8.5 Hz, 2H), 6.62 (d, $^3J_{H,H}$ = 8.5 Hz, 2H), 2.29 (s, 6H), 1.81 (bs, 12H), 1.08 (s, 18H), 1.06 (s, 9H) ppm.

^{13}C{^{1}H} NMR (101 MHz, $CDCl_3$, 298 K): δ = 166.3, 150.6, 148.0, 140.8, 139.0, 138.7, 136.6, 136.1, 134.6, 133.0, 132.9, 129.6, 128.8, 128.8, 128.0, 123.8, 123.6, 34.5, 34.2, 31.2, 31.1, 21.1, 20.6 ppm.

^{19}F NMR (376 MHz, $CDCl_3$, 298 K): δ = -124.7 (q, $^1J_{B,F}$ = 24.9 Hz) ppm.

^{11}B NMR (128 MHz, $CDCl_3$, 298 K): δ = -0.46 (t, $^1J_{B,F}$ = 29.6 Hz) ppm.

ATR-IR: ν = 3030 (w), 2960 (m), 2922 (m), 2866 (m), 1612 (w), 1569 (w), 1540 (m), 1511 (w), 1477 (m), 1459 (s), 1384 (s), 1313 (w), 1266 (w), 1202 (w), 1135 (w), 1106 (w), 1055 (m), 1041 (m), 1014 (m), 1005 (m), 845 (m), 829 (w), 786 (w), 742 (w), 664 (w), 570 (w) cm^{-1}.

ESI-MS: No signal observed.

Elemental analysis: Anal. Calcd. for $C_{63}H_{69}BF_2N_2$: C, 83.79; H, 7.70; N, 3.10 %. Found: 83.20; H, 7.85; N, 2.91 %.

Note: Some signals in the ^{1}H and ^{13}C NMR could not be unambiguously assigned using the data that was collected. The number of peaks, their multiplicity and their integration are consistent with the expected values.

^{tBu}BT-BDI and ^{Cl}BT-BDI

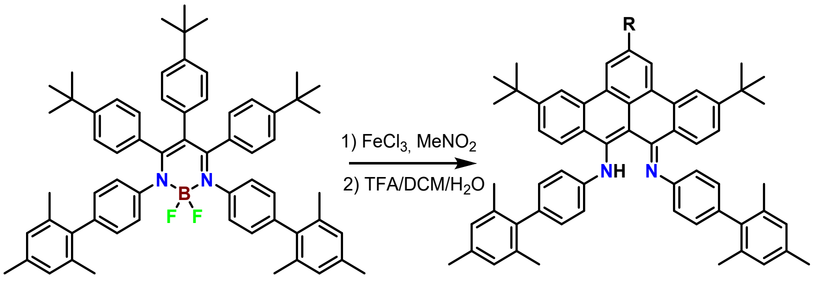

^{TBP}BDI-BF$_2$ (385.5 mg, 0.43 mmol, 1 equiv.) was added to a three-necked, 1 L round-bottom flask equipped with a magnetic stir bar. Then, DCM (390 mL) was added, and the solution was sparged with N_2 gas for 30 minutes. The reaction mixture was protected from light using aluminium foil. $FeCl_3$ (1.59 g, 9.8 mmol, 23 equiv.) was dissolved in $MeNO_2$ (39 mL) and slowly added to the DCM solution under a flow of N_2. The N_2 inlet was then replaced by a stopper and the solution was stirred for approximately 15 hours. Aqueous $NaHCO_3$ (150 mL, saturated) was added to solution, and the solution changed from brown/yellow to orange/red, with the formation of solids. The organics were extracted from the aqueous phase with DCM (150 mL). The organic phase was washed with H_2O (4x150 mL) and brine (2x150 mL), dried on $MgSO_4$ and concentrated under reduced pressure. To the resulting solids, a mixture of trifluoroacetic acid (36 mL), DCM (2 mL) and water (2 mL) was added and left to stir for 10 hours. To the mixture was then slowly added aqueous $NaHCO_3$ (~700 mL, saturated) until the pH was basic, and DCM (100 mL) was added; the organic phase was bright red. The phases were separated and the aqueous phase was extracted with DCM (150 mL). The combined organic phases were washed with water (2x100 mL) and brine (1x100 mL), dried on $MgSO_4$ and concentrated under reduced pressure. Purification was done by column chromatography on silica, using a gradient eluent (3/1 -> 2/1 -> 1/1 -> 0/1 PE/DCM). **^{Cl}BT-BDI** was isolated from this as first fraction. The second fraction, containing **^{tBu}BT-BDI** and starting material (**^{TBP}BDI-BF$_2$**) was then purified by column chromatography on silica (95:5 PE/ethyl acetate). These solids were then washed with cold MeOH (5 mL) and cold HMDSO (5 mL). The combined MeOH and HMDSO washings were put in the freezer to obtain more material over-night, which was combined with the other solids and dried *in vacuo* to obtain **^{tBu}BT-BDI** (44.5 mg, 12 %) and **^{Cl}BT-BDI** (40.2 mg, 11 %) as red to orange solids.

Note: Scholl oxidations were also attempted using combinations of DDQ and methanesulfonic acid, triflic acid or $Sc(OTf)_3$ but gave irreproducible results, and no desired products could be isolated from the crude reaction mixtures.

^{tBu}BT-BDI

^{1}H NMR (400 MHz, CDCl$_3$, 298 K): δ = 13.23 (bs, 1H), 8.64 (s, 2H), 8.47 (s, 2H), 7.81 (d, $^3J_{H,H}$ = 8.7 Hz, 2H), 7.27 (dd, $^3J_{H,H}$ = 8.4, $^4J_{H,H}$ = 1.4 Hz, 2H), 7.03 (d, $^3J_{H,H}$ = 8.5 Hz, 2H), 6.98 (d, $^3J_{H,H}$ = 8.0 Hz, 2H), 6.96 (s, 4H), 2.33 (s, 6H), 2.11 (s, 12H), 1.64 (s, 9H), 1.46 (s, 18H) ppm.

^{13}C{^{1}H} NMR (101 MHz, CDCl$_3$, 298 K): δ = 165.59, 152.29, 147.60, 146.50, 139.16, 136.49, 136.47, 134.70, 134.17, 130.34, 129.90, 129.43, 128.19, 127.29, 125.62, 125.41, 123.79, 119.81, 119.76, 119.54, 35.32, 31.82, 31.37, 21.19, 21.00 ppm.

ATR-IR: ν = 2957 (s), 2909 (m), 2866 (m), 1723 (w), 1591 (m), 1500 (m), 1476 (m), 1415 (m), 1379 (m), 1363 (m), 1313 (m), 1283 (m), 1260 (m), 1241 (m), 1130 (w), 1100 (w), 1034 (w), 1003 (w), 873 (w), 848 (w), 833 (w), 801 (w), 729 (w), 713 (w) cm^{-1}.

HR-MS: m/z = 851.5292 {[M+H]$^+$, calc. 851.5304}.

^{Cl}BT-BDI

^{1}H NMR (400 MHz, CDCl$_3$, 298 K): δ = 13.45 (bs, 1H), 8.51 (s, 2H), 8.37 (d, $^4J_{H,H}$ = 1.2 Hz, 2H), 7.81 (d, $^3J_{H,H}$ = 8.6 Hz, 2H), 7.29 (dd, $^3J_{H,H}$ = 8.8, $^4J_{H,H}$ = 1.4 Hz, 2H), 7.05 (d, $^3J_{H,H}$ = 8.4 Hz, 4H), 6.99 (d, $^3J_{H,H}$ = 8.4 Hz, 4H), 6.96 (s, 4H), 2.34 (s, 6H), 2.11 (s, 12H), 1.45 (s, 18H) ppm.

^{13}C{^{1}H} NMR (101 MHz, CDCl$_3$, 298 K): δ = 152.85, 147.13, 139.03, 136.56, 136.49, 136.43, 135.12, 132.93, 130.43, 130.33, 129.12, 128.21, 125.97, 124.51, 122.39, 119.96, 119.89, 35.39, 31.38, 21.17, 20.97 ppm.

Note: a smaller number of signals than expected is observed with ^{13}C NMR spectroscopy. We attribute this to broadening and low signal intensity observed for several other aromatic signals in the ^{13}C NMR of both this compound and **BT-BDI**.

ATR-IR: ν = 2957 (s), 2867 (m), 1592 (m), 1499 (m), 1476 (m), 1461 (m), 1416 (m), 1379 (m), 1363 (m), 1313 (m), 1284 (m), 1213 (m), 1204 (w), 1130 (w), 1101 (w), 1034 (w), 1013 (w), 959 (w), 931 (w), 907 (w), 873 (w), 833 (w), 801 (w), 731 (w), 634 (w). 614 (w), 574 (w) 521 (w) cm^{-1}.

HR-MS: m/z = 829.4286 {[M+H]$^+$, calc. 829.4289}.

BT-BDI-BF_2

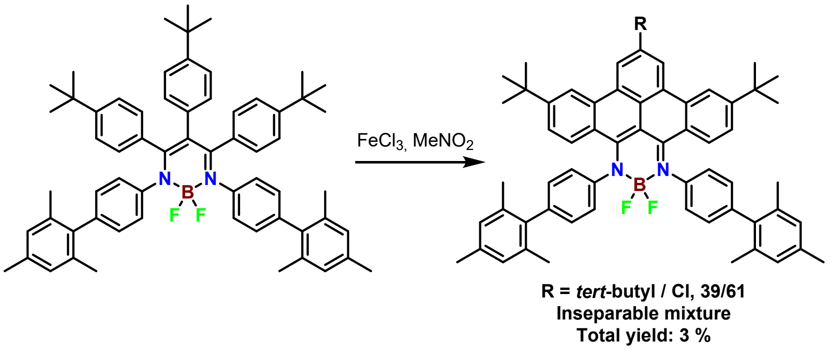

^{TBP}**BDI-BF$_2$** (392.6 mg, 0.43 mmol, 1 equiv.) was added to a three-necked, 1 L round-bottom flask equipped with a magnetic stir bar. Then, DCM (390 mL) was added, and the solution was sparged with N_2 gas for 30 minutes. The reaction mixture was protected from light using aluminium foil. $FeCl_3$ (1.62 g, 10.0 mmol, 23 equiv.) was dissolved in $MeNO_2$ (39 mL) and dropwise added to the DCM solution under a flow of N_2. The N_2 inlet was then replaced by a stopper and the solution was stirred for approximately 15 hours. Aqueous $NaHCO_3$ (150 mL, saturated) was added to solution, and the solution changed from brown/yellow to orange/red with the formation of solids. The organics were extracted from the aqueous phase with DCM (150 mL). The organic phase was washed with H_2O (4x150 mL) and brine (2x150 mL), dried on $MgSO_4$ and concentrated under reduced pressure. The solids were extracted with PE (~200 mL), concentrated under reduced pressure, and purified by column chromatography on silica using a gradient eluent (95/5 -> 90/10 PE/ethyl acetate) and the second fraction, containing **BT-BDI-BF$_2$** and starting material ^{TBP}**BDI-BF$_2$** was collected. Further purification was done by column chromatography on basic alumina (98/2 PE/ethyl acetate). The first fraction containing **BT-BDI-BF$_2$** was concentrated under reduced pressure and the resulting solids were washed with cold MeOH (5 mL) and cold HMDSO (5 mL). The combined MeOH and HMDSO washings were put in the freezer to obtain more material over-night, which was combined with the other solids and dried *in vacuo* to obtain **BT-BDI-BF$_2$** as a dark red solid, consisting of an inseparable mixture of the two related compounds (R=*t*Bu/Cl 39/61) shown above (13.2 mg, 3 %).

Note: For this particular reaction, the ratio of R = *t*Bu and Cl was 39/61 as observed by integration of the peaks between 9 and 8 ppm in the ^{1}H NMR spectrum. However, this ratio varies slightly amongst different samples.

^{1}H NMR (400 MHz, $CDCl_3$, 298 K): δ = 8.68 (R=*t*Bu, s, 2H), 8.56 (R=Cl, s, 2H), 8.45 (R=*t*Bu, d, $^4J_{H,H}$ = 2.0 Hz, 2H), 8.35 (R=Cl, d, $^4J_{H,H}$ = 2.0 Hz, 2H), 7.74 (dd, $^3J_{H,H}$ = 8.9, $^4J_{H,H}$ = 2.0 Hz, 4H), 7.72 (d, $^3J_{H,H}$ = 8.1 Hz, 8H), 7.17 – 7.05 (m, 12H), 6.94 (s, 8H), 2.32 (s, 12H), 2.05 (s, 24H), 1.62 (R=*t*Bu, s, 9H), 1.41 (s, 18H), 1.40 (s, 18H) ppm.

^{13}C{^{1}H} NMR (101 MHz, $CDCl_3$, 298 K): δ = 155.3, 154.5, 153.9, 146.3, 144.5, 144.2, 139.4, 139.1, 138.7, 138.5, 136.9, 136.8, 136.6, 136.1, 135.2, 131.3, 130.4, 130.2, 130.0, 128.2, 128.1, 127.5, 126.2, 124.2, 123.9, 123.8, 123.6, 123.1, 120.5, 119.9, 119.6, 35.5, 35.4, 35.3, 31.9, 31.1, 21.2, 20.8 ppm.

^{19}F NMR (376 MHz, $CDCl_3$, 298 K): δ = -124.8 (q, $^1J_{B,F}$ = 26.8 Hz) ppm.

^{11}B NMR (128 MHz, $CDCl_3$, 298 K): δ = 0.78 (t, $^1J_{B,F}$ = 25.6 Hz) ppm.

ATR-IR: v = 2958 (s), 2920 (m), 2866 (m), 1610 (m), 1567 (m), 1507 (m), 1495 (m), 1477 (m), 1456 (s), 1413 (s), 1388 (s), 1363 (s), 1331 (m), 1288 (w), 1260 (m), 1238 (m), 1202 (w), 1133 (w), 1103 (w), 1008 (m), 847 (w), 832 (w), 800 (w) cm^{-1}.

ESI-MS (MeCN): No signal observed.

^{tBu}BT-BDI-ZnEt

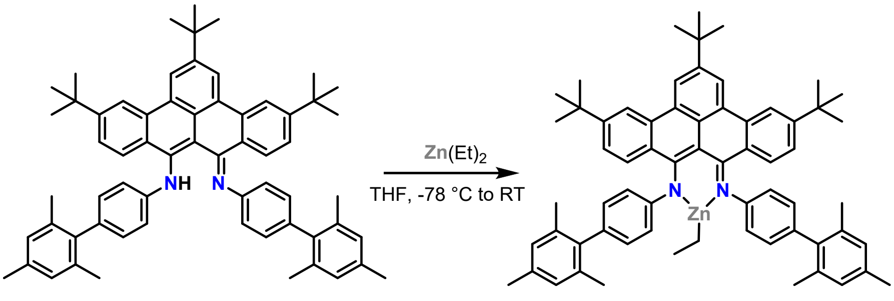

To a solution of ^{tBu}**BT-BDI** (19.8 mg, 24 µmol, 1 equiv.) in THF (~1.5 mL) was added $Zn(Et)_2$ (1 M in hexanes, 28 µL, 28 µmol, 1.2 equiv.) at -78 °C. After stirring at this temperature for ~10 minutes, the mixture was warmed up to room temperature, with an accompanying color change to dark brown, which gradually changed to dark blue. After stirring for 2.5 hours, all volatiles were removed *in vacuo* to obtain ^{tBu}**BT-BDI-ZnEt** as a dark purple solid (20.1 mg, 91 %).

^{1}H NMR (400 MHz, $CDCl_3$, 298 K): δ = 9.00 (s, 2H), 8.69 (d, $^{4}J_{H,H}$ = 2.1 Hz, 2H), 8.14 (d, $^{3}J_{H,H}$ = 8.8 Hz, 2H), 7.16 (dd, $^{3}J_{H,H}$ = 8.8 Hz, $^{4}J_{H,H}$ = 2.1 Hz, 2H), 7.03 (d, $^{3}J_{H,H}$ = 8.6 Hz, 4H), 6.93 (d, $^{3}J_{H,H}$ = 8.6 Hz, 4H), 6.91 (bs, 4H), 2.23 (s, 6H), 2.17 (s, 12H), 1.63 (s, 4H), 1.50 (t, $^{3}J_{H,H}$ = 8.1 Hz, 3H), 1.33 (s, 18H), 0.92 (q, $^{3}J_{H,H}$ = 8.0 Hz, 2H) ppm.

^{13}C{^{1}H} NMR (101 MHz, $CDCl_3$, 298 K): δ = 162.07, 153.53, 152.32, 144.87, 139.34, 136.34, 136.11, 136.05, 135.57, 132.12, 130.69, 128.61, 128.39, 127.72, 126.63, 126.07, 124.14, 123.27, 120.33, 119.45, 115.23, 35.13, 35.10, 31.86, 31.13, 21.19, 20.99, 12.90, 0.43 ppm.

ATR-IR: ν = 2960.1 (s), 2863.0 (m), 2731.3 (w), 1608.5 (m), 1568.3 (m), 1548.2 (m), 1477.8 (s), 1438.1 (m), 1404.28 (m), 1358.8 (s), 1331.4 (s), 1313.1 (m), 1285.0 (m), 1256.7 (s), 1205.9 (w), 1177.7 (w), 1140.2 (w), 1100.1 (m), 1022.8 (m), 1004.0 (w), 971.4 (w), 933.3 (w), 873.4 (w), 849.8 (w), 829.8 (w), 819.1 (w), 799.7 (w), 743.2 (w), 712.5 (w), 573.4 (w) cm^{-1}.

Elemental analysis: Anal. Calcd. for $C_{65}H_{70}N_2Zn$: C, 82.64; H, 7.47; N, 2.97 %. Found: C, 76.88; H, 6.83; N, 2.79 %. A satisfactory elemental analysis could not be obtained due to the sensitivity of the complex.

^{TBP}Indole·HMeSO$_3$

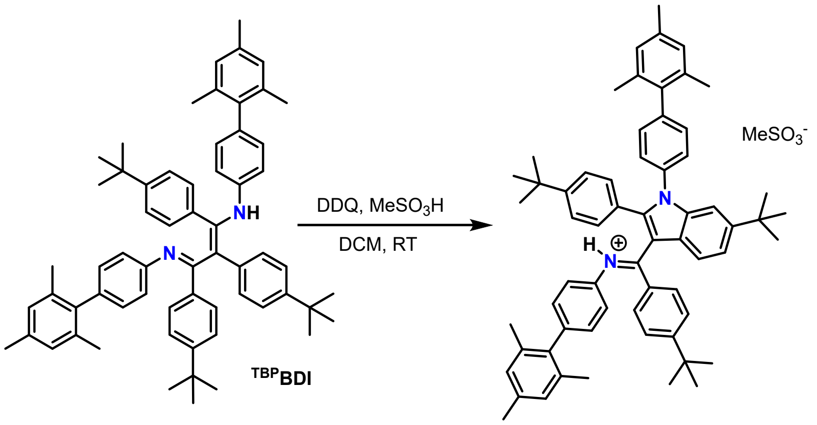

^{tBu}Indole•HMeSO$_3$

A Schlenk flask was charged with **^{TBP}BDI** (91.7 mg, 0.11 mmol, 1 equiv.) and DCM (10 mL), after which methanesulfonic acid (1.5 mL) was added. Upon addition, the colour changed from orange do dark red. After stirring at room temperature for 15 minutes, DDQ (105 mg, 0.46 mmol, 4.2 equiv.) was added, upon which the colour changed to brown. After stirring for an additional 10 minutes, the colour changed to orange, with the formation of a suspension. After total of 1 hour since the initial addition of methanesulfonic acid, water (20 mL) was added. The organic phase was separated from the aqueous phase and washed with additional water (20 mL). The organic phase was dried on $MgSO_4$ and concentrated under reduced pressure to yield a brown solid (89.7 mg). The presented spectroscopic data is from this sample without further purification and shows full conversion to **^{TBP}Indole·HMeSO$_3$** (see note). After washing with petroleum ether (10 mL), crystals suitable for single-crystal XRD were grown from a solution of diethyl ether and pentane.

^{1}H NMR (400 MHz, CDCl$_3$, 298 K): δ = 13.48 (s, 1H), 7.94 (d, $^3J_{H,H}$ = 8.5 Hz, 2H), 7.50 (d, $^3J_{H,H}$ = 8.8 Hz, 2H), 7.28 – 7.16 (m, 8H), 7.07 (d, $^3J_{H,H}$ = 8.7 Hz, 2H), 6.98 – 6.86 (m, 7H), 6.77 (d, $^3J_{H,H}$ = 8.6 Hz, 2H), 2.33 (s, 3H), 2.29 (s, 3H), 2.22 (s, 3H), 1.97 (bs, 6H), 1.91 (s, 6H), 1.33 (s, 9H), 1.32 (s, 9H), 1.18 (s, 9H) ppm.

^{13}C{^{1}H} NMR (101 MHz, CDCl$_3$, 298 K): δ = 172.1, 160.3, 152.8, 148.9, 148.6, 142.2, 141.4, 139.0, 137.7, 137.6, 137.5, 137.4, 137.2, 135.8, 135.7, 134.6, 132.8, 130.3, 130.1, 128.5, 128.4, 126.4, 125.9, 125.6, 124.0, 123.9, 122.1, 120.6, 107.8, 107.2, 38.9, 35.7, 35.2, 34.7, 31.6, 31.1, 21.2, 21.1, 20.8 ppm.

ESI-MS (MeCN): m/z = 853.6 {[M+H]$^+$, calc. 853.6}.

Note: We include the synthesis of this compound because it explains the reasoning behind the development of a templated strategy for our target molecules as presented in the manuscript. However, the compound itself is not of further interest to our goals in this study. We therefore include limited analytical data on this compound, nor do we report on the further purification of this compound beyond what we obtained from the crude reaction mixture after aqueous washing. The spectroscopic data presented allows us to corroborate that the crystal structure corresponds to the bulk of the material, with the exception of the co-crystallised DDQ, which does not appear to be present in significant quantities based on the ^{13}C NMR. Furthermore, toluene, which was not used as a solvent in the procedure or for the crystallisation, is found to be co-crystallised, which is due to residual solvent from previous crystallisation attempts.

Attempted Scholl oxidation of ^{TBP}BDI using $FeCl_3$

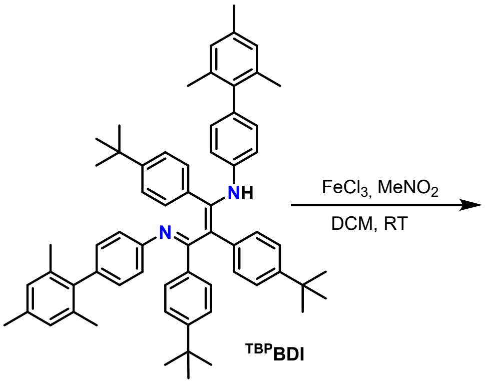

^{TBP}BDI (101.6 mg, 0.12 mmol, 1 equiv.) was added to a three-necked round-bottom flask equipped with a magnetic stir bar. Then, DCM (100 mL) was added, and the solution was sparged with N_2 gas for 30 minutes. The reaction mixture was protected from light using aluminium foil. $FeCl_3$ (443 mg, 2.7 mmol, 23 equiv.) was dissolved in $MeNO_2$ (10 mL) and dropwise added to the DCM solution under a flow of N_2. The N_2 inlet was then replaced by a stopper and the solution was stirred for approximately 16 hours. Aqueous $NaHCO_3$ (50 mL, saturated) was added to solution to quench the reaction. The organics were extracted from the aqueous phase with DCM (50 mL). The organic phase was washed with H_2O (4x50 mL) and brine (2x50 mL), dried on $MgSO_4$ and concentrated under reduced pressure to give a yellow solid.

--

The crude reaction mixture was analyzed using ^{1}H NMR spectroscopy and ESI-MS. The ^{1}H NMR showed minor peaks in the downfield region (8-9 ppm) where the **BT-BDI** has characteristic peaks, indicative of possible conversion towards the desired product (Figure S56). ESI-MS of the crude product confirmed this, with a weak signal at m/z = 851.5 (for **^{tBu}BT-BDI**: $[M+H]^+$, calc. 851.5), as well as a strong signal at 833.4, which could correspond to a similar *tert*-butyl-for-chloride substituted product as observed in the Scholl oxidation of **^{TBP}BDI-BF$_2$**, but without the formation of new carbon-carbon bonds (for **^{TBP}BDI**: $[M + H - C_4H_9 + Cl]^+$, calc. 833.5). These results combined show that Scholl oxidation of the untemplated BDI (**^{TBP}BDI**) is relatively unsuccessful compared to the Scholl oxidation of the templated BDI (**^{TBP}BDI-BF$_2$**). No further attempts at isolating the obtained materials were made.

S3. NMR spectra

1,2-bis(4-(tert-butyl)phenyl)ethan-1-one (^{tBu}DOB)

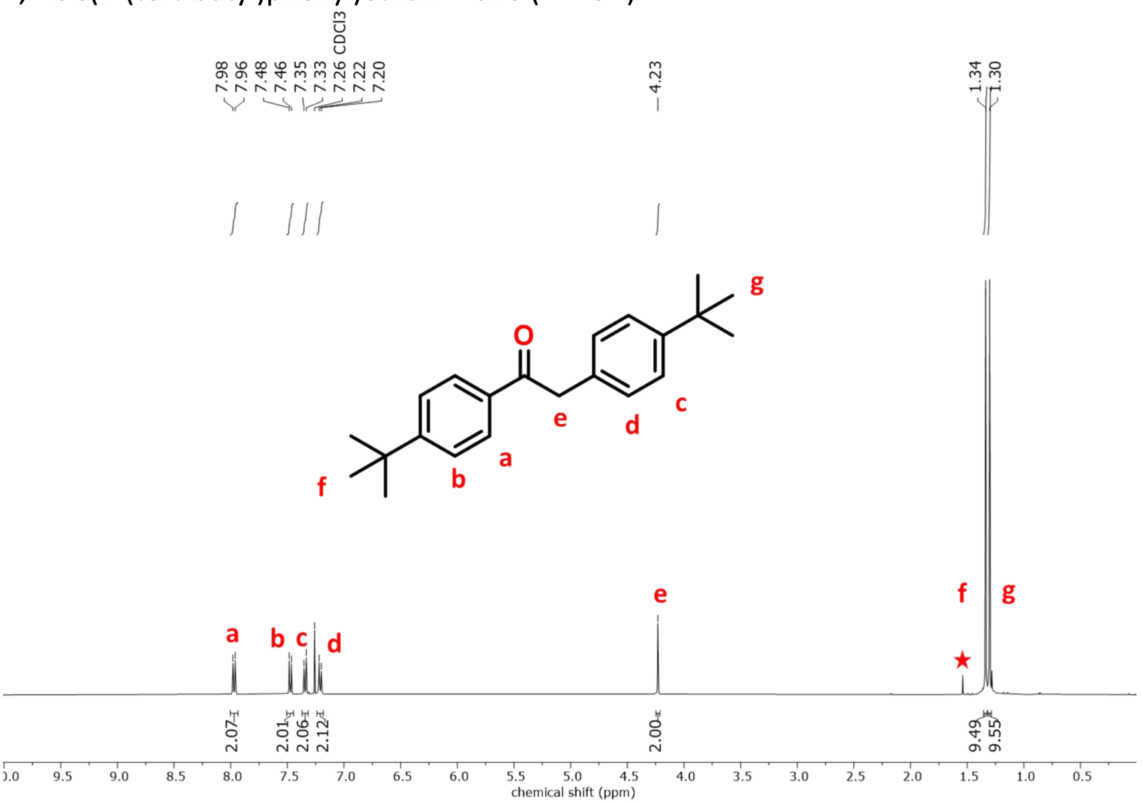

Figure S1: ^{1}H NMR spectrum of **1,2-bis(4-(tert-butyl)phenyl)ethan-1-one** (**^{tBu}DOB)** in $CDCl_3$, at 25 °C. The resonance marked with a star is attributed to residual water in the solvent.

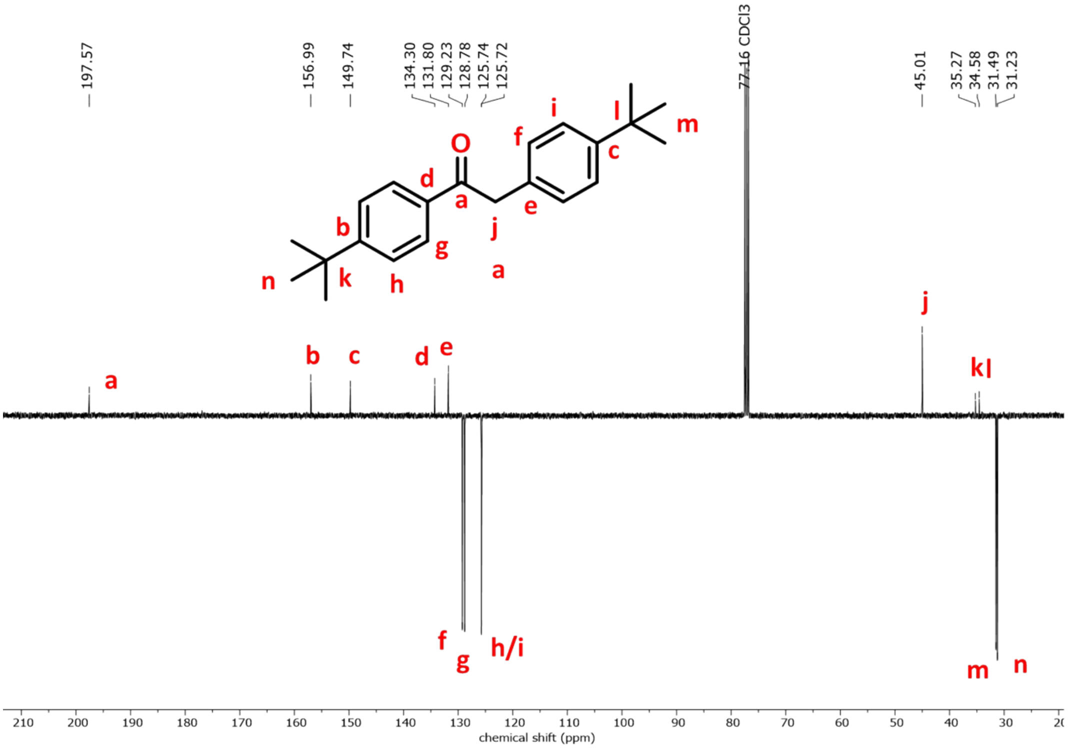

Figure S2: ^{13}C-APT NMR spectrum of **1,2-bis(4-(tert-butyl)phenyl)ethan-1-one** (**^{tBu}DOB)** in $CDCl_3$, at 25 °C.

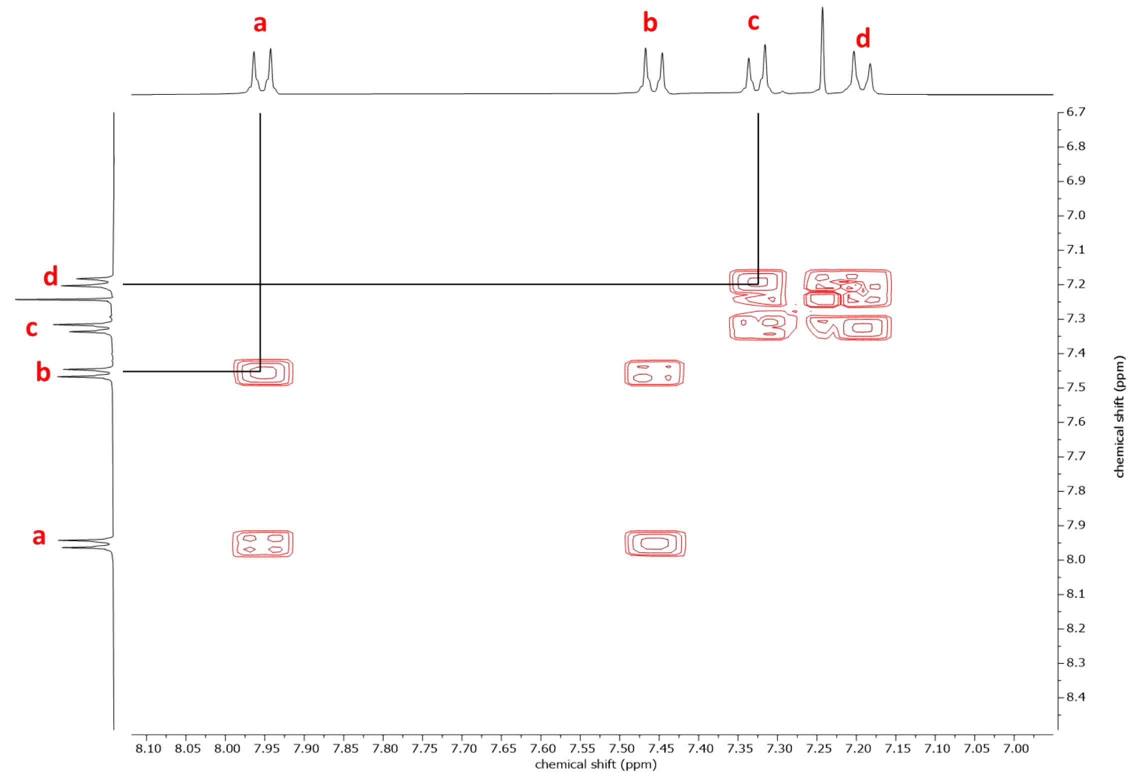

Figure S3: gCOSY NMR spectrum of **1,2-bis(4-(tert-butyl)phenyl)ethan-1-one** (^{tBu}**DOB)** in $CDCl_3$, at 25 °C.

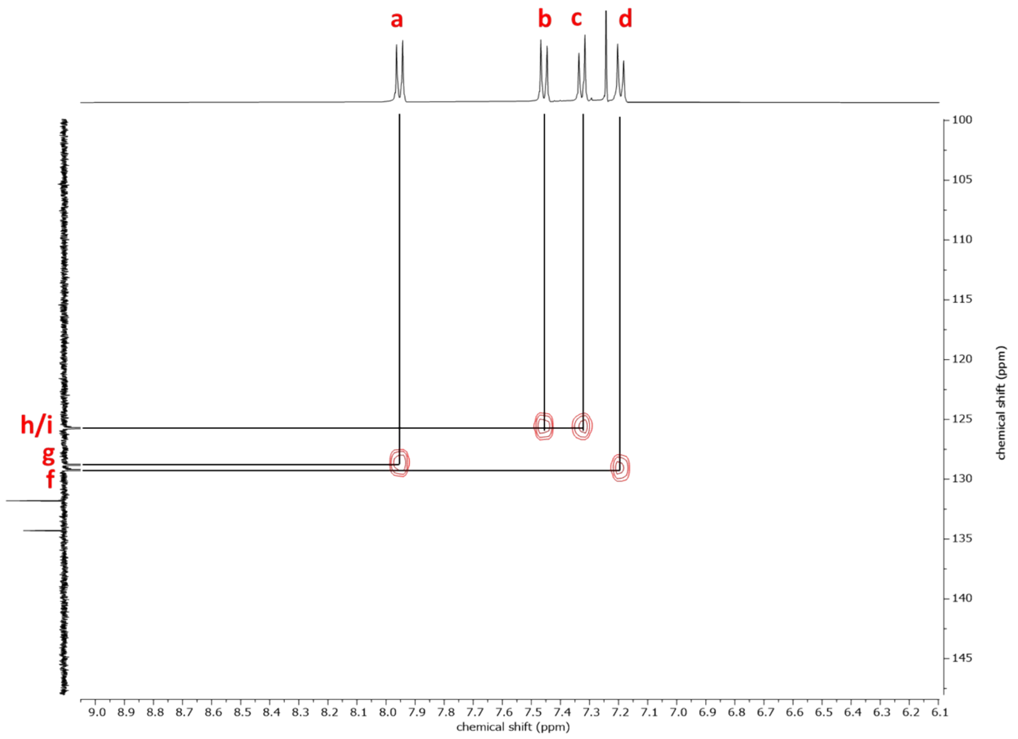

Figure S4: ^{1}H-^{13}C HSQCAD NMR spectrum of **1,2-bis(4-(tert-butyl)phenyl)ethan-1-one** (^{tBu}**DOB)** in $CDCl_3$, at 25 °C.

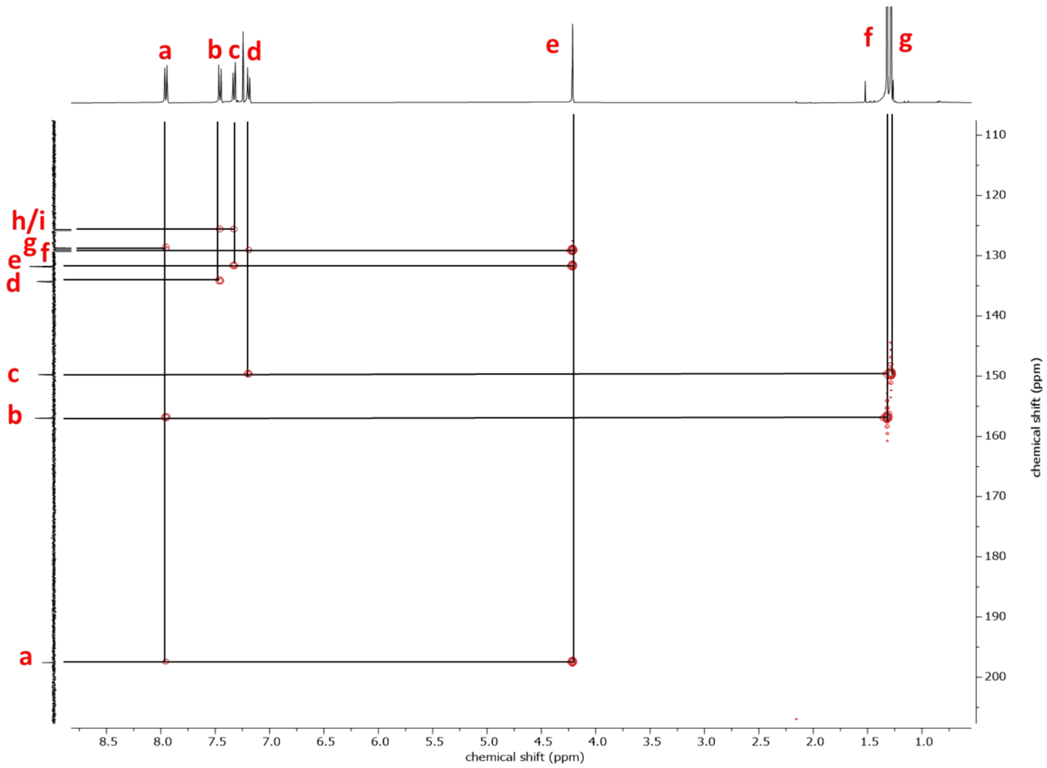

Figure S5: ^{1}H-^{13}C gHMBC NMR spectrum of **1,2-bis(4-(tert-butyl)phenyl)ethan-1-one (^{tBu}DOB)** in $CDCl_3$, at 25 °C.

4-mesityl aniline

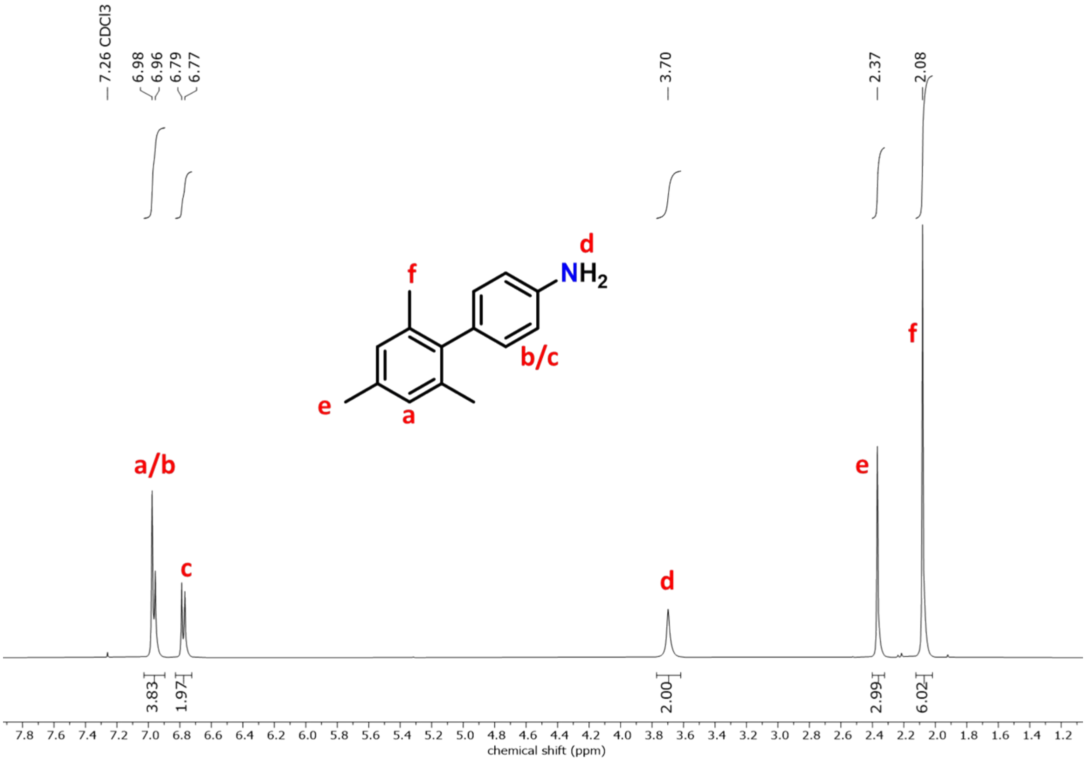

Figure S6: ^{1}H NMR spectrum of **4-mesityl aniline** in $CDCl_3$, at 25 °C.

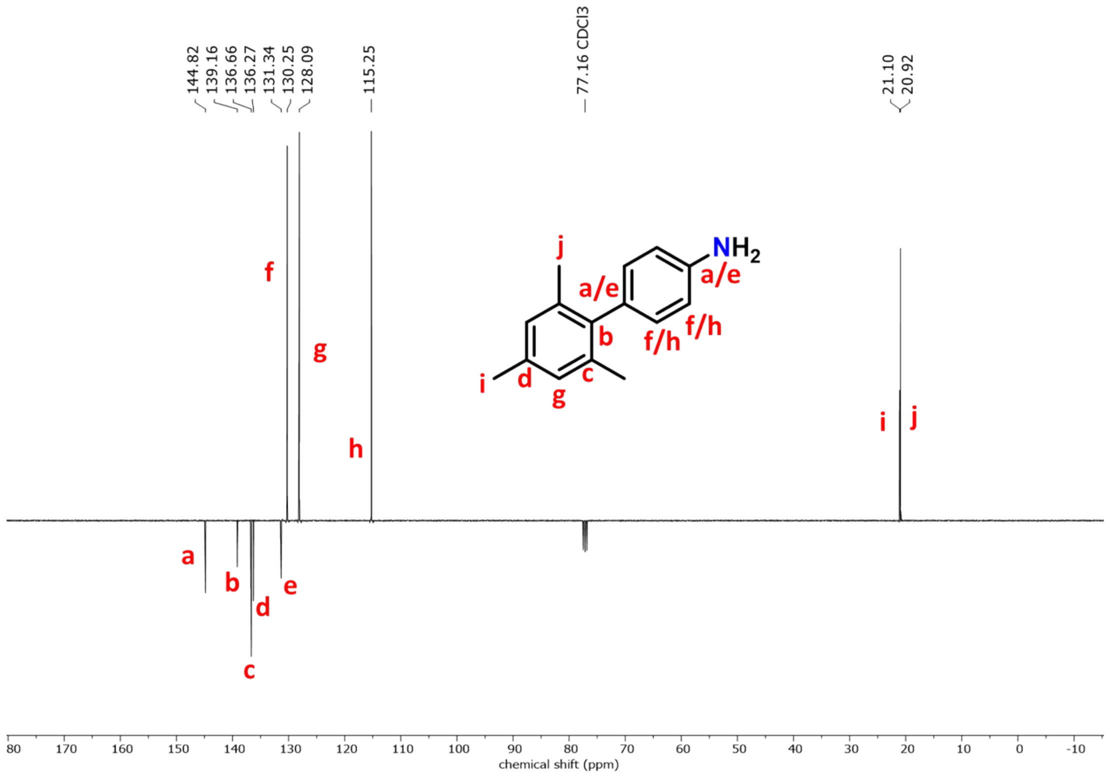

Figure S7: ^{13}C-APT NMR spectrum of **4-mesityl aniline** in $CDCl_3$, at 25 °C.

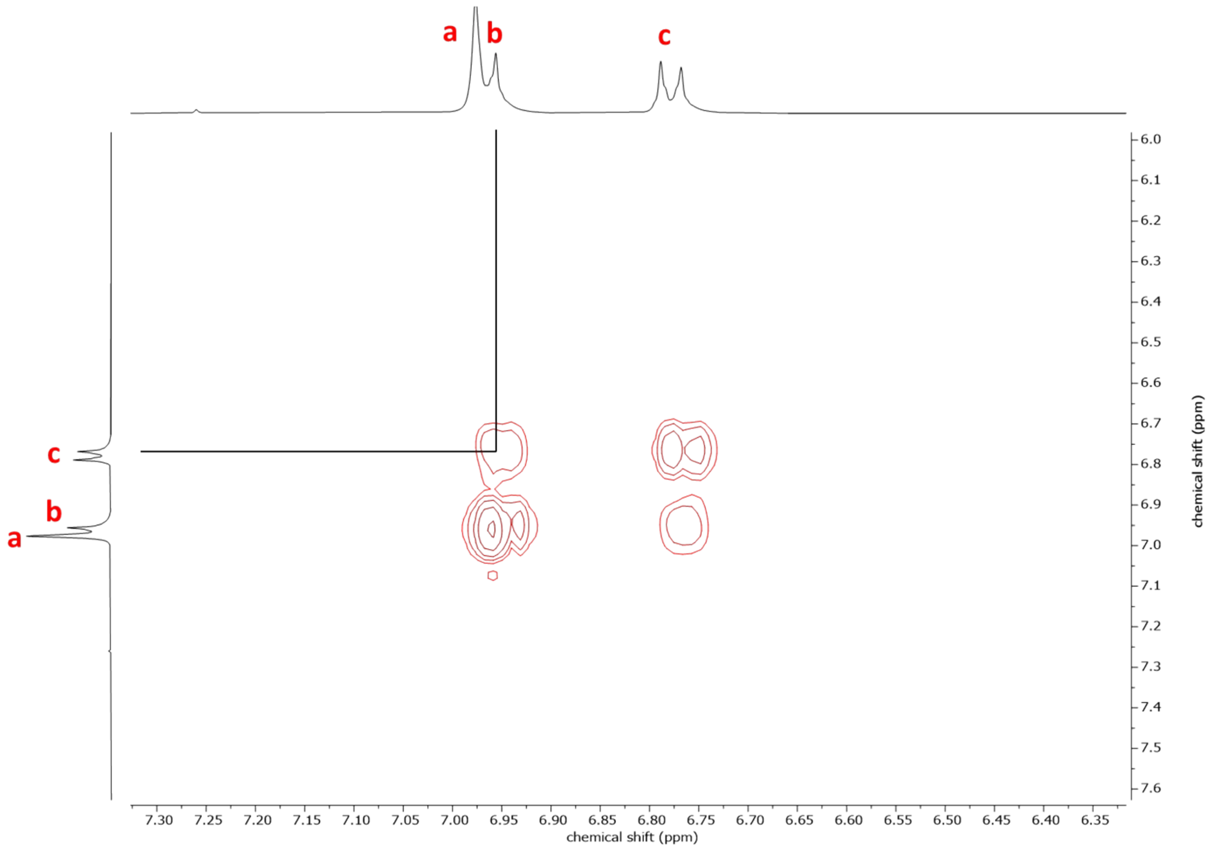

Figure S8: COSY NMR spectrum of **4-mesityl aniline** in $CDCl_3$, at 25 °C.

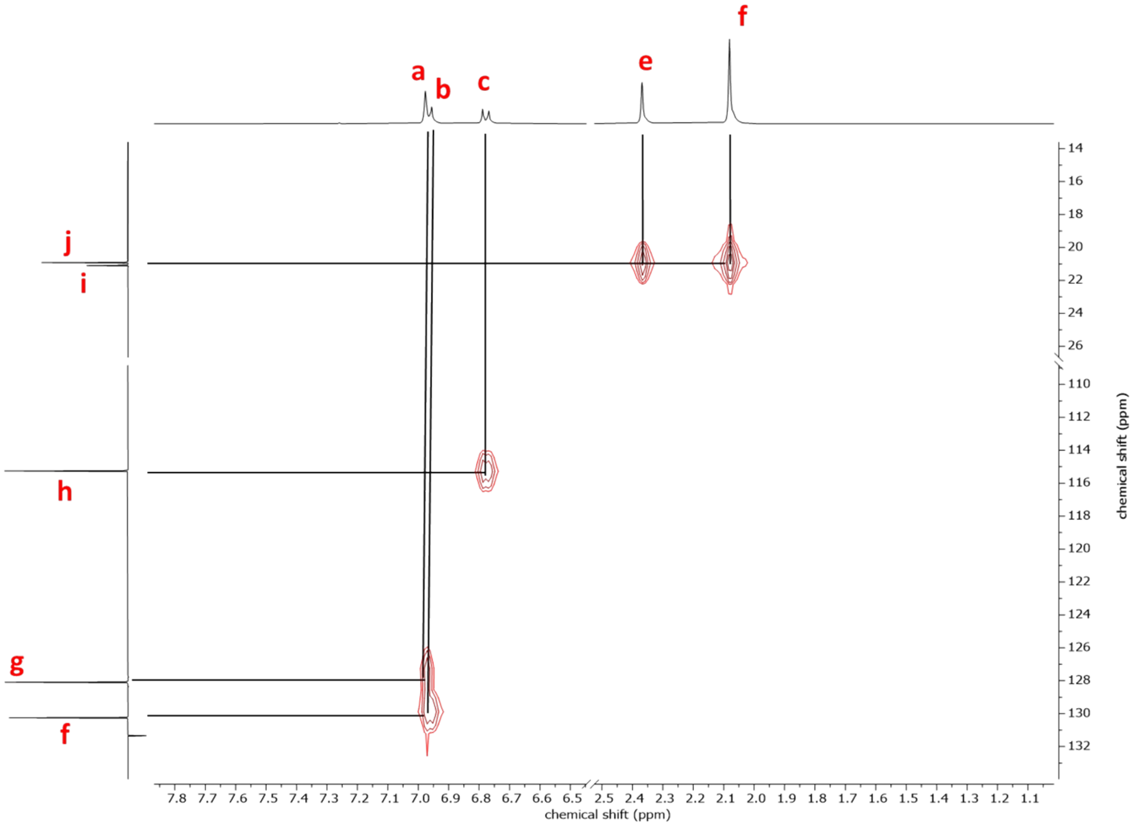

Figure S9: ^{1}H-^{13}C HSQC NMR spectrum of **4-mesityl aniline** in $CDCl_3$, at 25 °C.

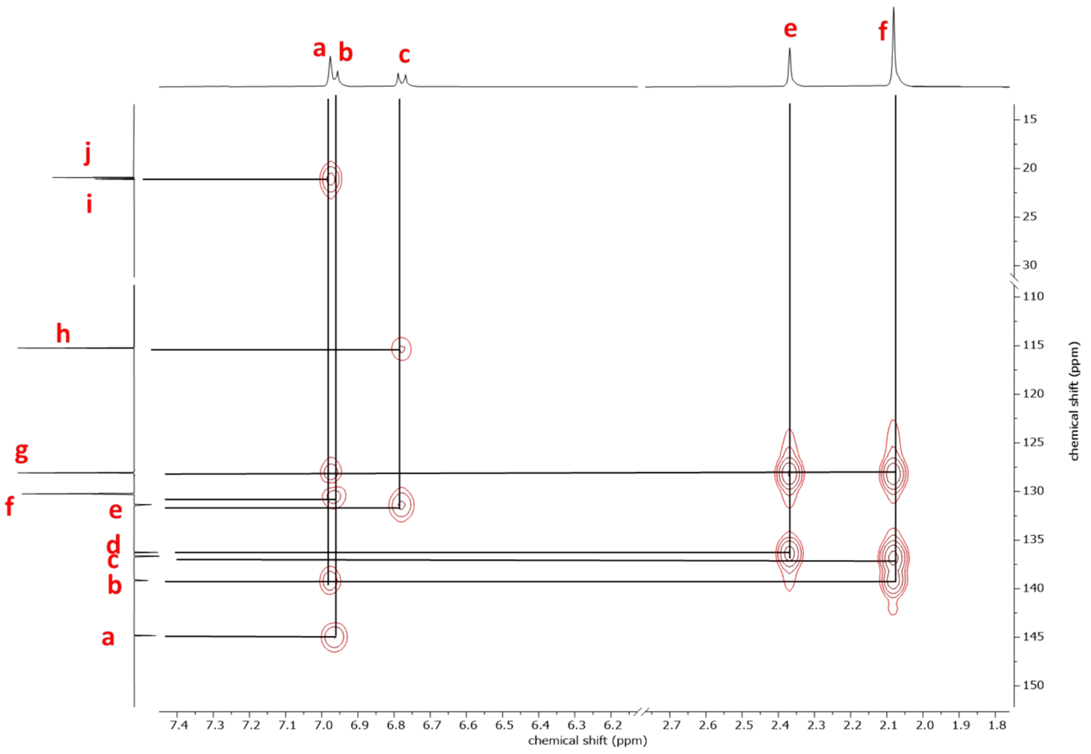

Figure S10: ^{1}H-^{13}C HMBC NMR spectrum of **4-mesityl aniline** in $CDCl_3$, at 25 °C.

1,2,3-tris(4-(tert-butyl)phenyl)propane-1,3-dione (^{TBP}AcAc)

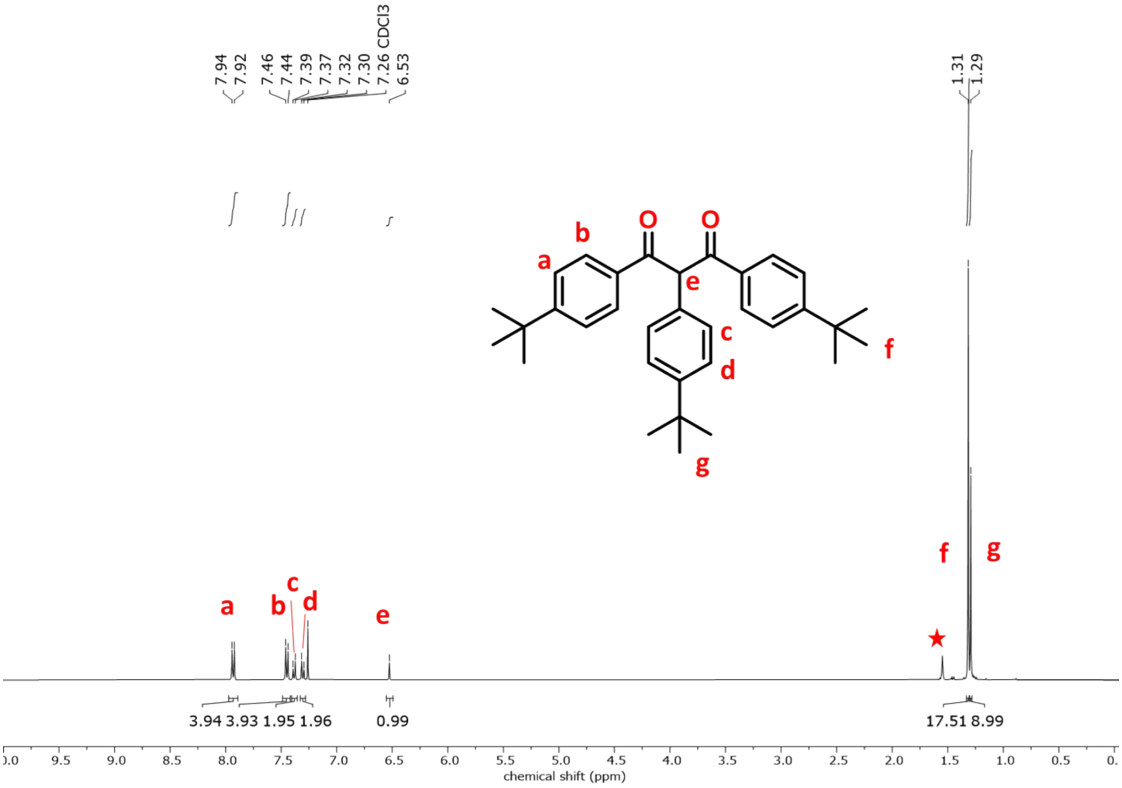

Figure S11: ^{1}H NMR spectrum of **1,2,3-tris(4-(tert-butyl)phenyl)propane-1,3-dione** (^{TBP}**AcAc**) in $CDCl_3$, at 25 °C. The resonance marked with a star is attributed to residual water in the solvent.

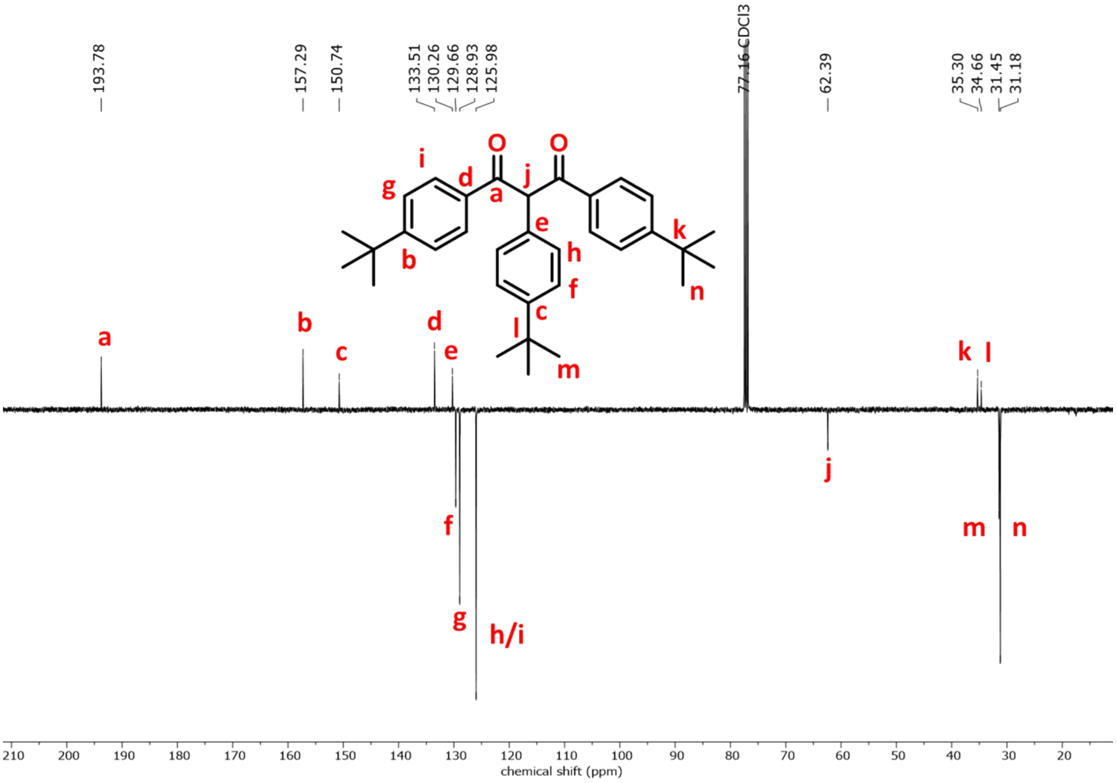

Figure S12: ^{13}C-APT NMR spectrum of **1,2,3-tris(4-(tert-butyl)phenyl)propane-1,3-dione** (^{TBP}**AcAc**) in $CDCl_3$, at 25 °C.

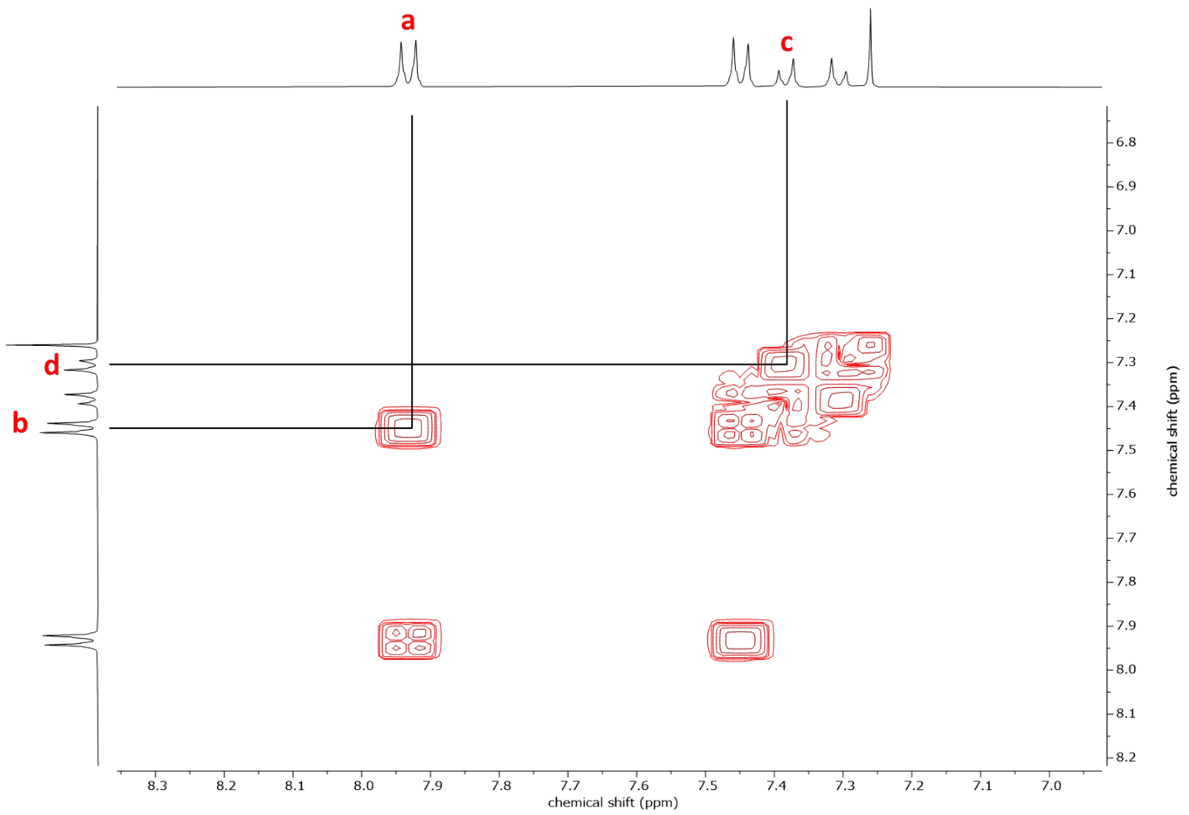

Figure S13: gCOSY NMR spectrum of **1,2,3-tris(4-(tert-butyl)phenyl)propane-1,3-dione** (^{TBP}**AcAc**) in $CDCl_3$, at 25 °C.

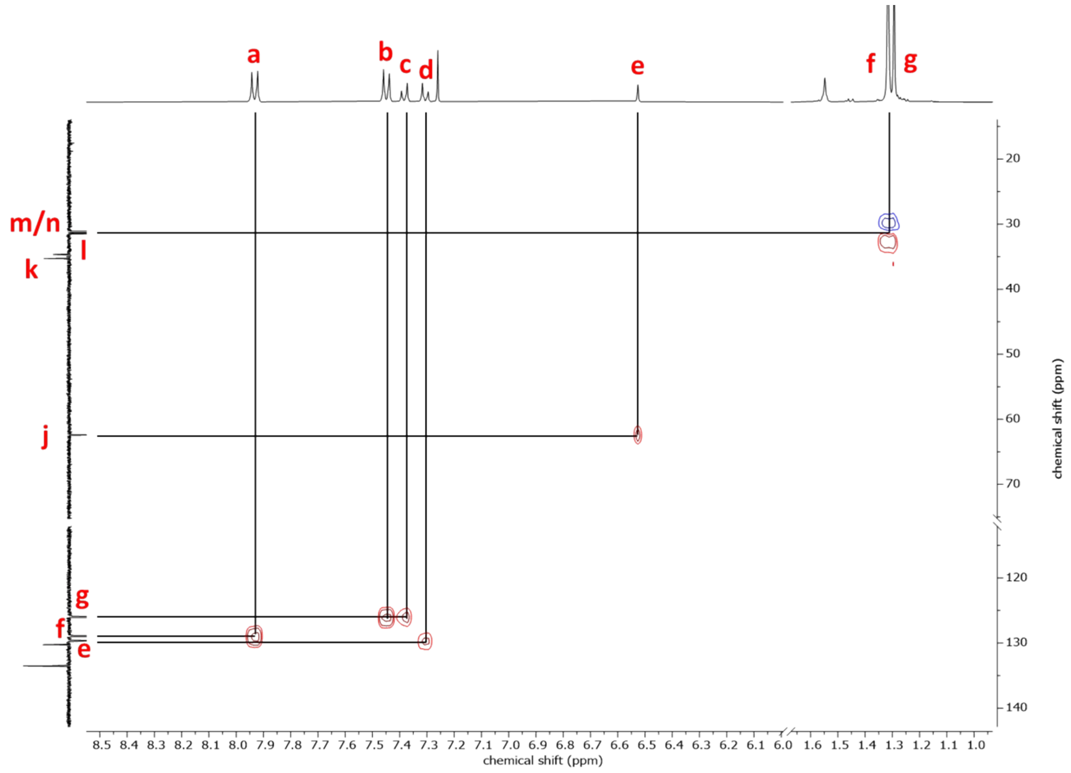

Figure S14: HSQCAD NMR spectrum of **1,2,3-tris(4-(tert-butyl)phenyl)propane-1,3-dione** (^{TBP}**AcAc**) in $CDCl_3$, at 25 °C.

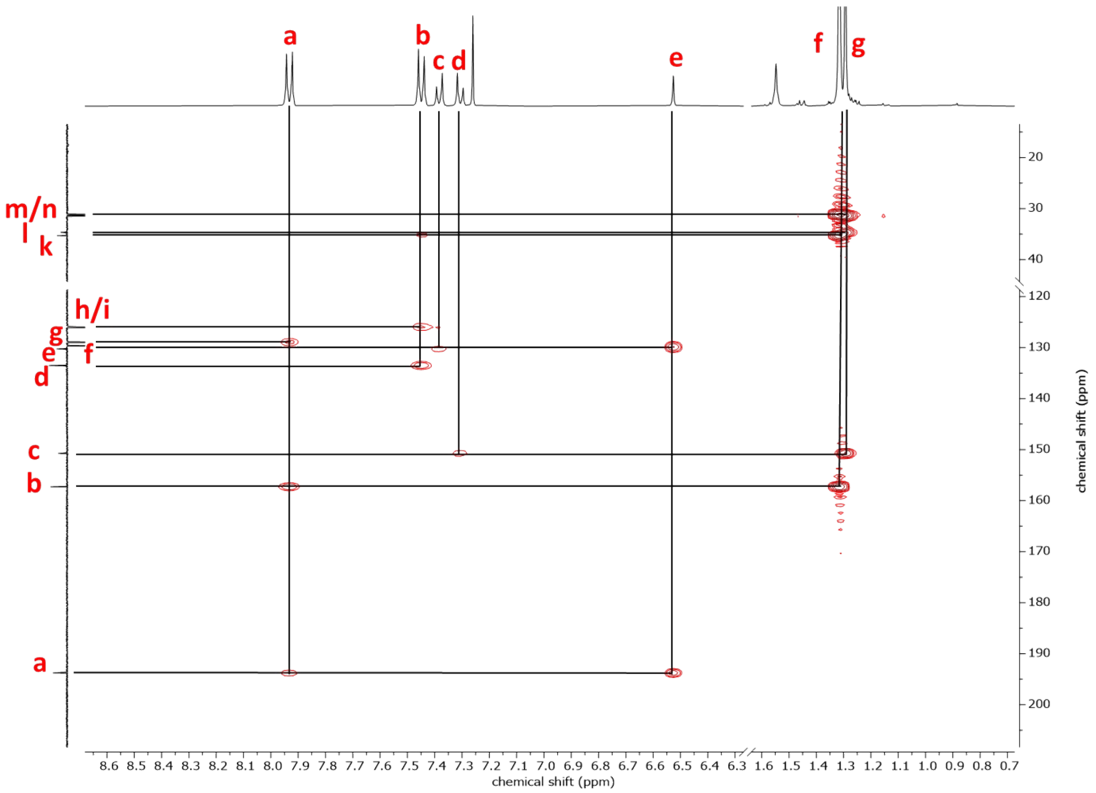

Figure S15: gHMBCAD NMR spectrum of **1,2,3-tris(4-(tert-butyl)phenyl)propane-1,3-dione** (^{TBP}**AcAc**) in $CDCl_3$, at 25 °C.

^{TBP}BDI

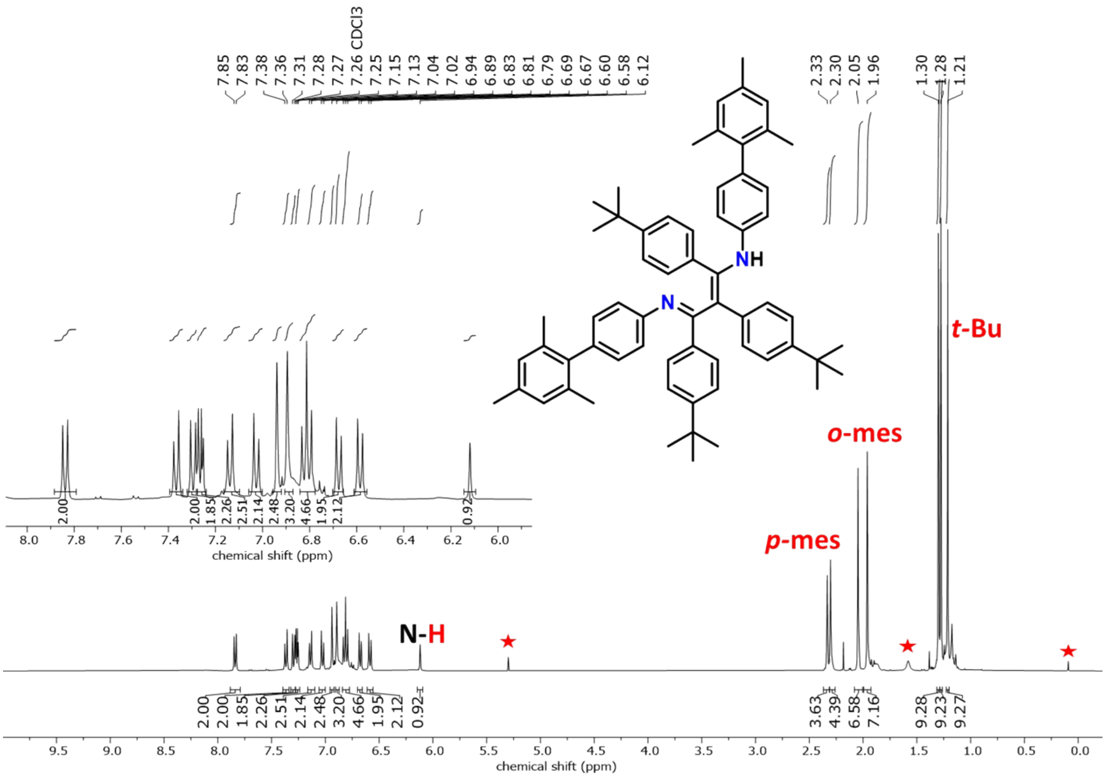

Figure S16: ^{1}H NMR spectrum of ^{TBP}**BDI** in $CDCl_3$, at 25 °C. The resonance marked with a star are attributed to residual DCM (5.30 ppm), water (1.56 ppm) and laboratory grease (0.07 ppm).

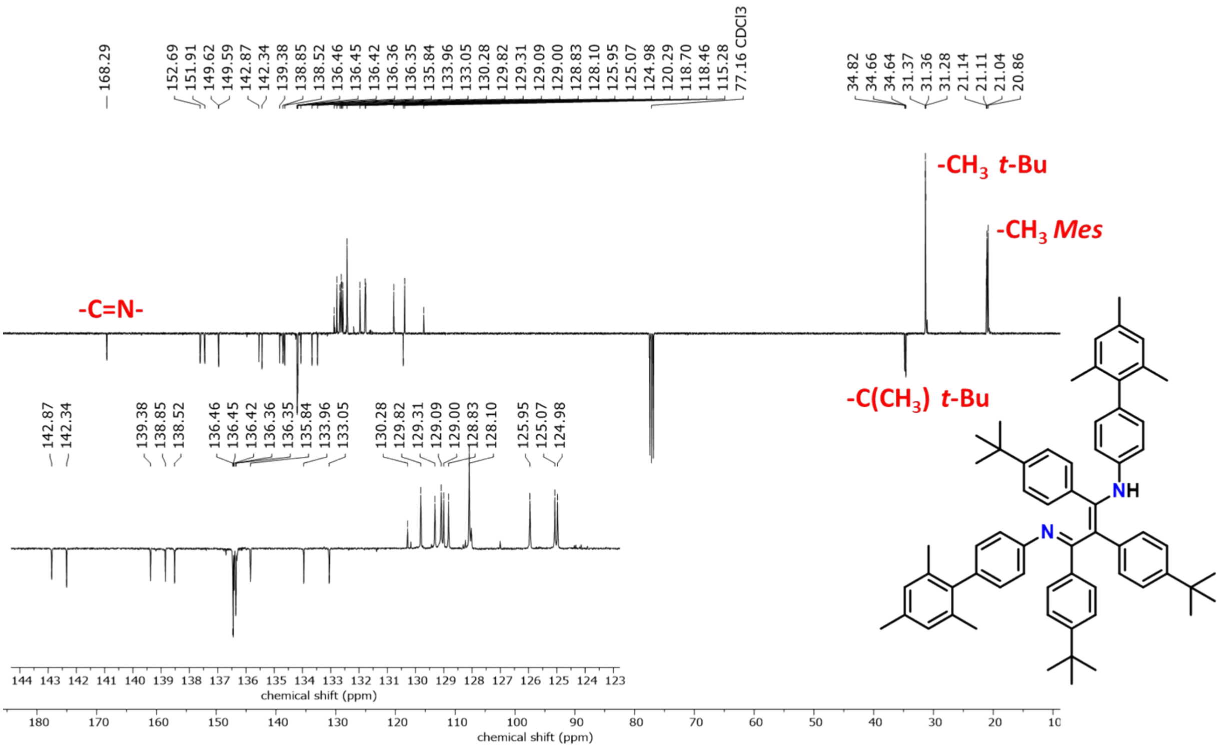

Figure S17: ^{13}C-APT NMR spectrum of **^{TBP}BDI** in $CDCl_3$, at 25 °C.

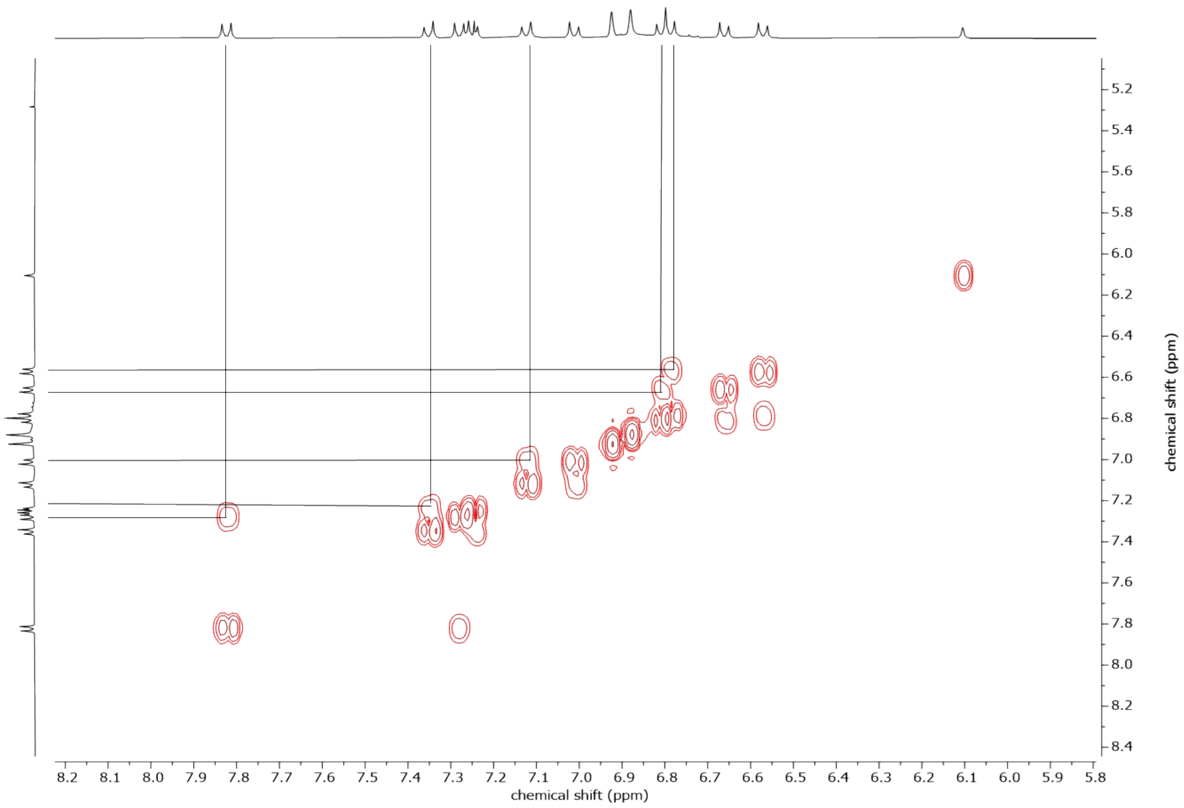

Figure S18: COSY NMR spectrum of **^{TBP}BDI** in $CDCl_3$, at 25 °C.

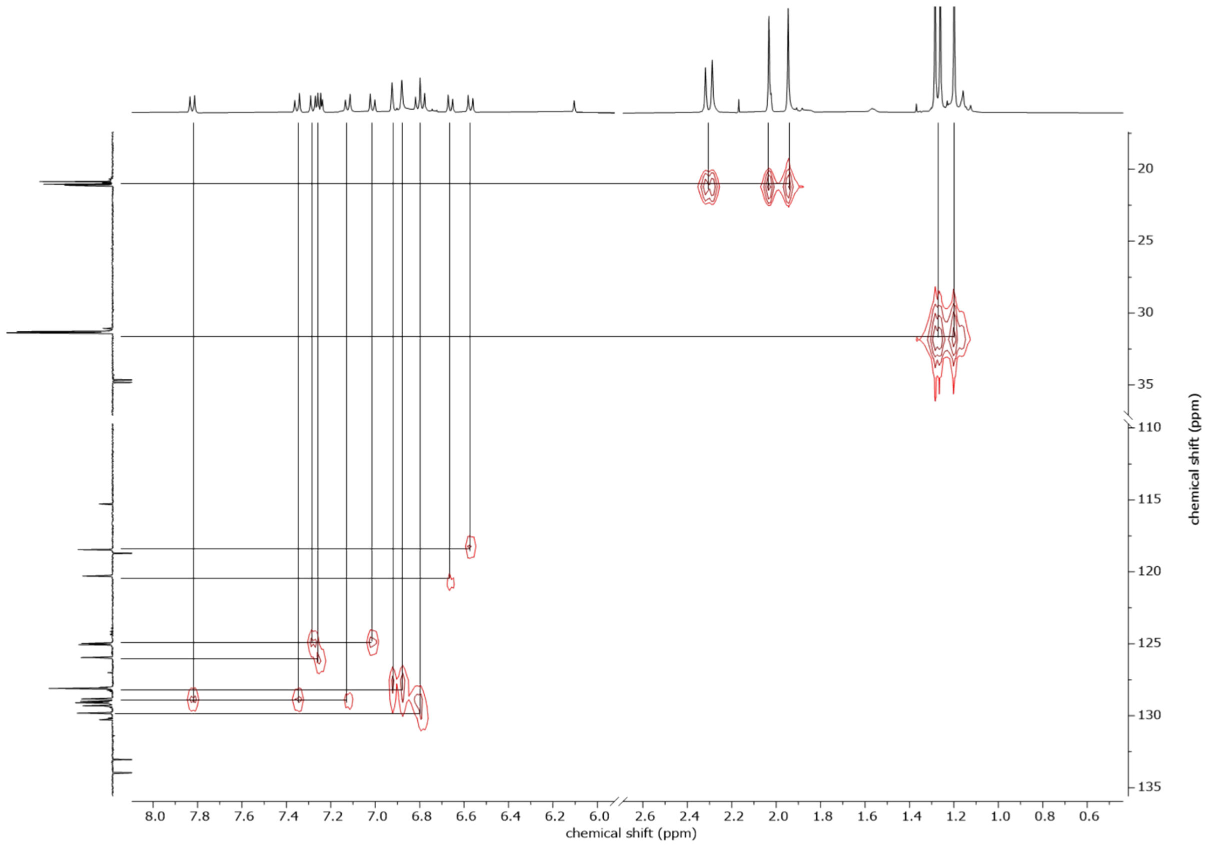

Figure S19: ^{1}H-^{13}C HSQC NMR spectrum of **^{TBP}BDI** in $CDCl_3$, at 25 °C.

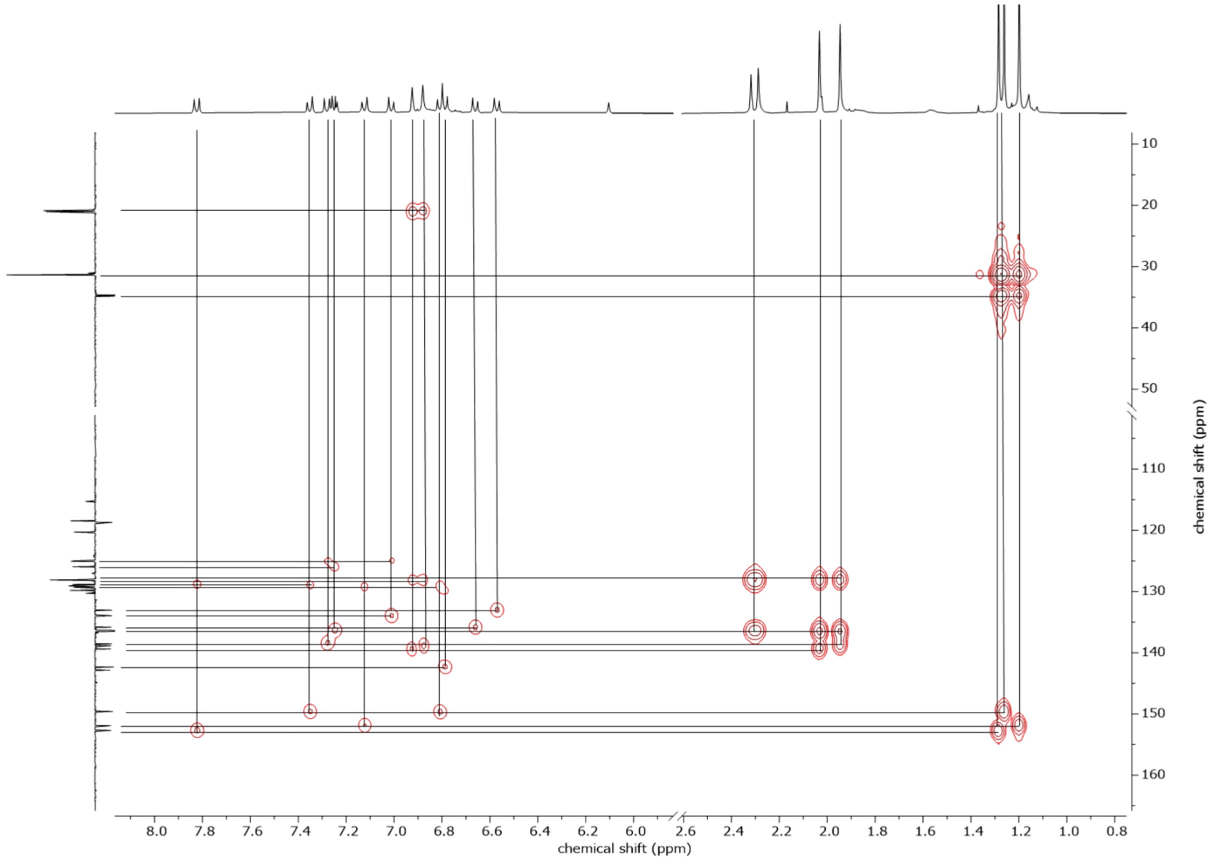

Figure S20: ^{1}H-^{13}C HMBC NMR spectrum of **^{TBP}BDI** in $CDCl_3$, at 25 °C.

^{TBP}BDI-BF$_2$

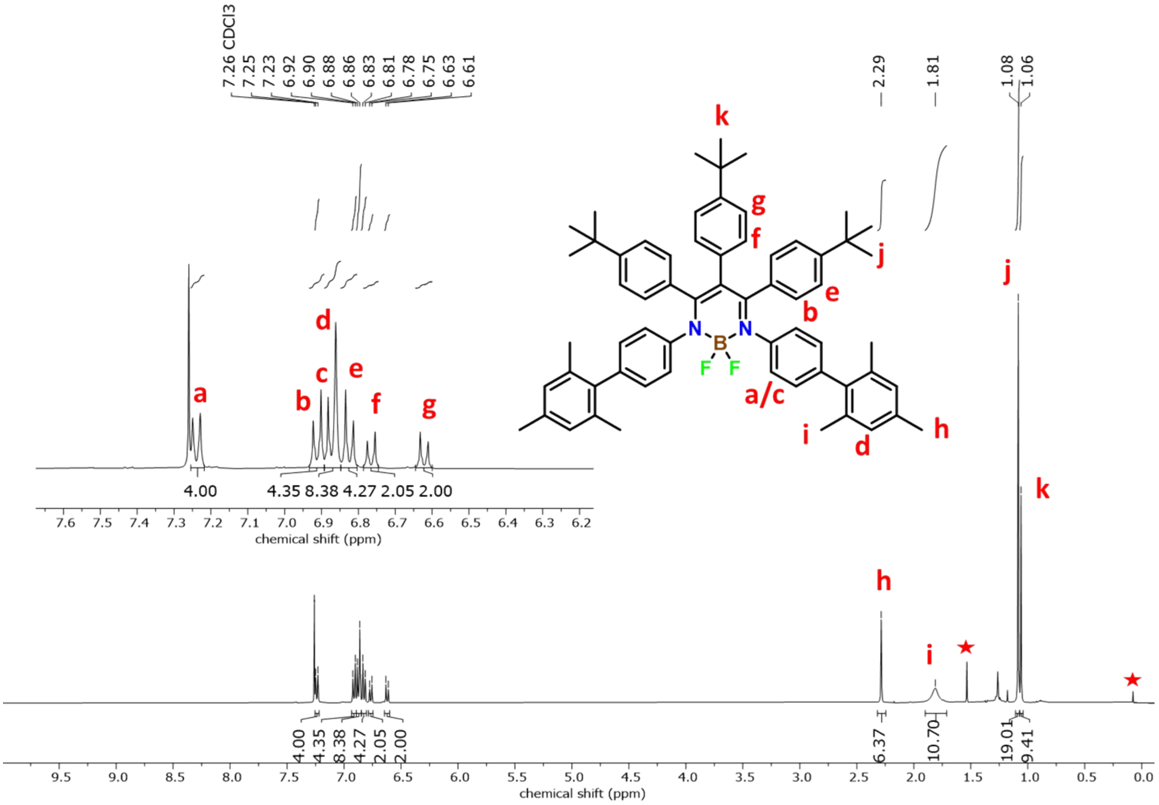

Figure S21: ^{1}H NMR spectrum of **^{TBP}BDI-BF$_2$** in CDCl$_3$, at 25 °C. The resonance marked with a star are attributed to residual water (1.56 ppm) and laboratory grease (0.08 ppm).

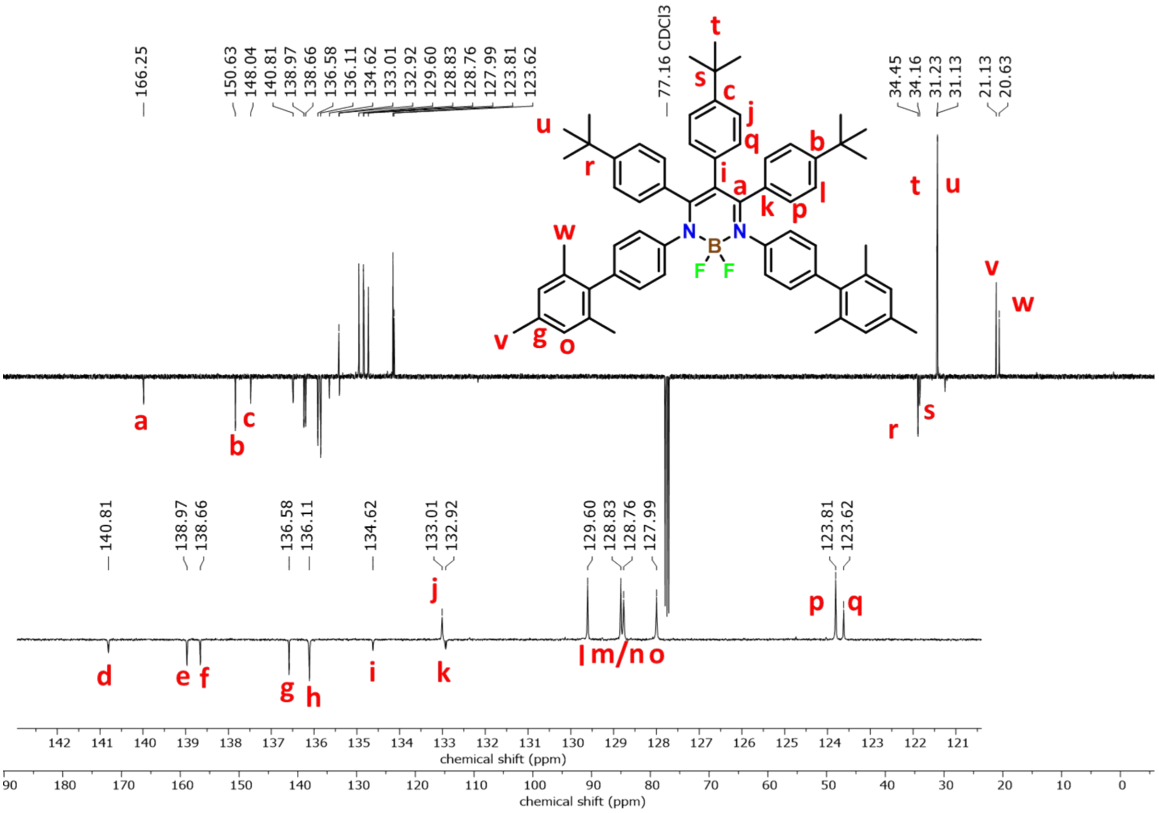

Figure S22: ^{13}C-APT NMR spectrum of **^{TBP}BDI-BF$_2$** in CDCl$_3$, at 25 °C.

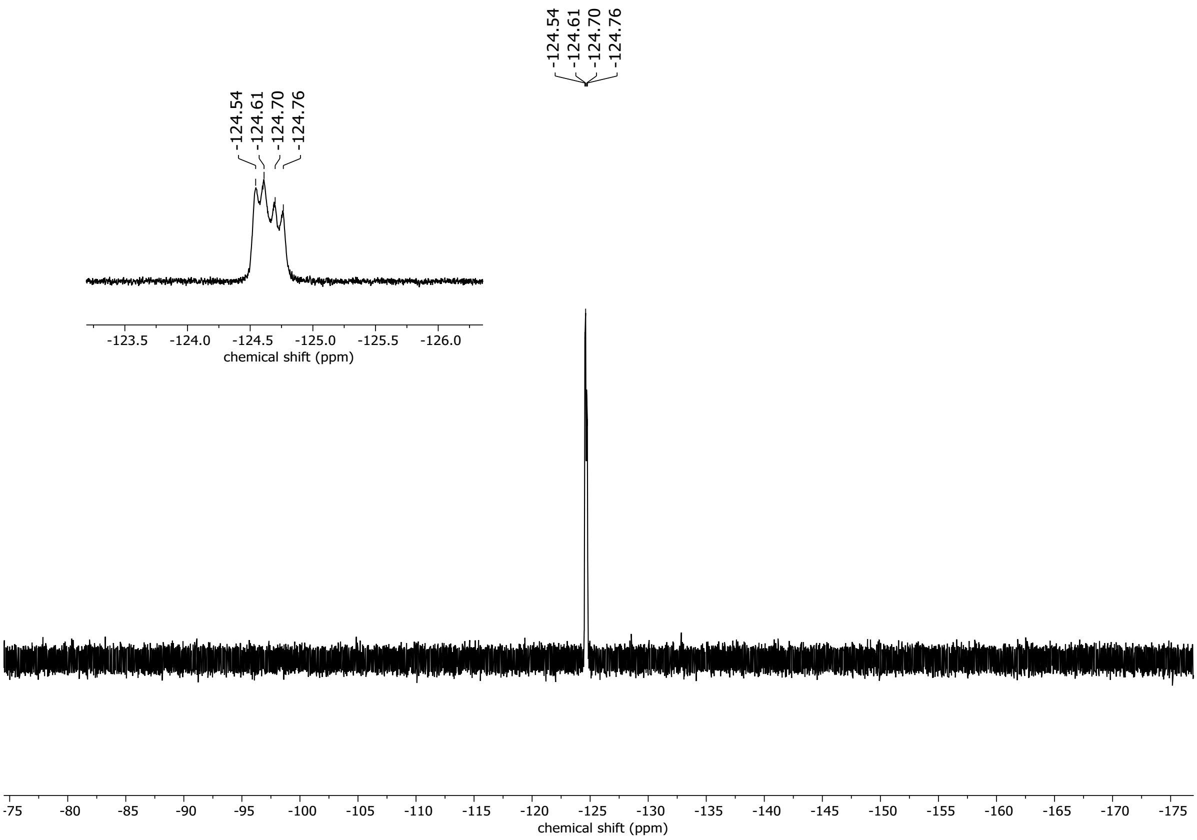

Figure S23: ^{19}F NMR spectrum of $^{\mathbf{TBP}}$**BDI-BF$_2$** in $CDCl_3$, at 25 °C.

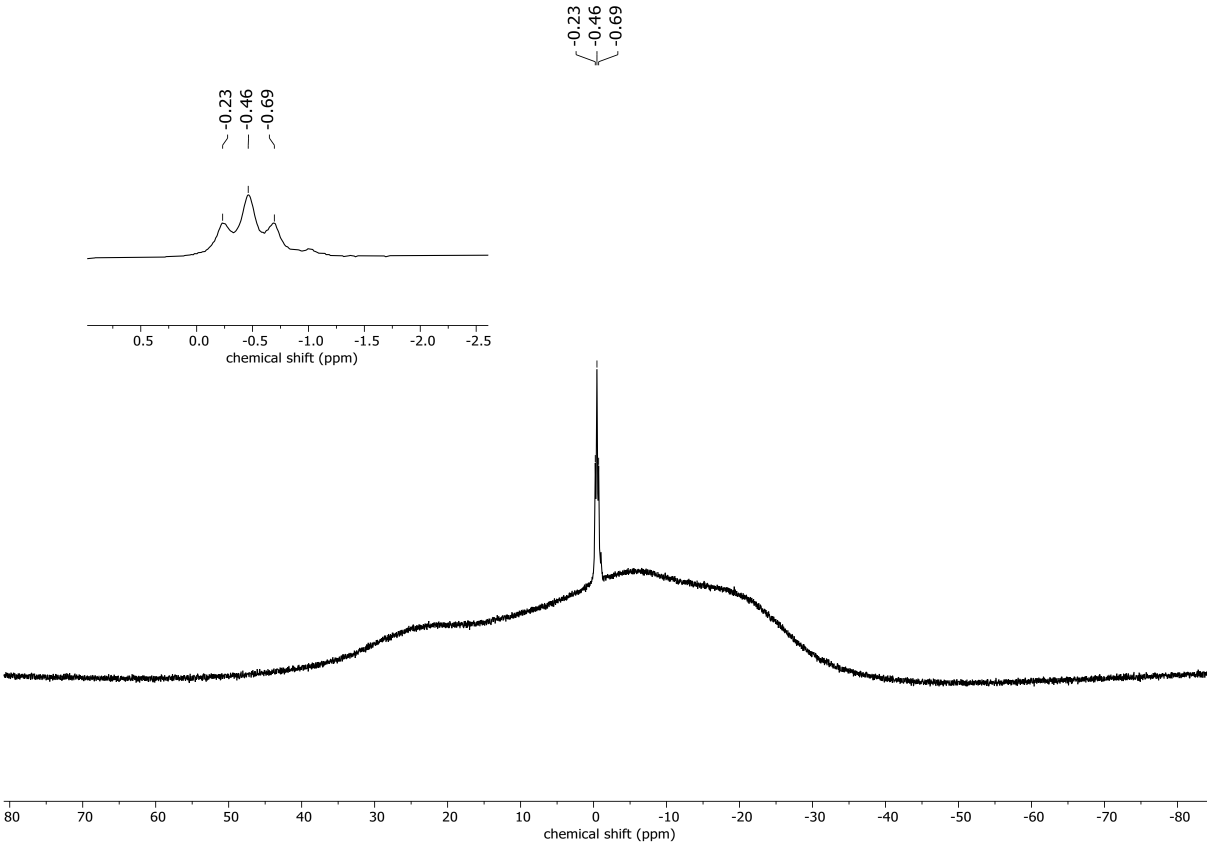

Figure S24: ^{11}B NMR spectrum of $^{\mathbf{TBP}}$**BDI-BF$_2$** in $CDCl_3$, at 25 °C.

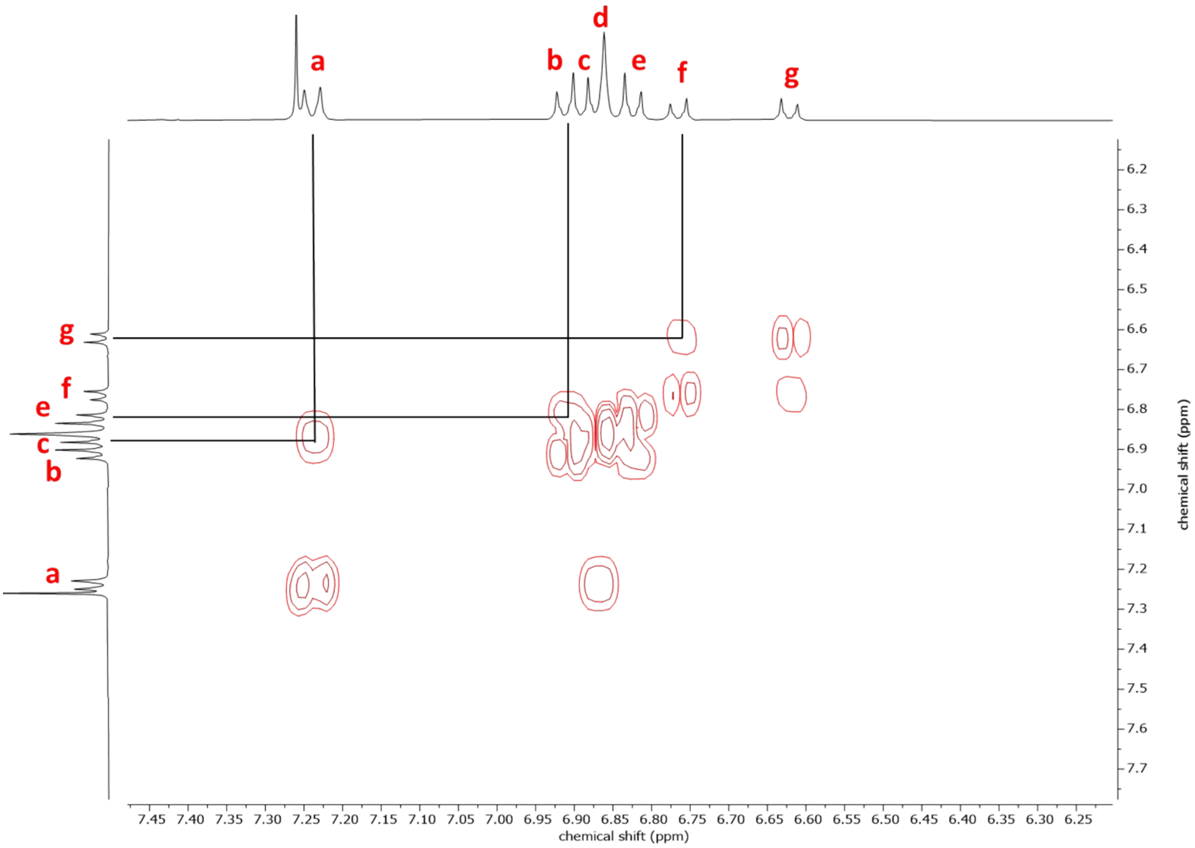

Figure S25: COSY NMR spectrum of ^{TBP}**BDI-BF$_2$** in $CDCl_3$, at 25 °C.

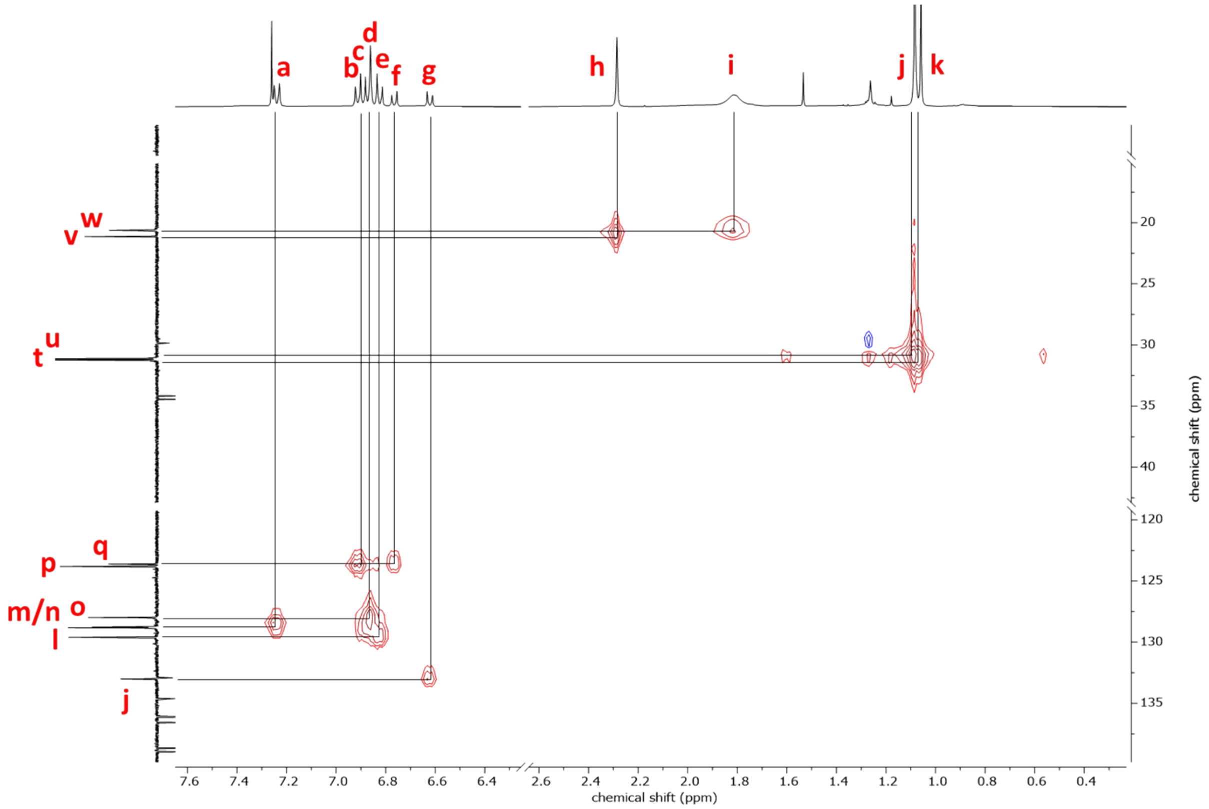

Figure S26: ^{1}H-^{13}C HSQC NMR spectrum of ^{TBP}**BDI-BF$_2$** in $CDCl_3$, at 25 °C.

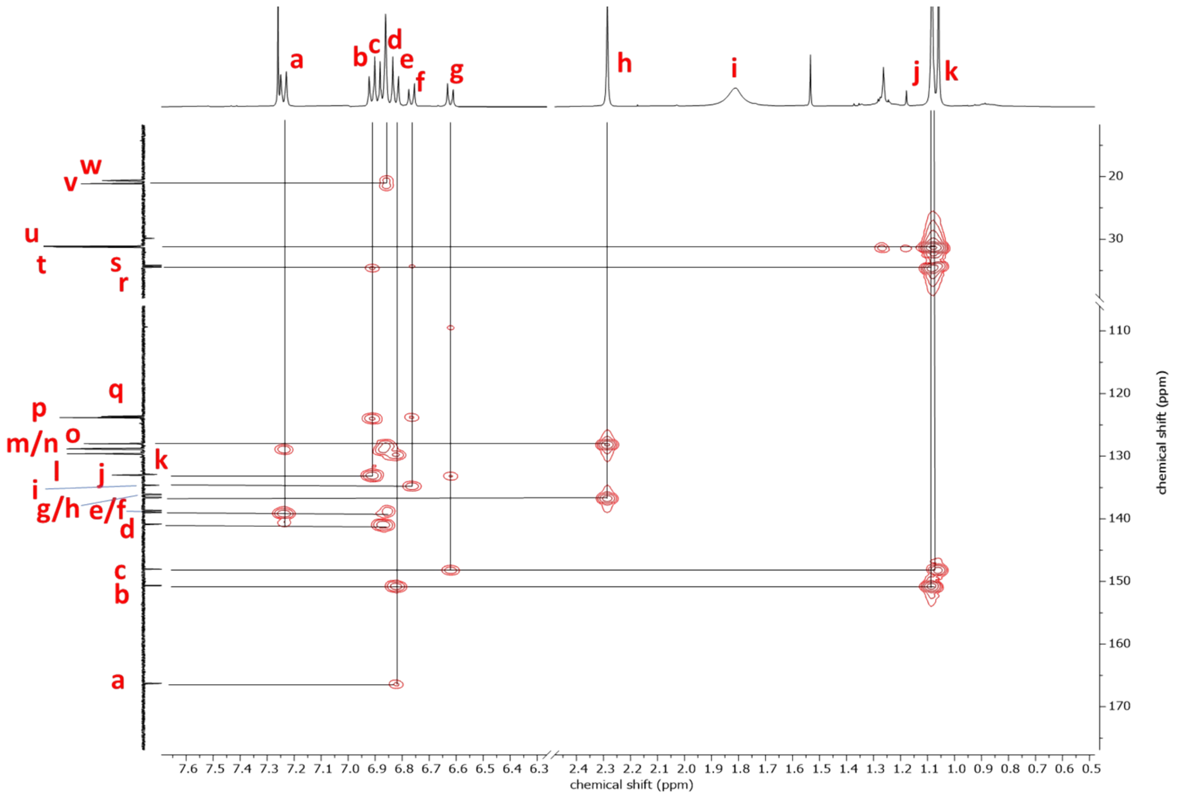

Figure S27: ^{1}H-^{13}C HMBC NMR spectrum of **^{TBP}BDI-BF$_2$** in $CDCl_3$, at 25 °C.

^{tBu}BT-BDI

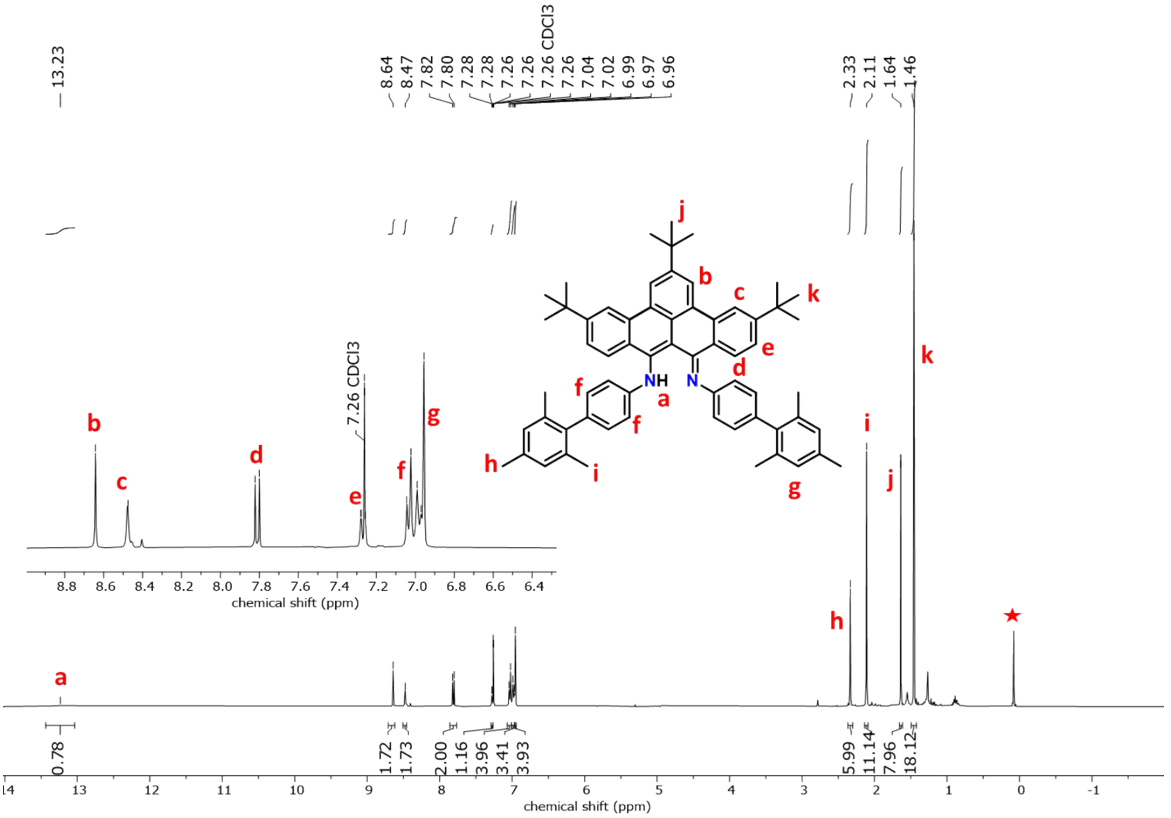

Figure S28: ^{1}H NMR spectrum of **^{tBu}BT-BDI** in $CDCl_3$, at 25 °C. The resonance marked with a star is attributed to residual laboratory grease and/or HMDSO (0.06 ppm).

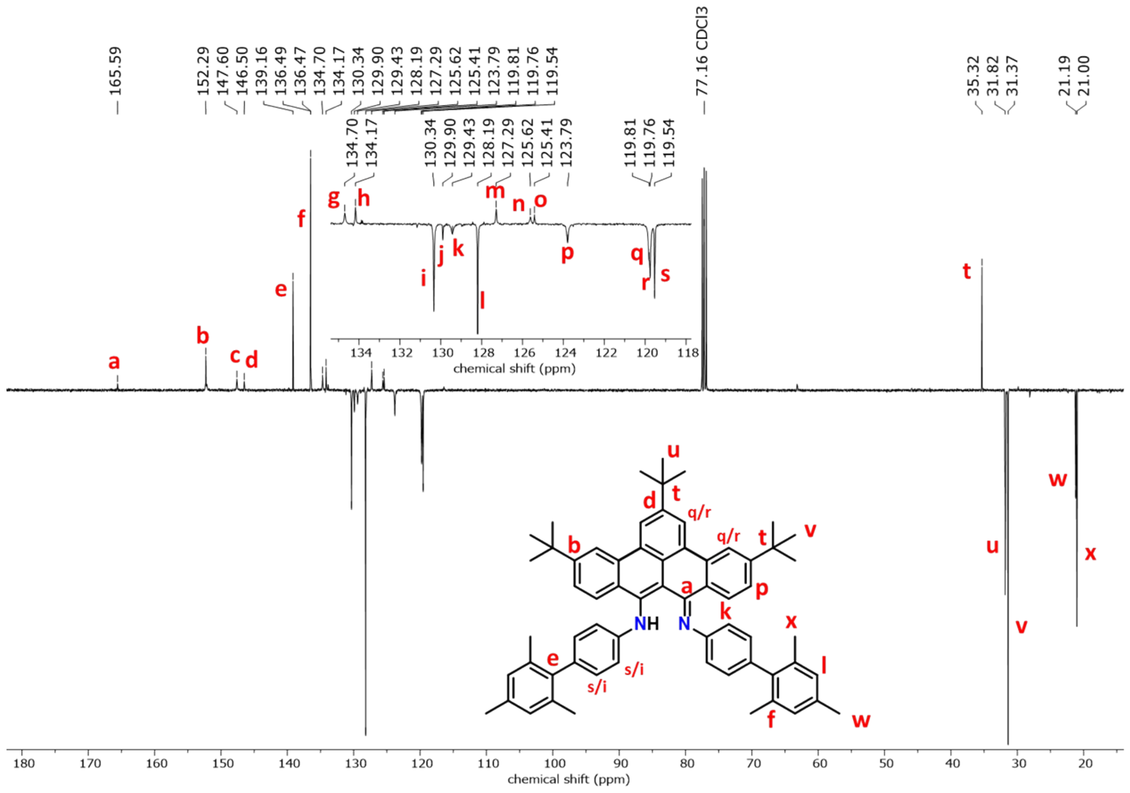

Figure S29: ^{13}C-APT NMR spectrum of ^{tBu}**BT-BDI** in $CDCl_3$, at 25 °C.

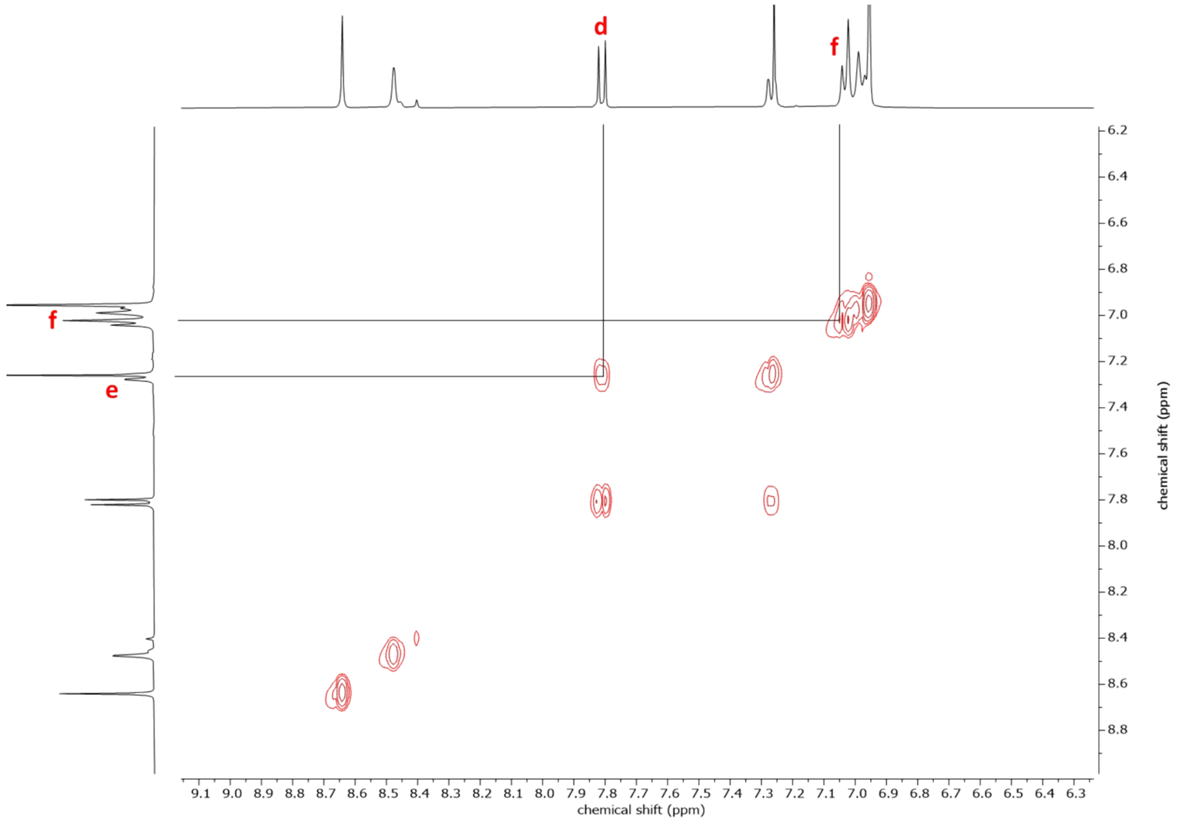

Figure S30: COSY NMR spectrum of ^{tBu}**BT-BDI** in $CDCl_3$, at 25 °C.

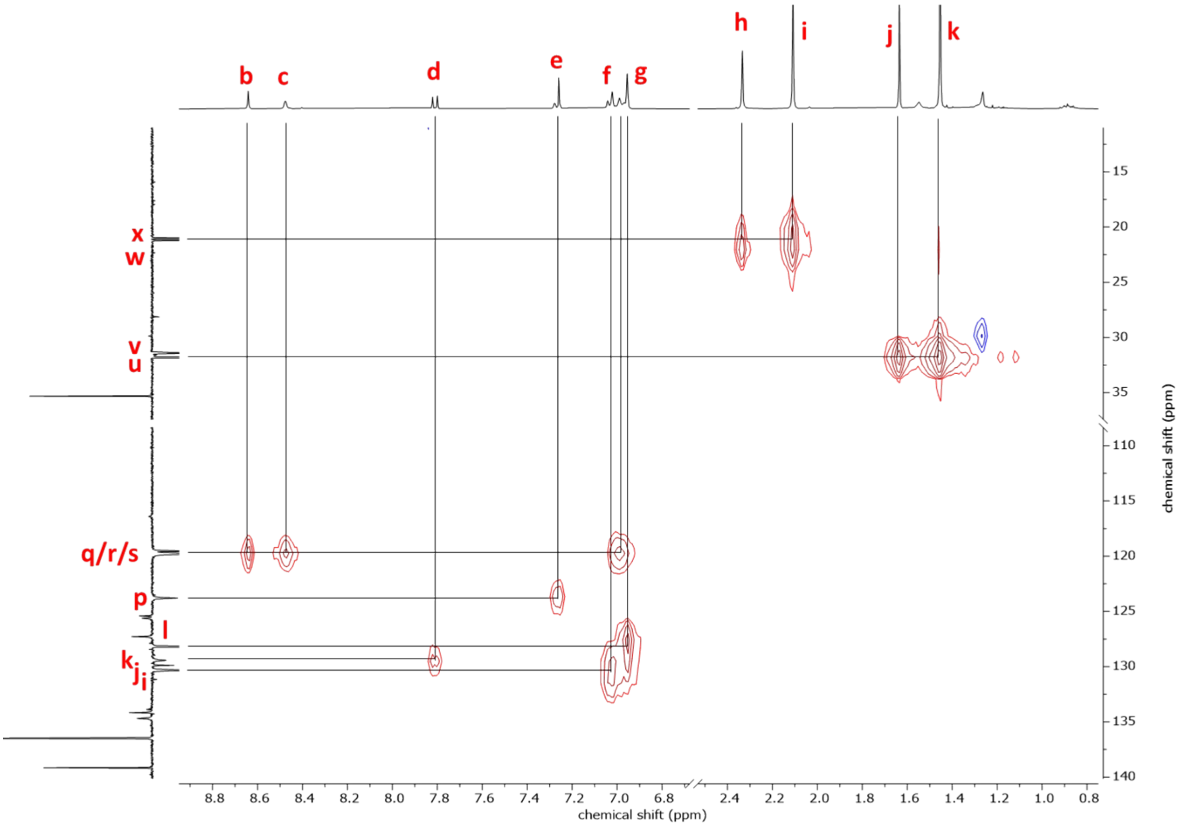

Figure S31: ^{1}H-^{13}C HSQC NMR spectrum of **^{tBu}BT-BDI** in $CDCl_3$, at 25 °C.

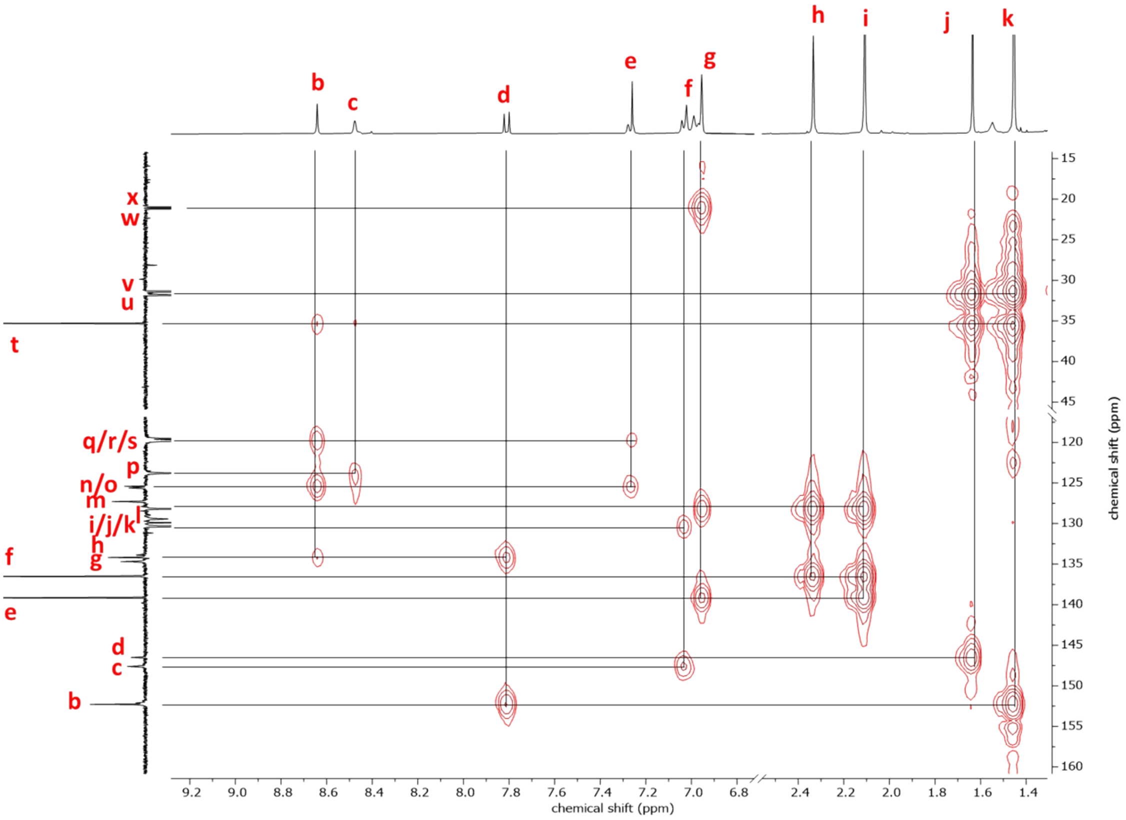

Figure S32: ^{1}H-^{13}C HMBC NMR spectrum of **^{tBu}BT-BDI** in $CDCl_3$, at 25 °C.

^{Cl}BT-BDI

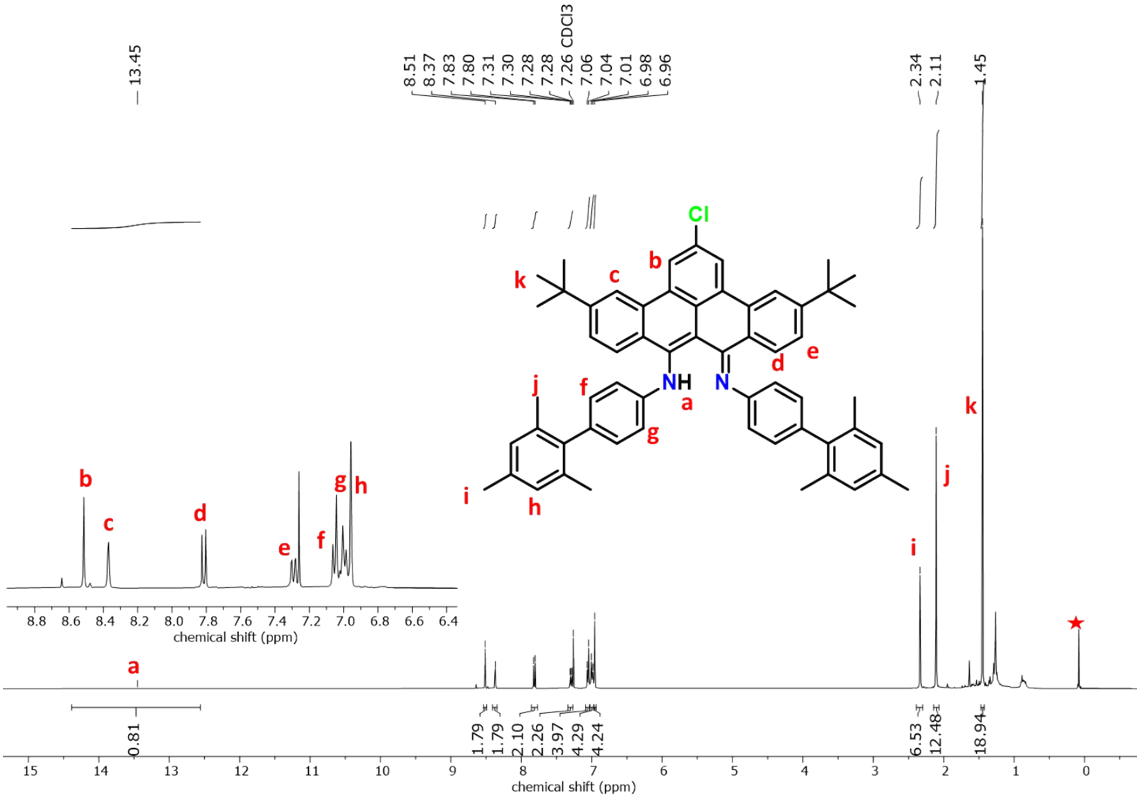

Figure S33: ^{1}H NMR spectrum of **^{Cl}BT-BDI** in $CDCl_3$, at 25 °C. The resonance marked with a star is attributed to residual HMDSO.

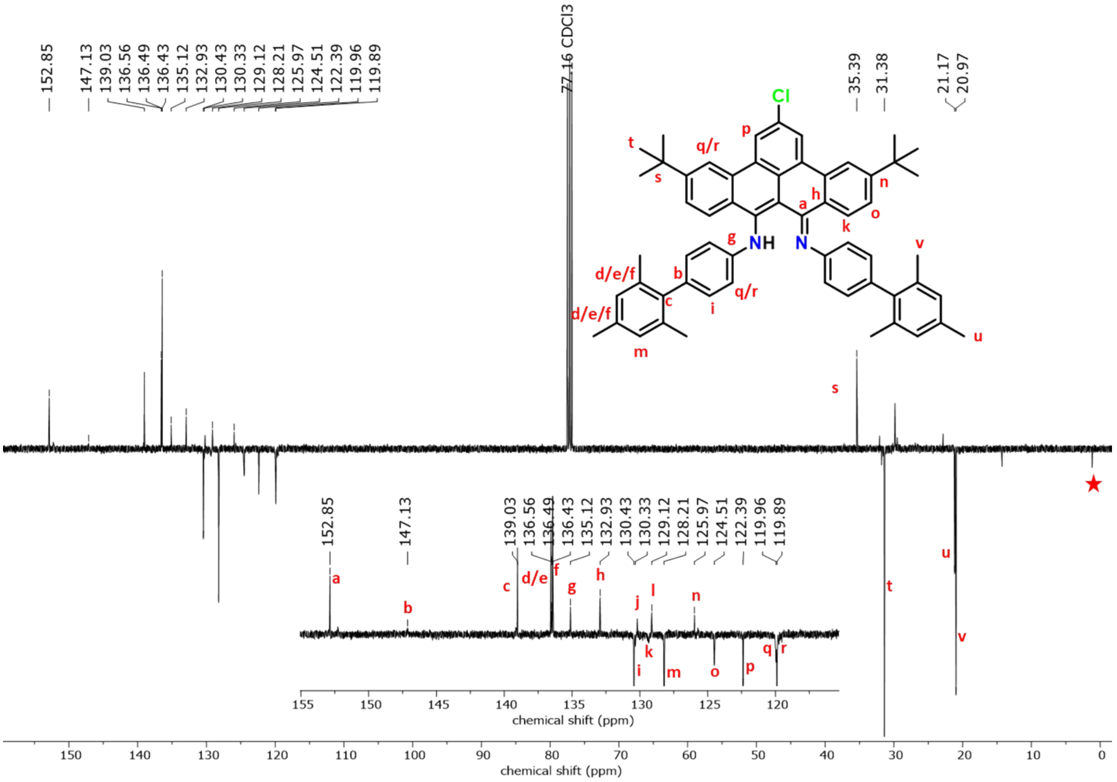

Figure S34: ^{13}C NMR spectrum of **^{Cl}BT-BDI** in $CDCl_3$, at 25 °C. The resonance marked with a star is attributed to laboratory grease and/or HMDSO.

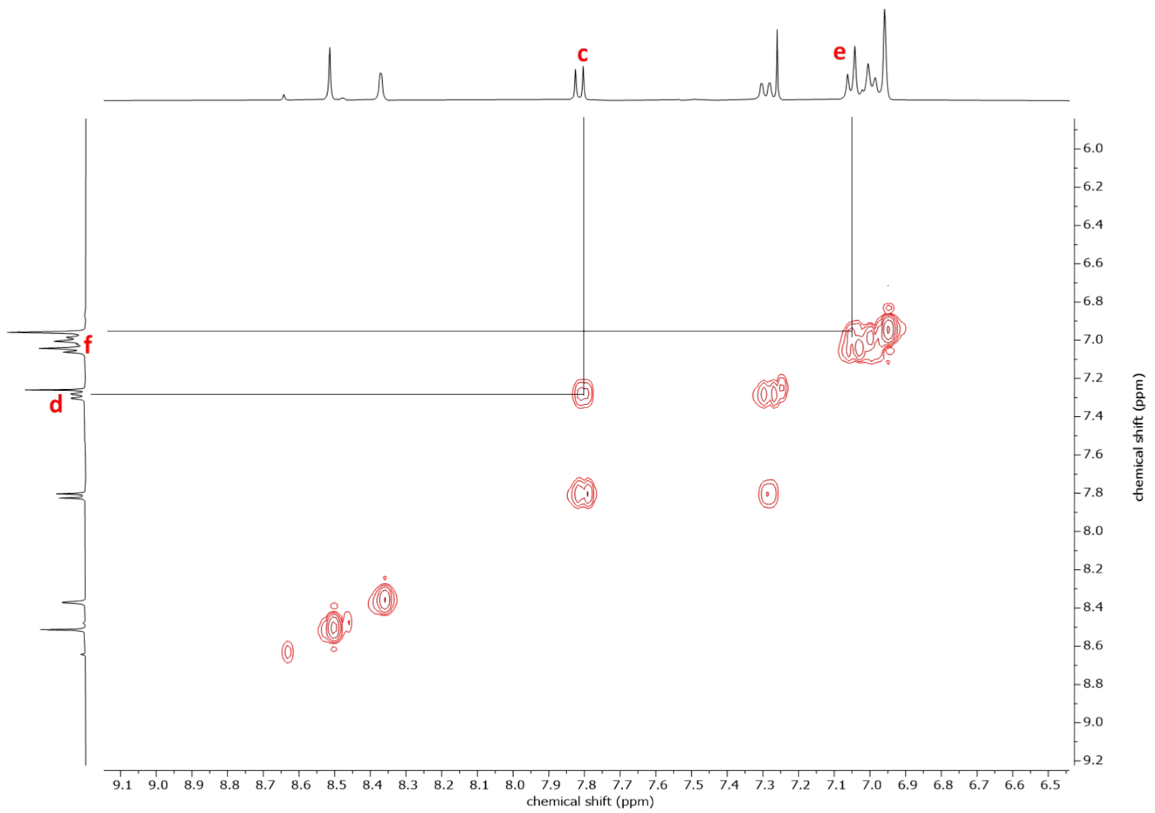

Figure S35: COSY NMR spectrum of **^{Cl}BT-BDI** in $CDCl_3$, at 25 °C.

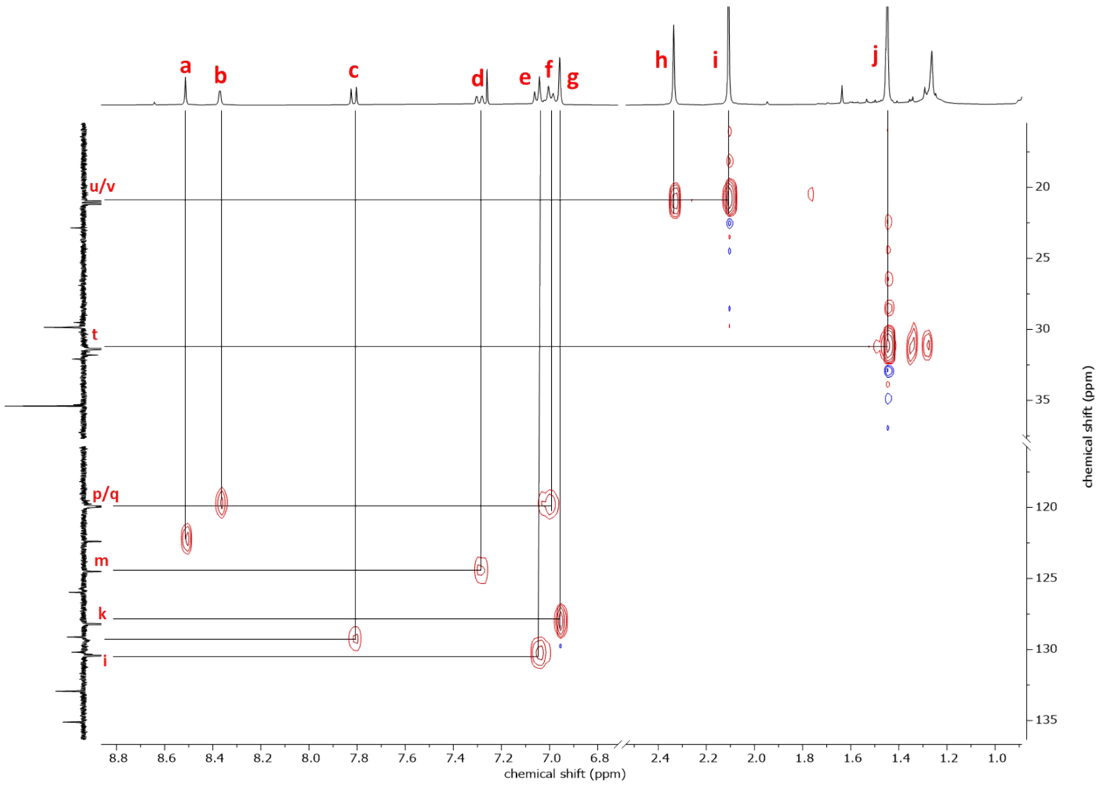

Figure S36: ^{1}H-^{13}C HSQCAD NMR spectrum of **^{Cl}BT-BDI** in $CDCl_3$, at 25 °C.

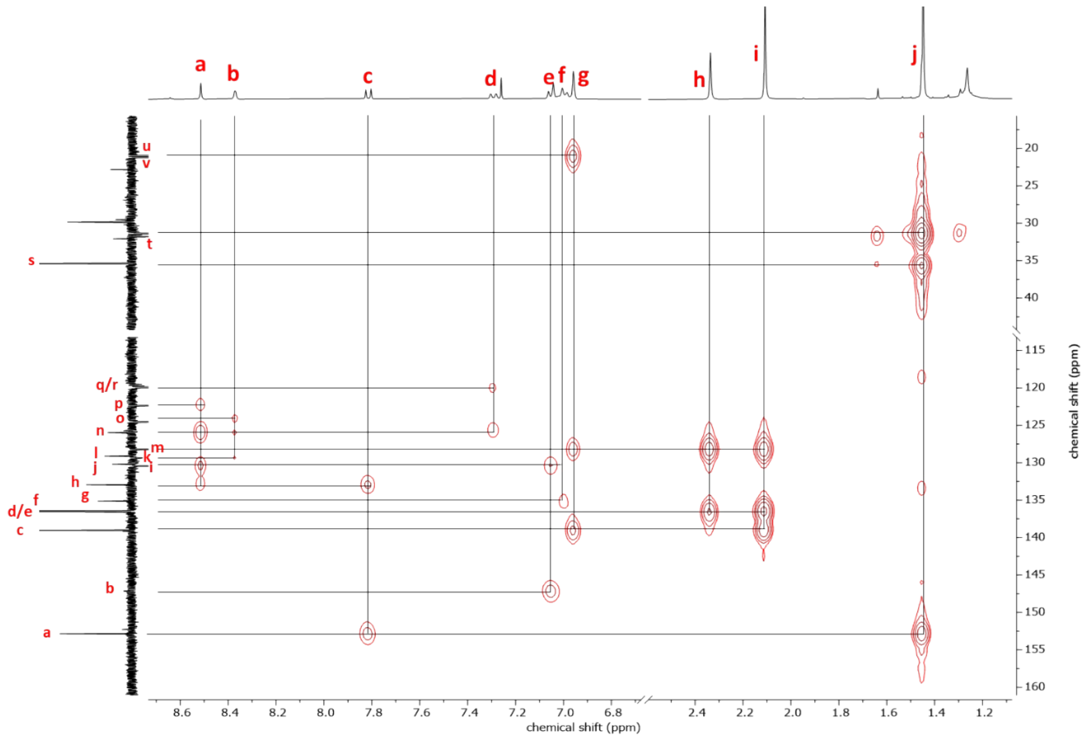

Figure S37: ^{1}H-^{13}C HMBC NMR spectrum of ^{Cl}**BT-BDI** in $CDCl_3$, at 25 °C.

BT-BDI-BF_2

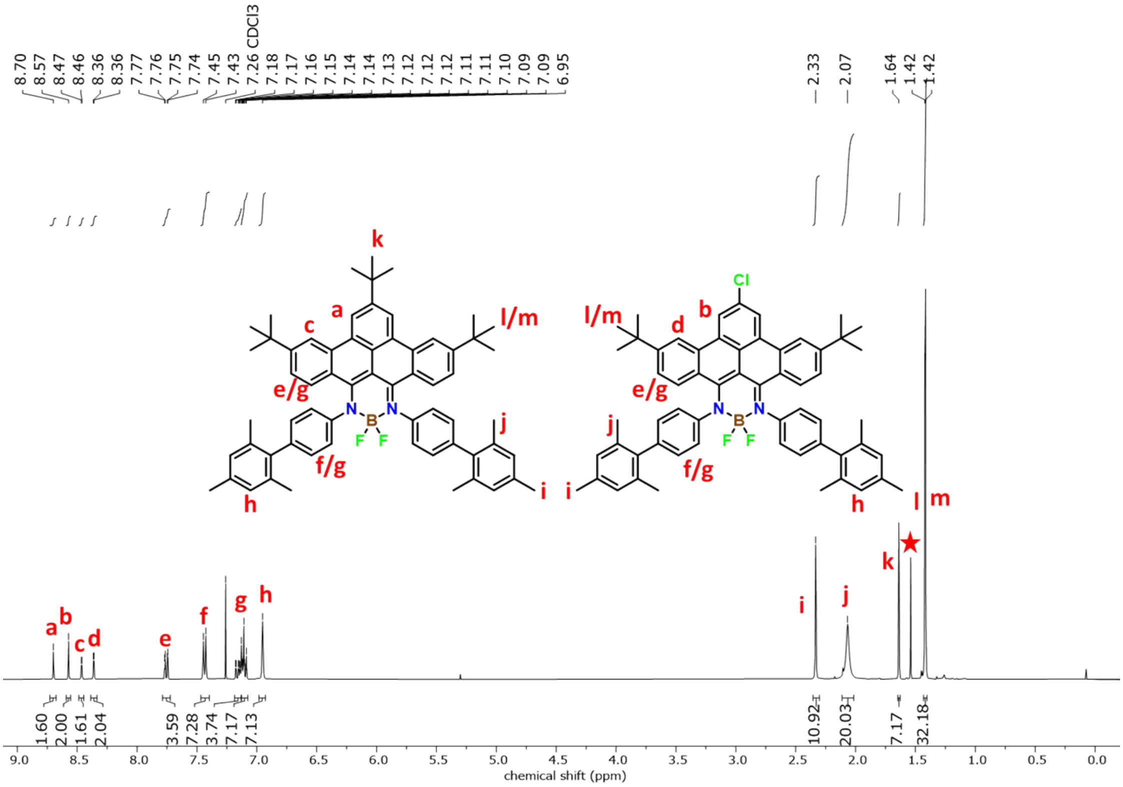

Figure S38: ^{1}H NMR spectrum of **BT-BDI-BF_2** in $CDCl_3$, at 25 °C. The resonance marked with a star is attributed to residual water in the solvent.

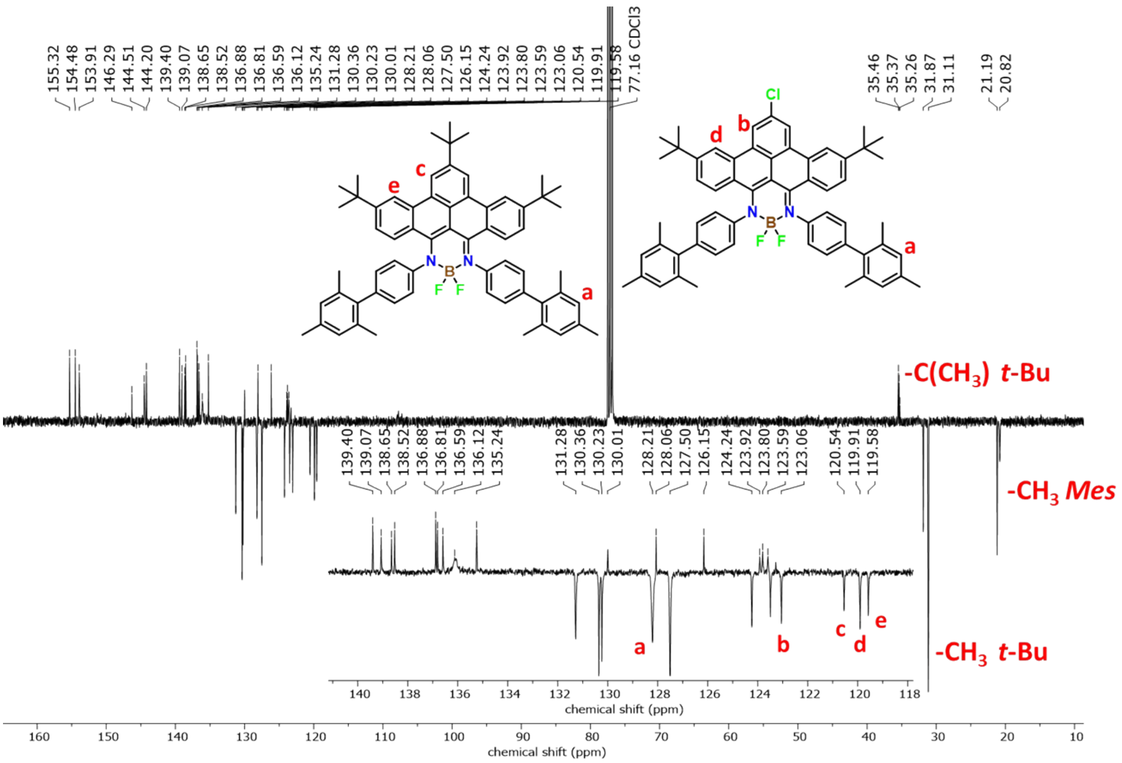

Figure S39: ^{13}C-APT NMR spectrum of **BT-BDI-BF$_2$** in $CDCl_3$, at 25 °C.

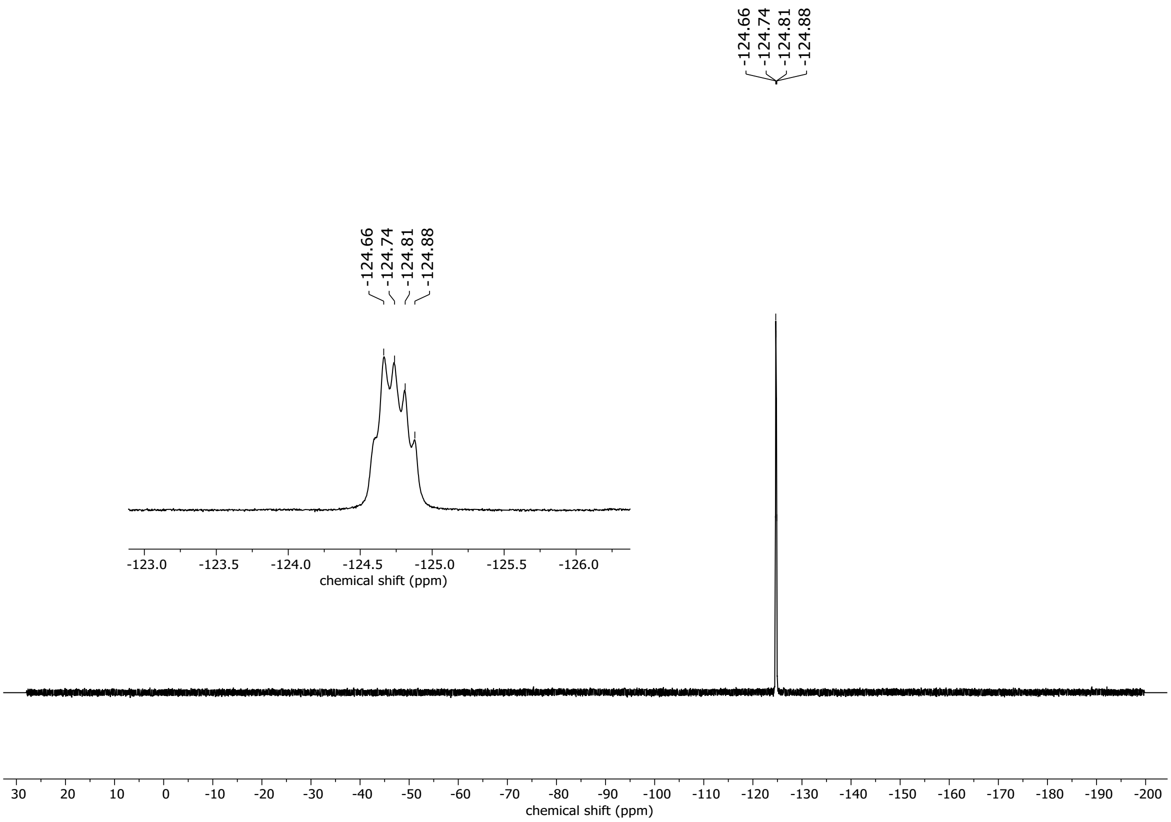

Figure S40: ^{19}F NMR spectrum of **BT-BDI-BF$_2$** in $CDCl_3$, at 25 °C.

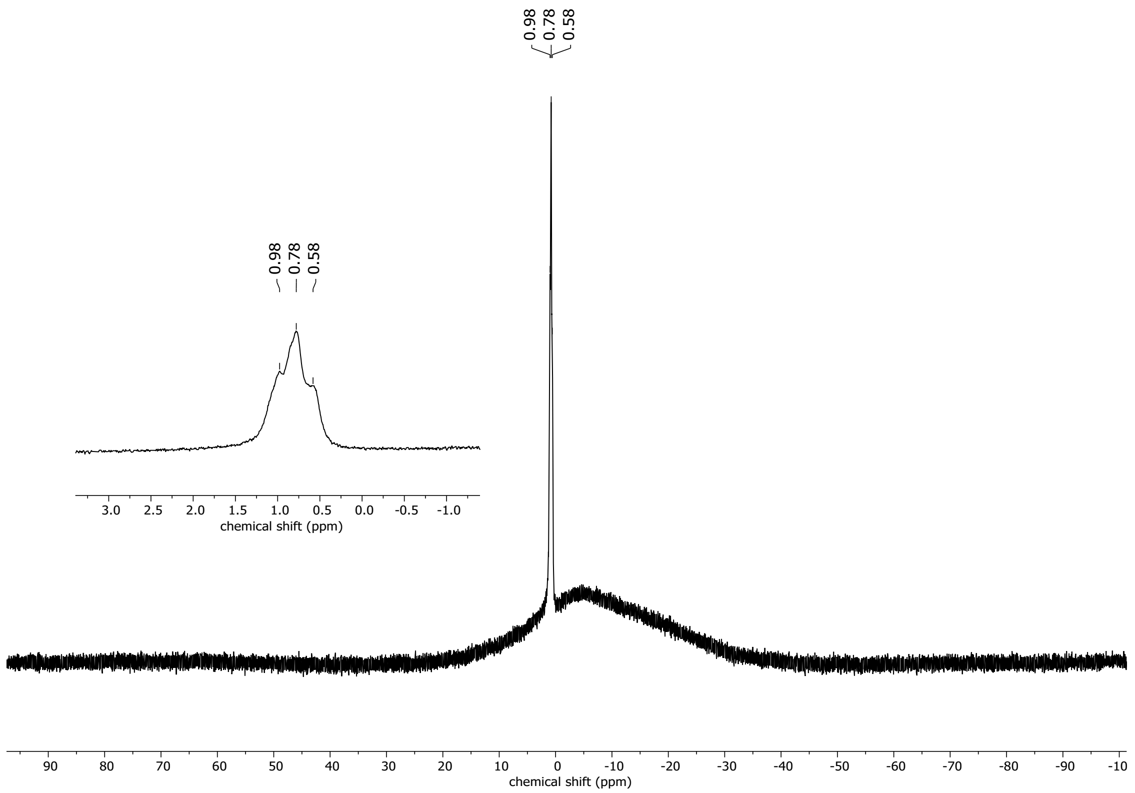

Figure S41: The ^{11}B NMR spectrum of **BT-BDI-BF$_2$** in CDCl$_3$, at 25 °C.

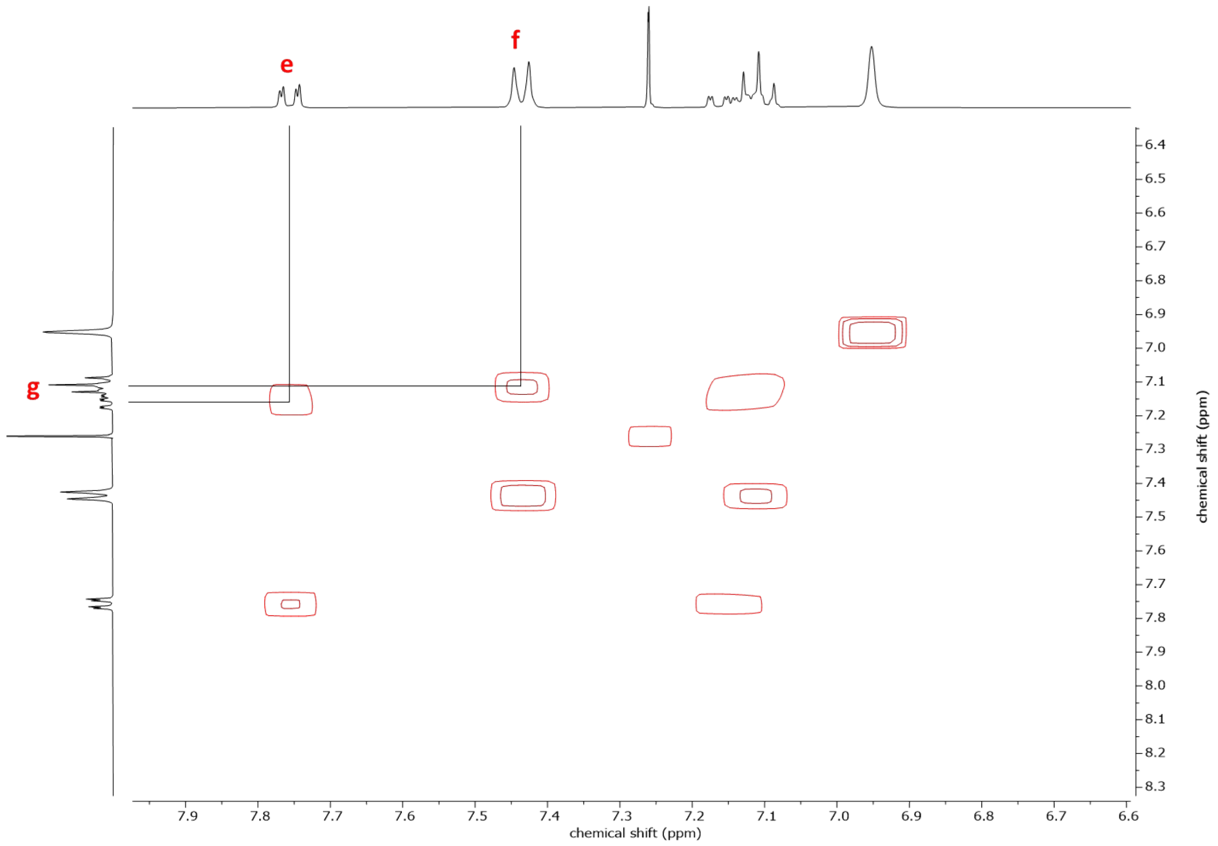

Figure S42: gCOSY NMR spectrum of **BT-BDI-BF$_2$** in CDCl$_3$, at 25 °C.

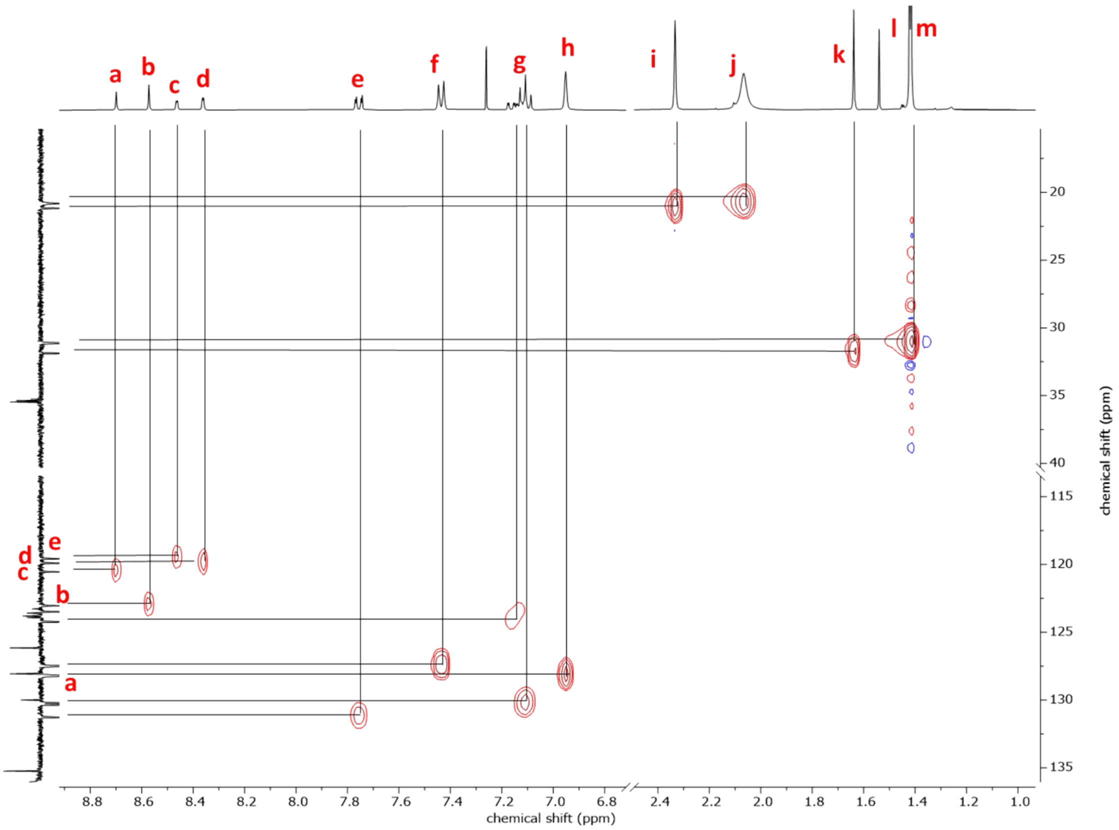

Figure S43: ^{1}H-^{13}C HSQCAD NMR spectrum of **BT-BDI-BF₂** in $CDCl_3$, at 25 °C.

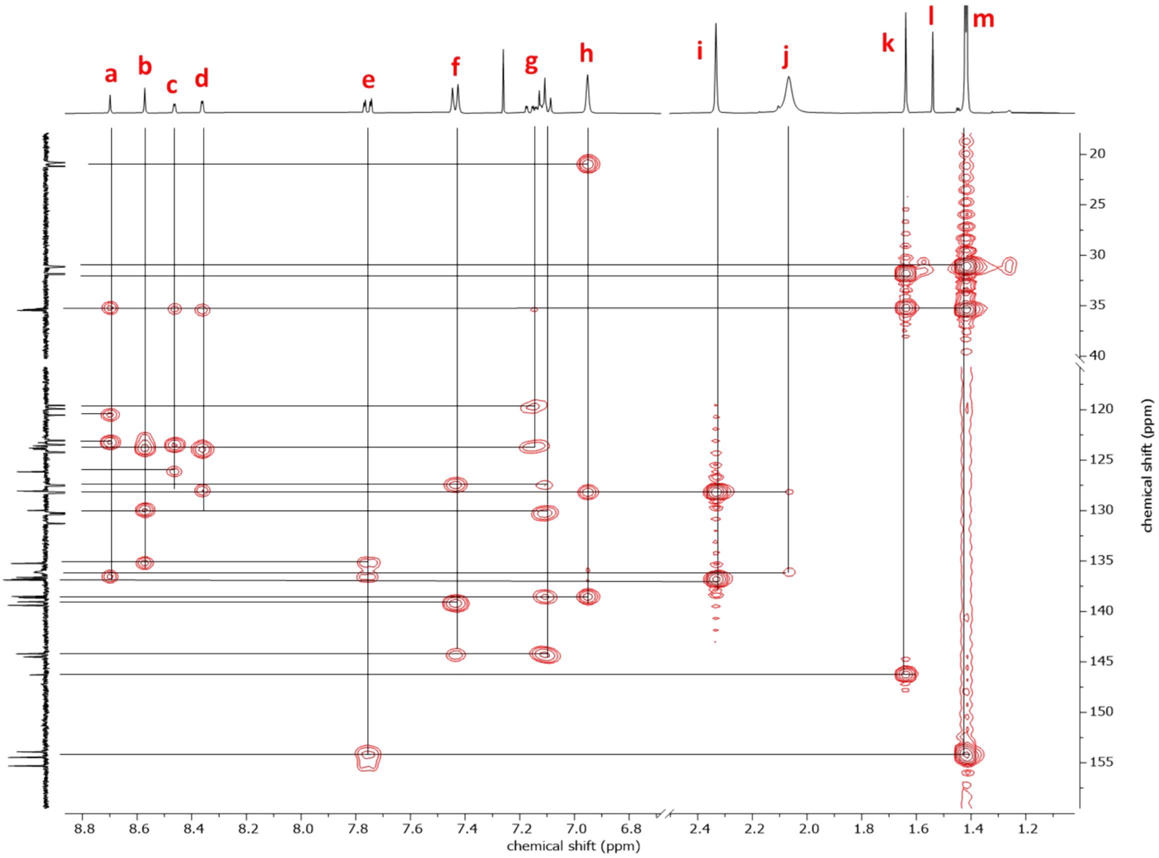

Figure S44: ^{1}H-^{13}C gHMBCAD NMR spectrum of **BT-BDI-BF_2** in $CDCl_3$, at 25 °C.

^{tBu}BT-BDI-ZnEt

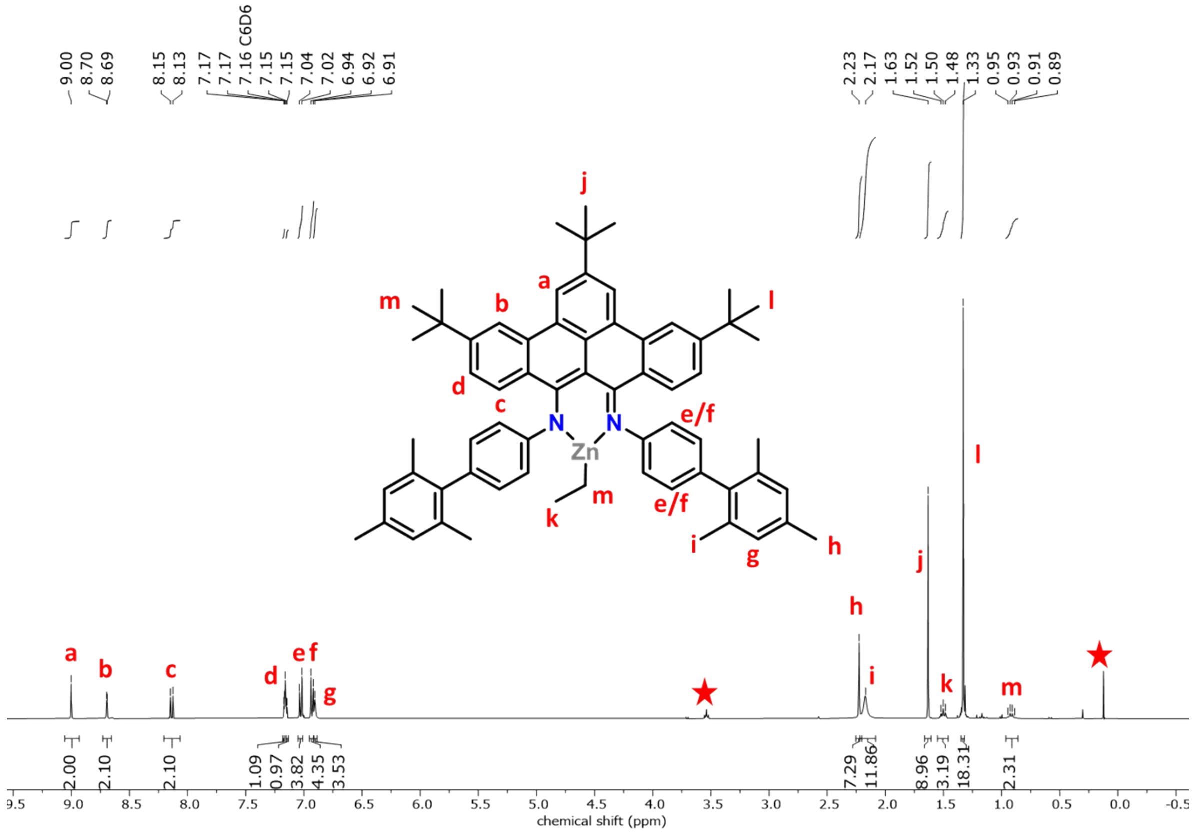

Figure S45: ^{1}H NMR spectrum of **^{tBu}BT-BDI-ZnEt** in C_6D_6, at 25 °C. Resonances marked with a star are attributed to residual THF (3.54 ppm) and HMDSO (0.12 ppm).

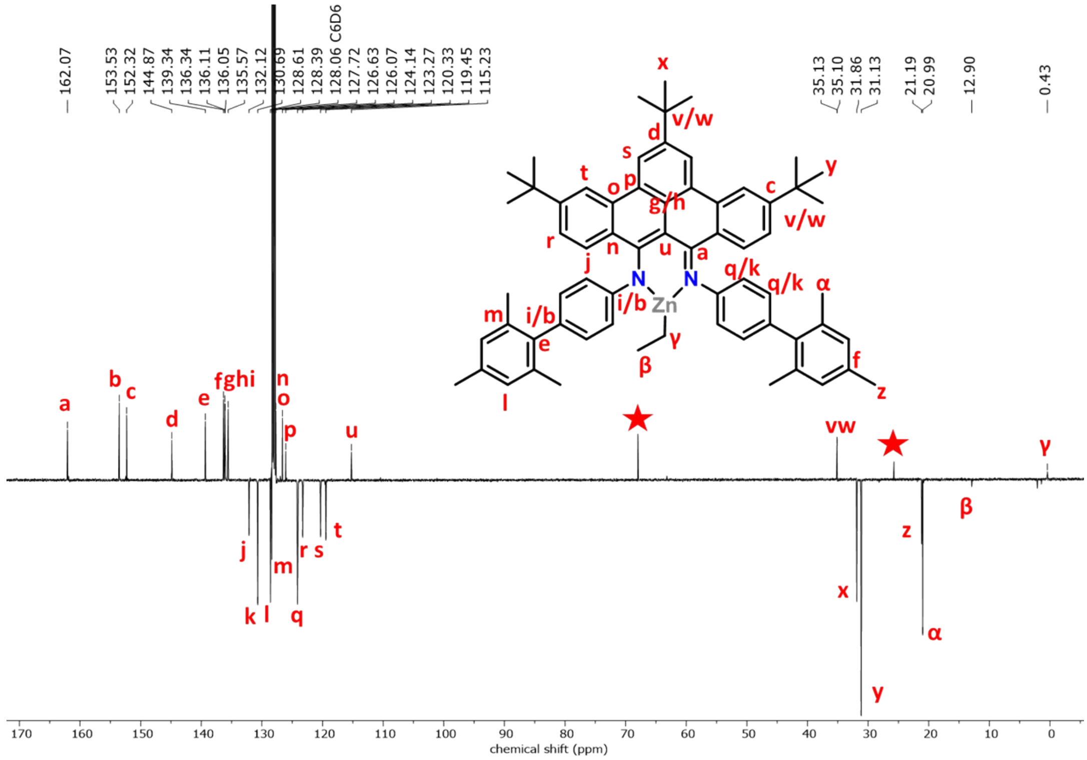

Figure S46: ^{13}C-APT NMR spectrum of **^{tBu}BT-BDI-ZnEt** in C_6D_6, at 25 °C. The resonances marked with a star are attributed to residual THF.

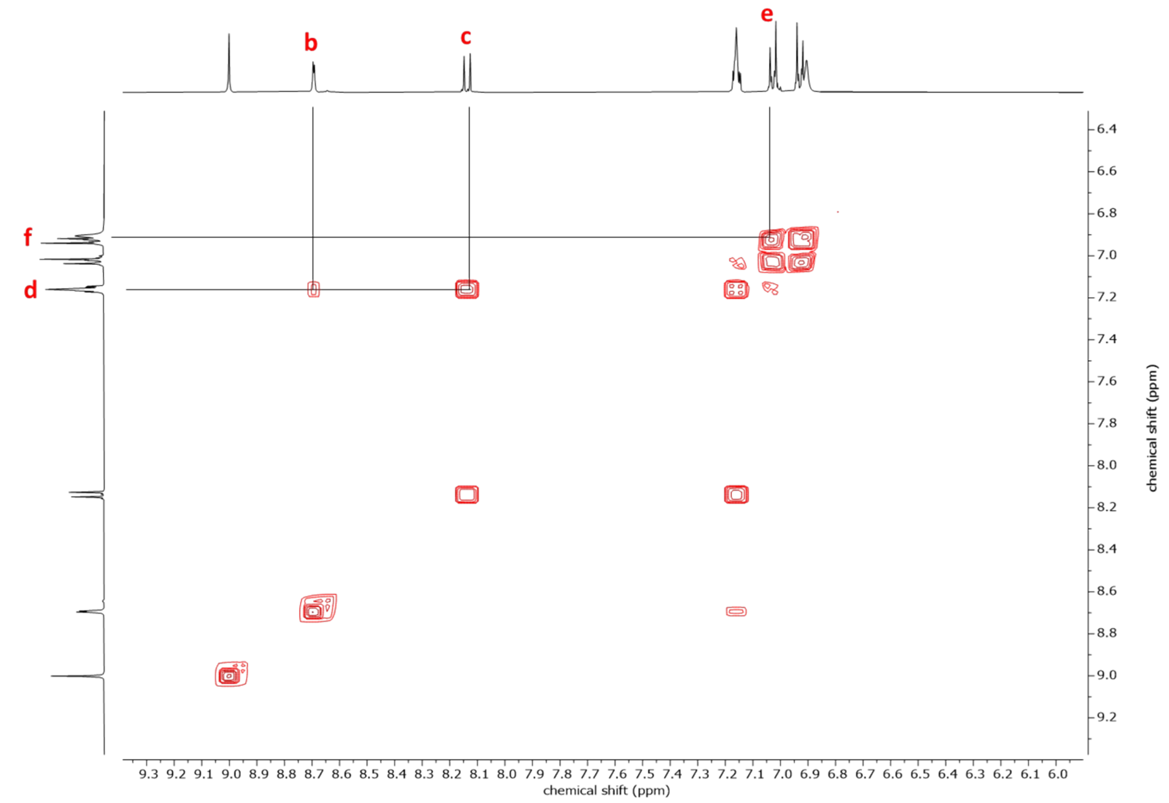

Figure S47: COSY NMR spectrum of **^{tBu}BT-BDI-ZnEt** in C_6D_6, at 25 °C.

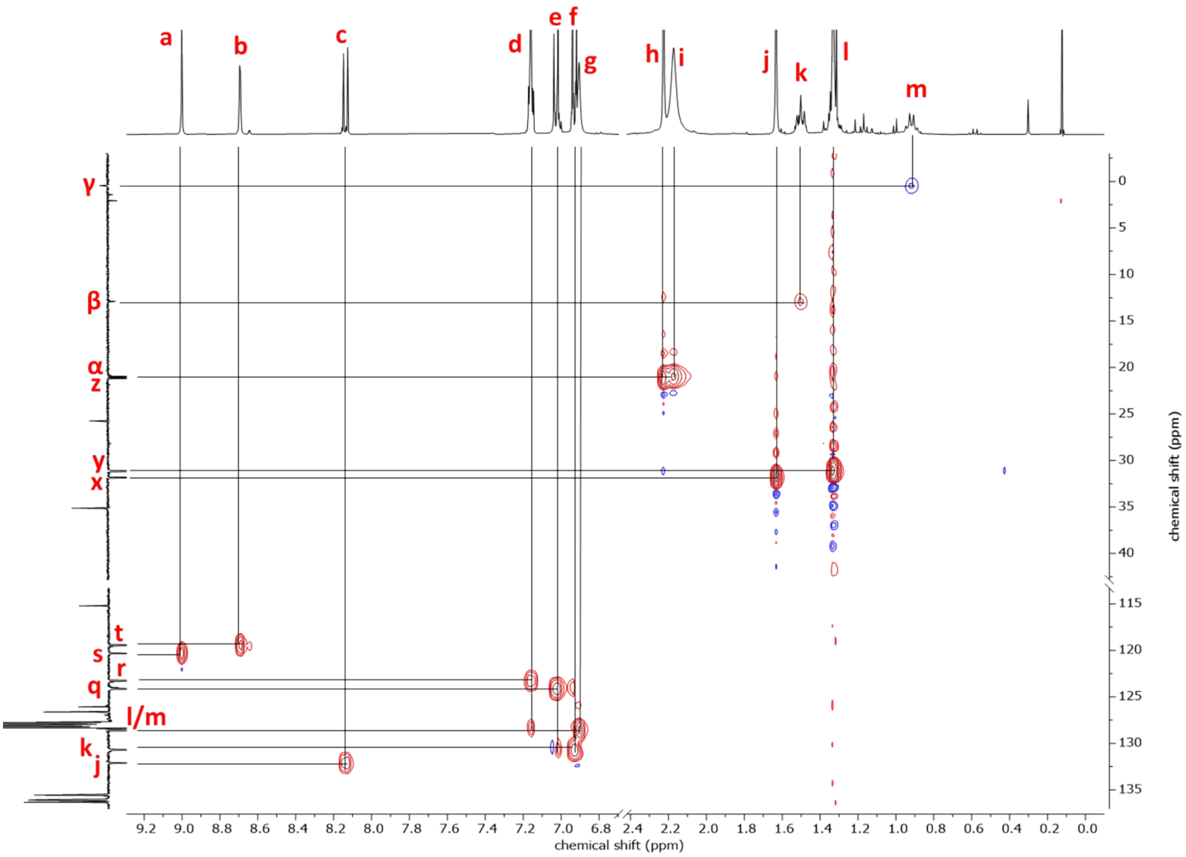

Figure S48: ^{1}H-^{13}C HSQCAD spectrum of **^{tBu}BT-BDI-ZnEt** in C_6D_6, at 25 °C.

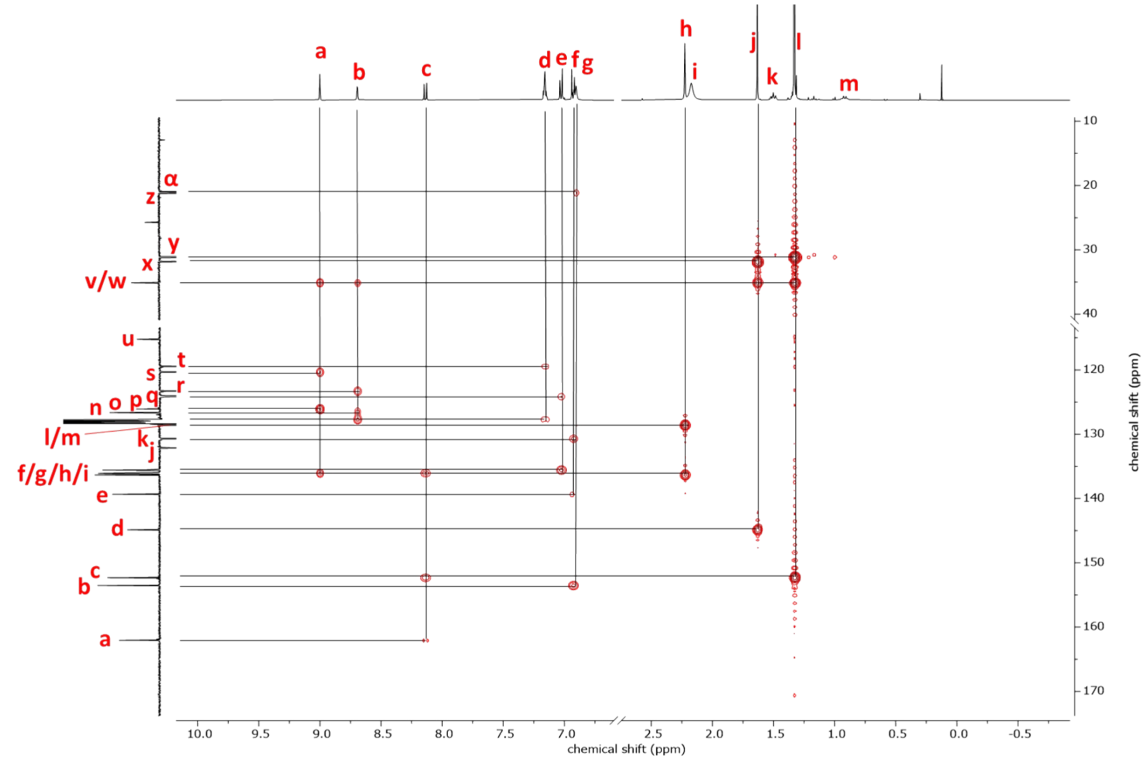

Figure S49: ^{1}H-^{13}C gHMBCAD spectrum of **^{tBu}BT-BDI-ZnEt** in C_6D_6, at 25 °C.

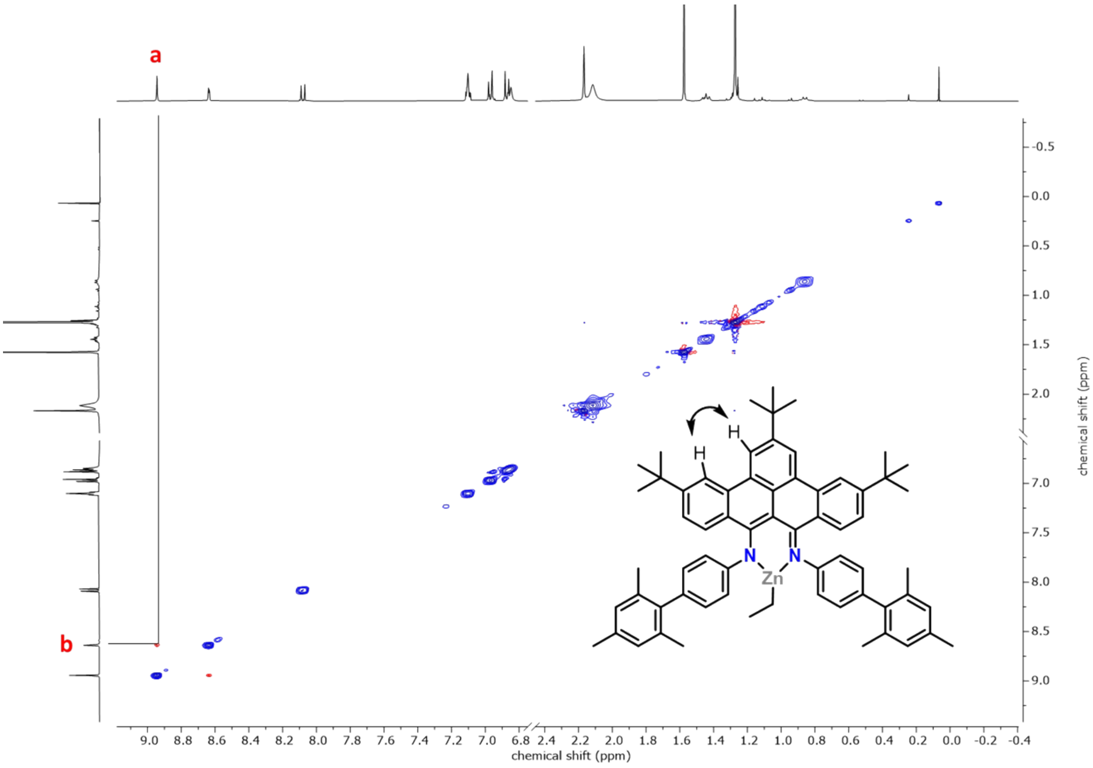

Figure S50: NOESY NMR spectrum of **^{tBu}BT-BDI-ZnEt** in C_6D_6, at 25 °C.

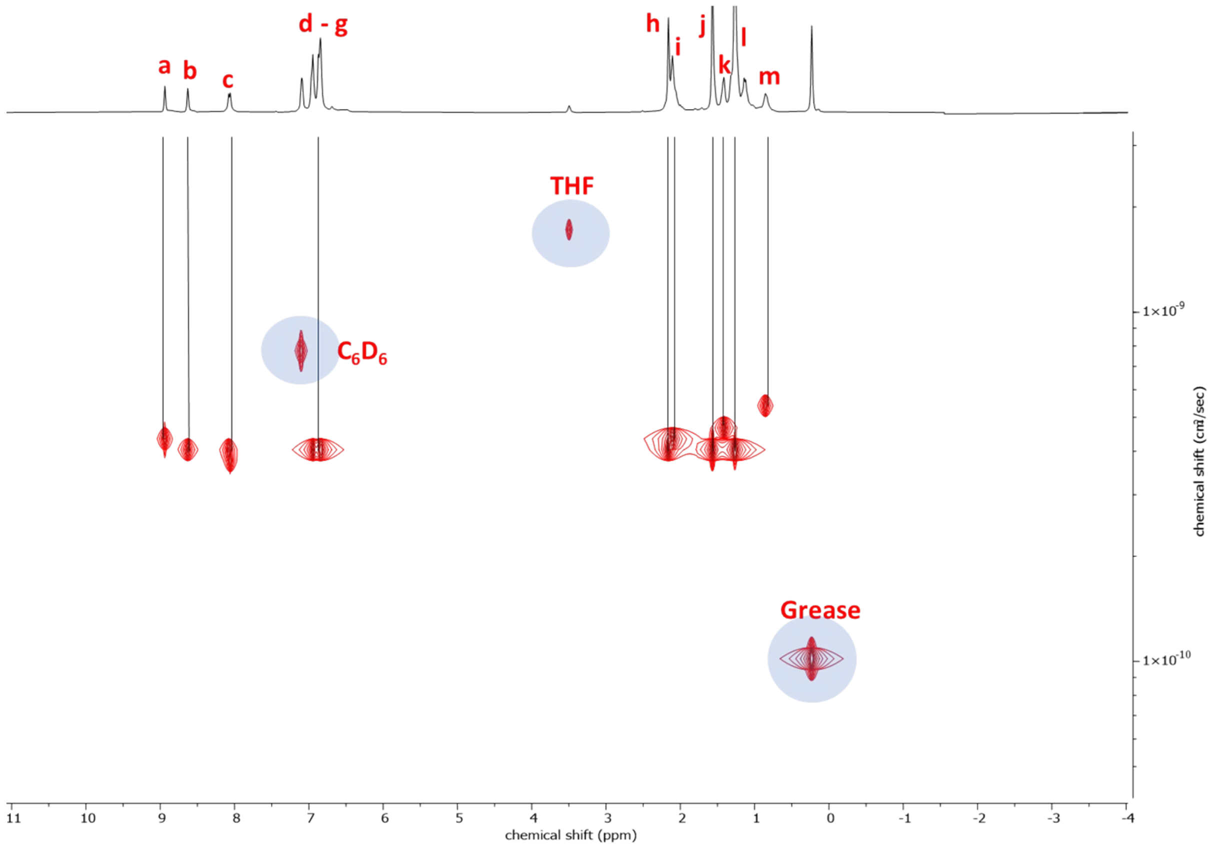

Figure S51: Convection corrected DOSY NMR spectrum of **^{tBu}BT-BDI-ZnEt** in C_6D_6, at 25 °C. Note that the signals from the ethyl group (k and m) show a diffusion constant in the same range as the rest of the ligand, suggesting they are from a similarly diffusion species.

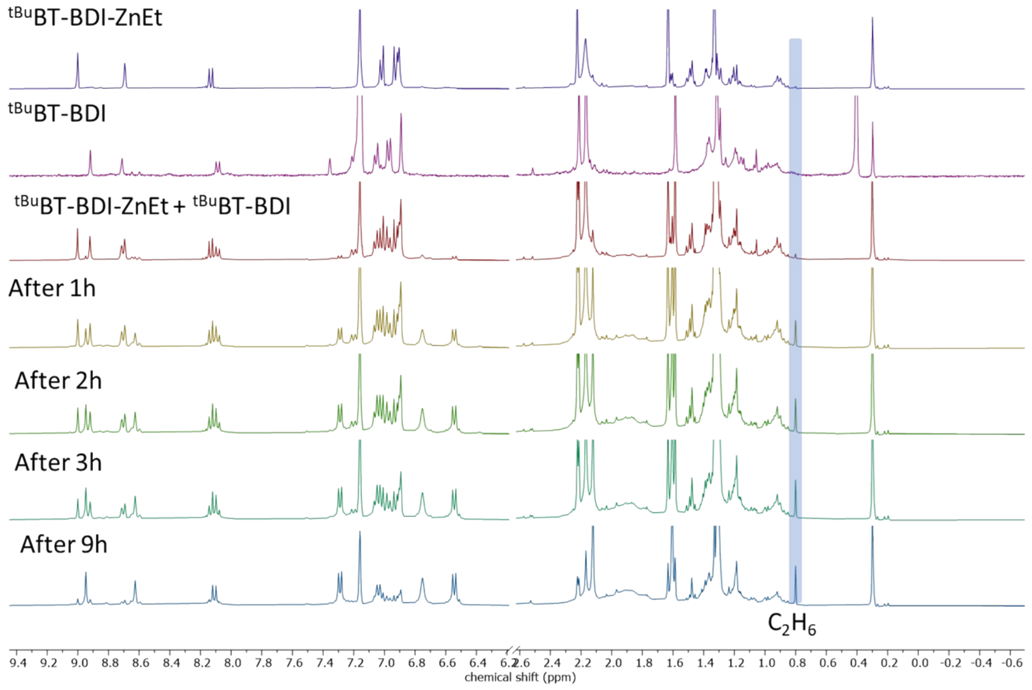

Figure S52: Stacked ^{1}H NMR spectra of **^{tBu}BT-BDI**, **^{tBu}BT-BDI-ZnEt** and a mixture of both directly after mixing and after several hours at room temperature in C_6D_6 at 25 °C. Note that in this timeframe, both compounds react to form a new, unidentified species, and ethane evolution is observed.

^{TBP}Indole·HMeSO$_3$

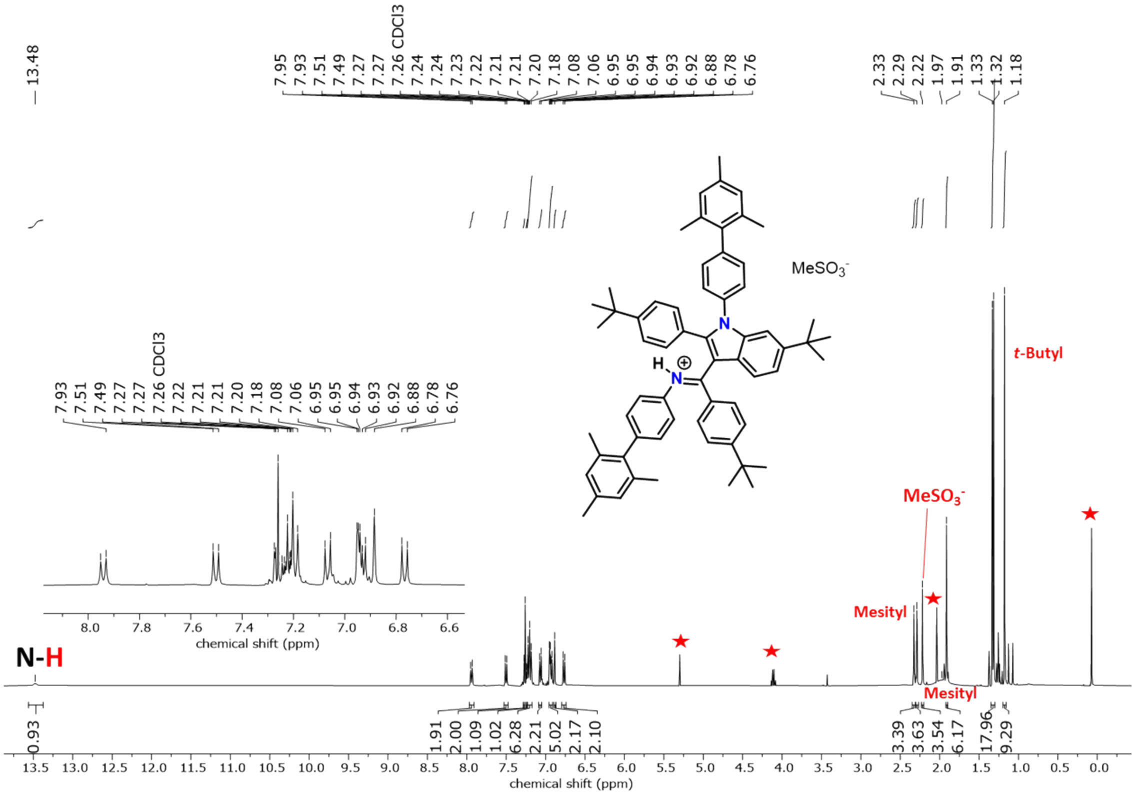

Figure S53: ^{1}H NMR spectrum of **^{TBP}Indole·HMeSO$_3$** in CDCl$_3$, at 25 °C. The resonances marked with a star are attributed to DCM (5.30 ppm), ethyl acetate (4.11, 2.04 ppm) and laboratory grease (0.07 ppm).

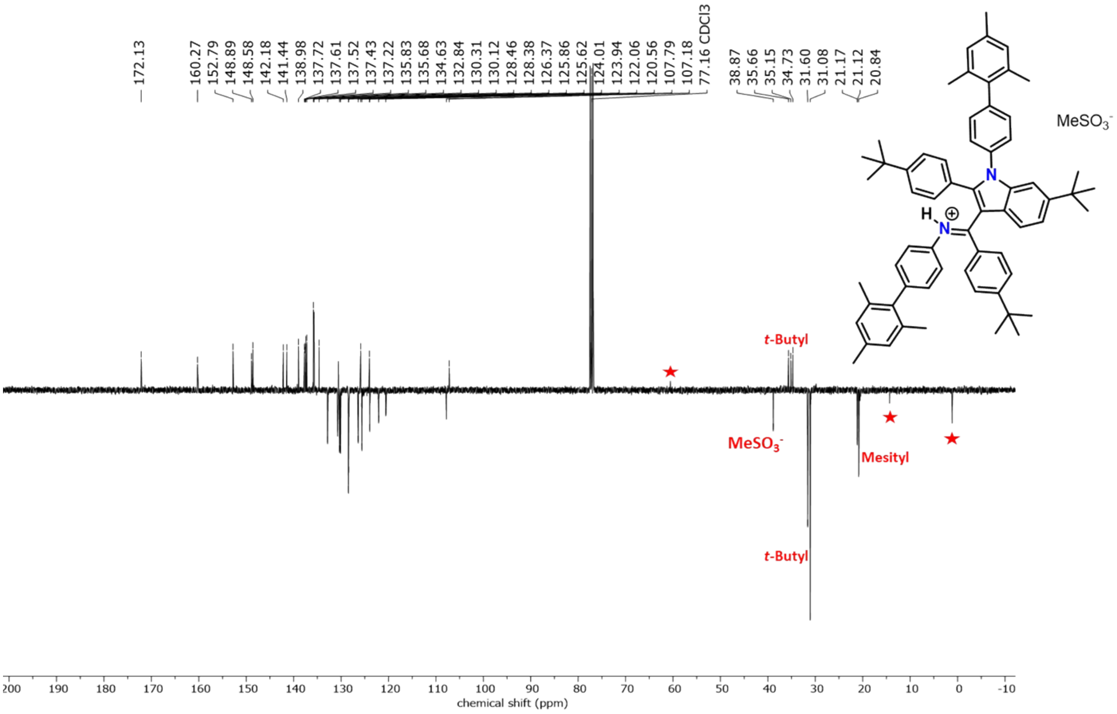

Figure S54: ^{13}C APT NMR spectrum of **^{TBP}Indole·HMeSO$_3$** in CDCl$_3$, at 25 °C. The resonances marked with a star are attributed to DCM (5.30 ppm), ethyl acetate (60.5 ppm and 14.3 ppm) and laboratory grease (1.2 ppm).

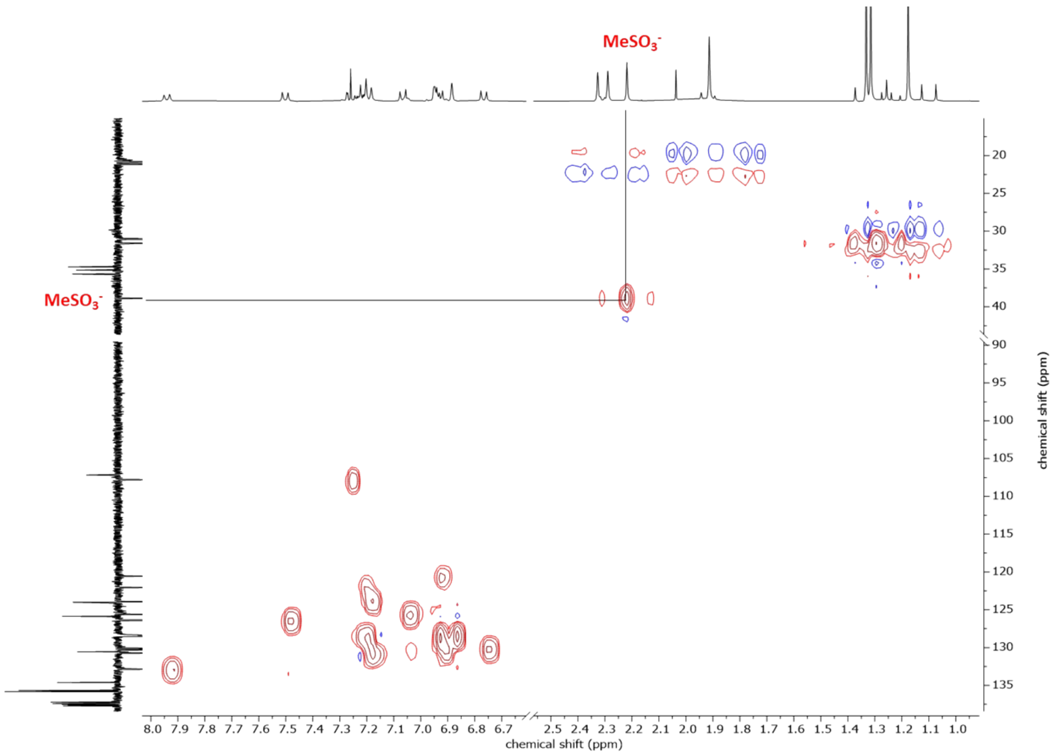

Figure S55: HSQCAD NMR spectrum of **^{TBP}Indole·HMeSO$_3$** in $CDCl_3$, at 25 °C.

Attempted Scholl oxidation of ^{TBP}BDI using $FeCl_3$

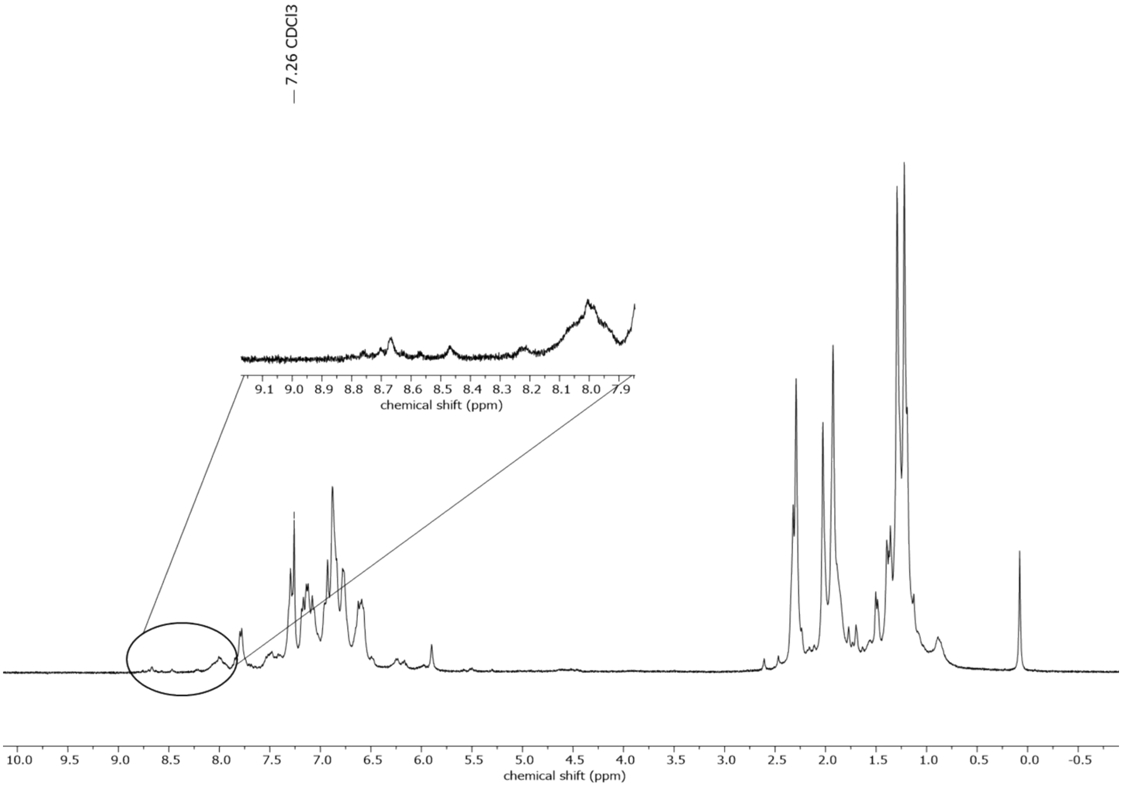

Figure S56: ^{1}H NMR spectrum of crude reaction mixture of the reaction of **^{TBP}BDI** with $FeCl_3$. The spectrum as recorded in $CDCl_3$, at 25 °C.

S4. UV-VIS and fluorescence

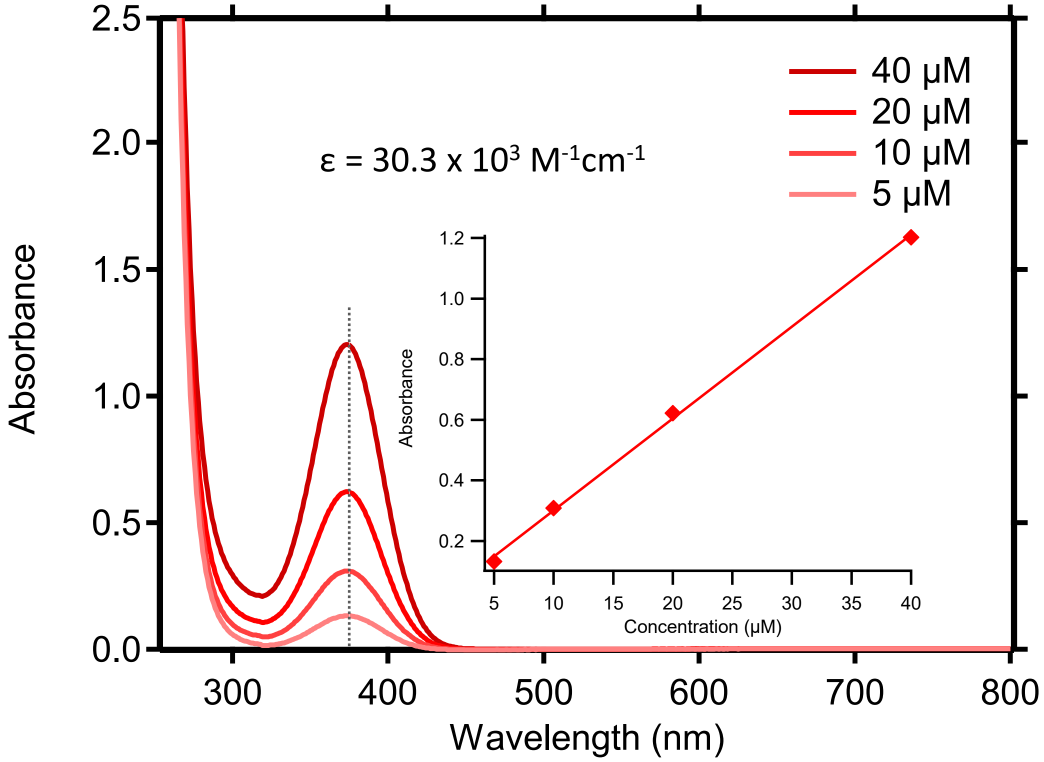

Figure S57: UV-VIS spectra of ^{TBP}**BDI-BF$_2$** in DCM at varying concentration. In-set: linear regression of the absorbance maximum at 374 nm versus the concentration.

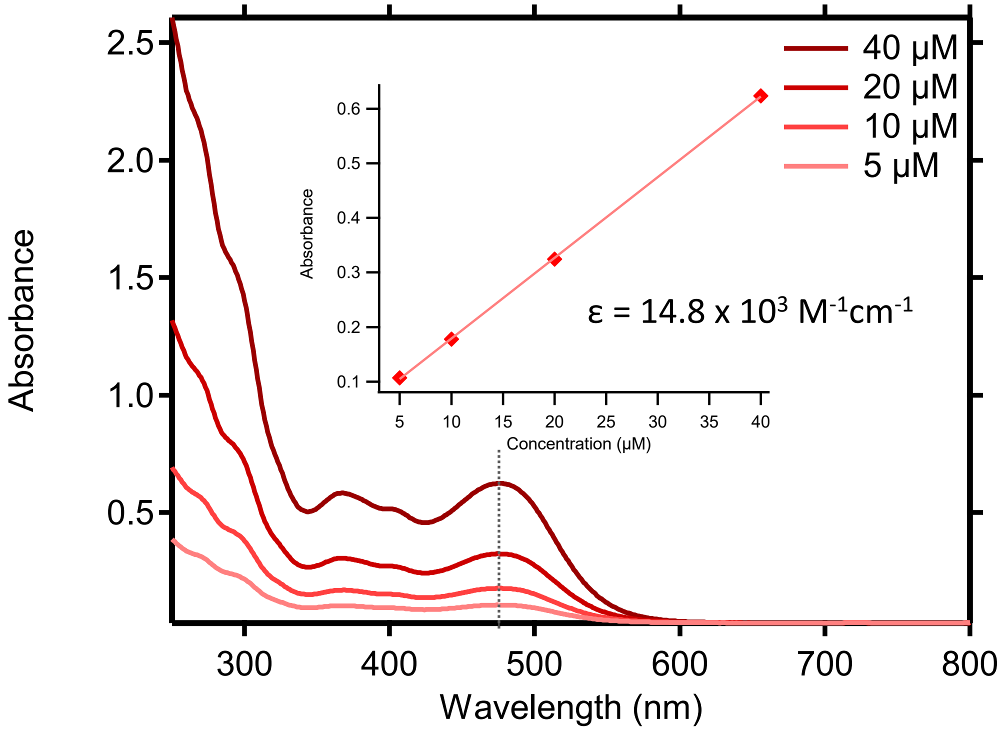

Figure S58: UV-VIS spectra of ^{tBu}**BT-BDI** in DCM at varying concentration. In-set: linear regression of the absorbance maximum at 475 nm versus the concentration.

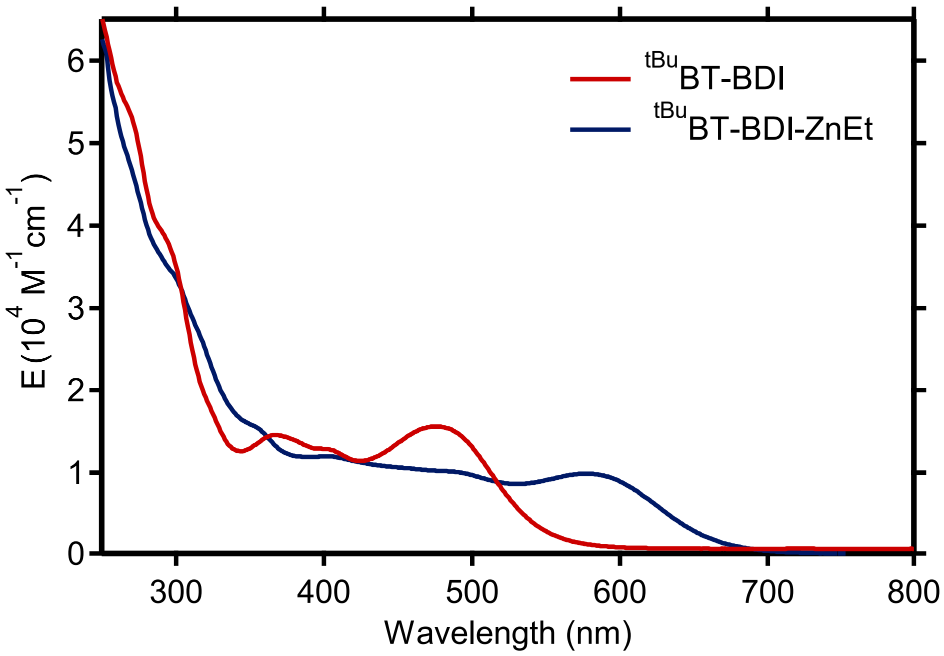

Figure S59: UV-VIS spectra of **^{tBu}BT-BDI** in DCM and **^{tBu}BT-BDI-ZnEt** in THF.

S5. Electrochemistry

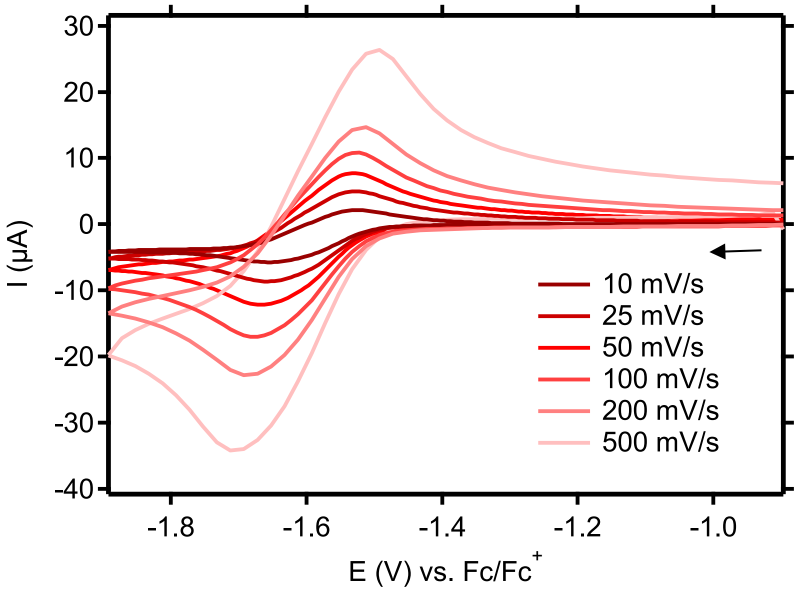

Figure S60: CV traces of **BT-BDI-BF$_2$** between -0.9 V and -1.9 V vs Fc/Fc$^+$ at multiple scan rates. Measurement was performed on 1 mM solution of analyte in DCM, 0.1 M N^{n}Bu$_4$PF$_6$ using a glassy carbon WE, a Pt wire CE and a Ag/AgNO$_3$ (10 mM) RE.

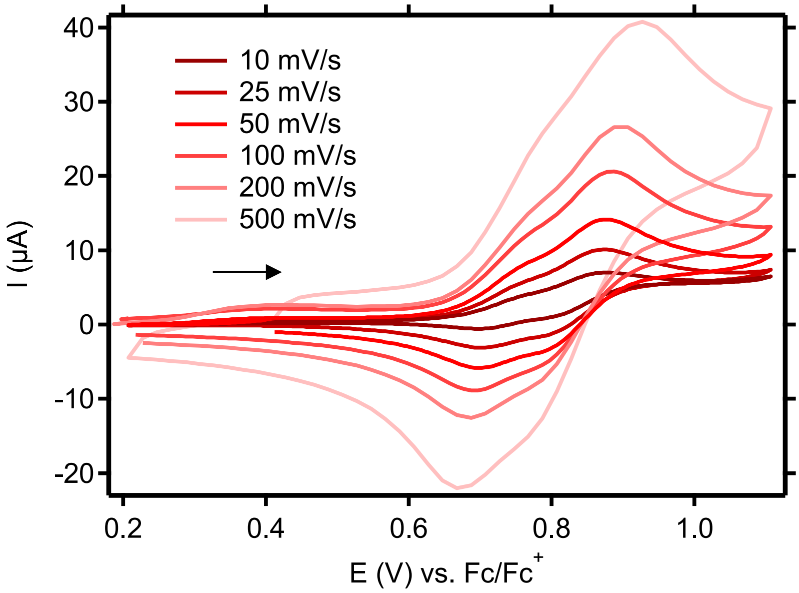

Figure S61: CV traces of **BT-BDI-BF$_2$** between 0.2 V and 1.1 V vs Fc/Fc$^+$. at multiple scan rates. Measurement was performed on 1 mM solution of analyte in DCM, 0.1 M N^{n}Bu$_4$PF$_6$ using a glassy carbon WE, a Pt wire CE and a Ag/AgNO$_3$ (10 mM) RE.

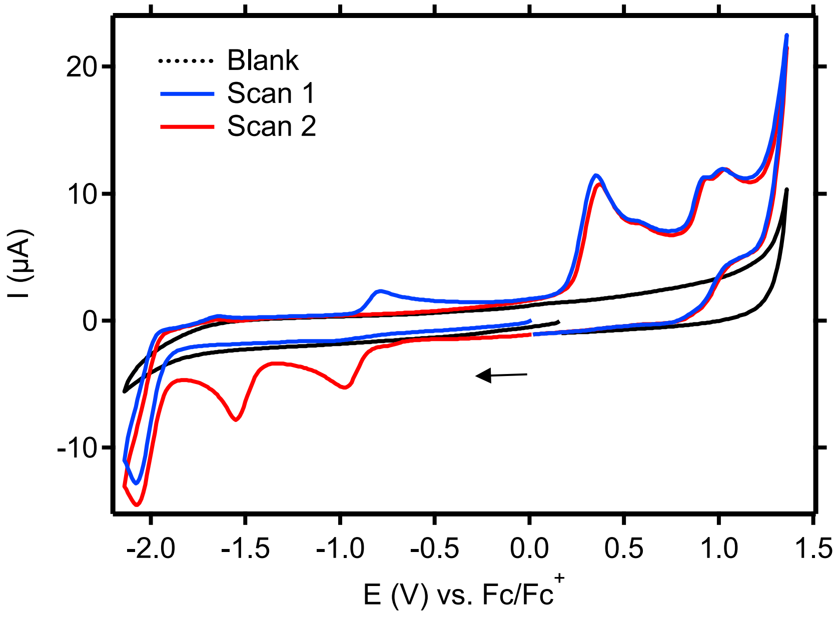

Figure S62: CV trace of ^{tBu}**BT-BDI**. Measurement was performed on 1 mM solution of analyte in DCM, 0.1 M $N^nBu_4PF_6$ using a glassy carbon WE, a Pt wire CE and a Ag/$AgNO_3$ (10 mM) RE.

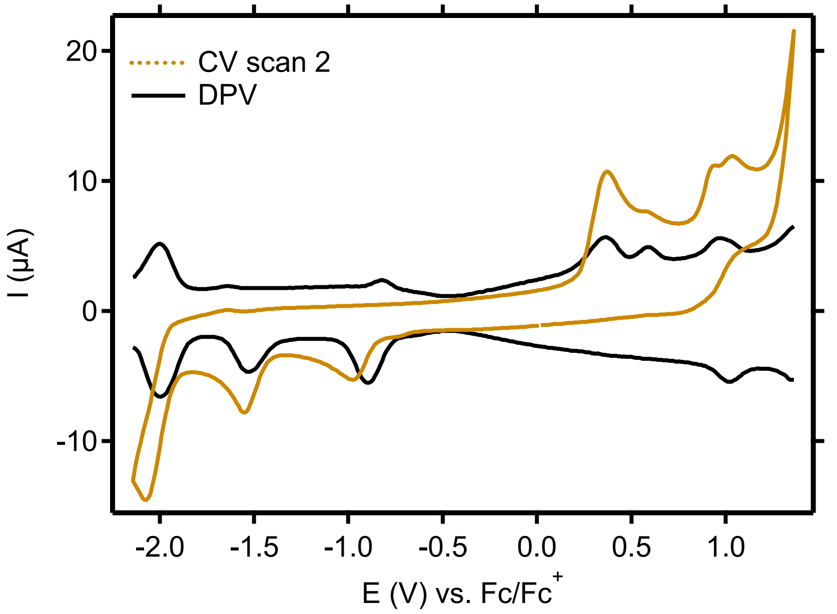

Figure S63: DPV and CV traces of ^{tBu}**BT-BDI**. Measurement was performed on 1 mM solution of analyte in DCM, 0.1 M $N^nBu_4PF_6$ using a glassy carbon WE, a Pt wire CE and a Ag/$AgNO_3$ (10 mM) RE.

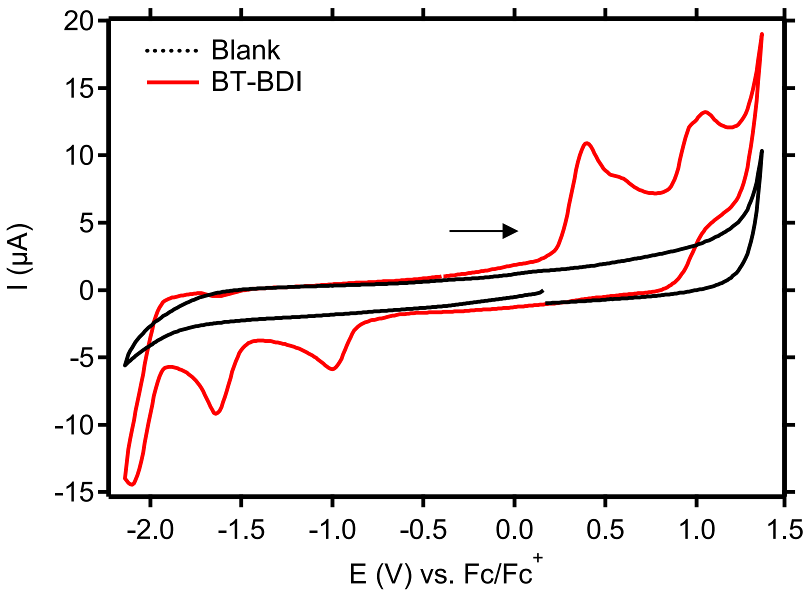

Figure S64: CV trace of **^{tBu}BT-BDI** measured in the anodic direction. Measurement was performed on 1 mM solution of analyte in DCM, 0.1 M $N^nBu_4PF_6$ using a glassy carbon WE, a Pt wire CE and a $Ag/AgNO_3$ (10 mM) RE.

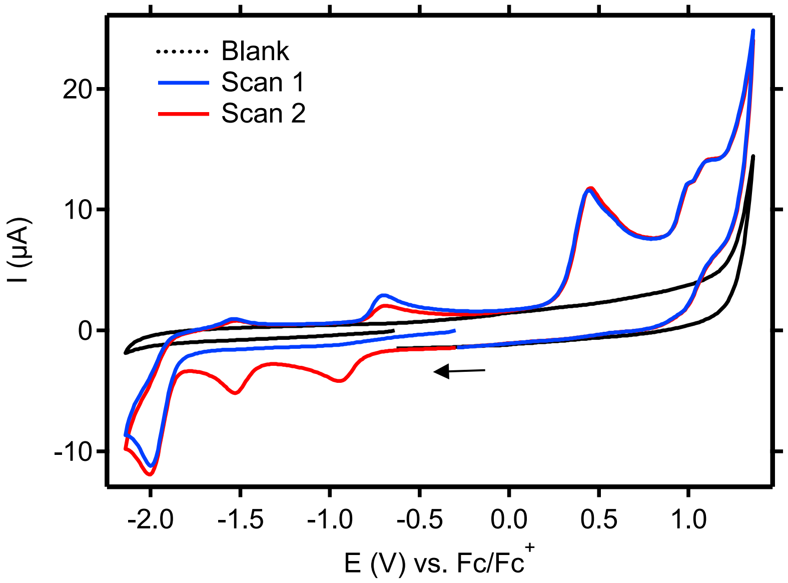

Figure S65: CV traces of **^{Cl}BT-BDI**. Measurement was performed on 1 mM solution of analyte in DCM, 0.1 M $N^nBu_4PF_6$ using a glassy carbon WE, a Pt wire CE and a $Ag/AgNO_3$ (10 mM) RE.

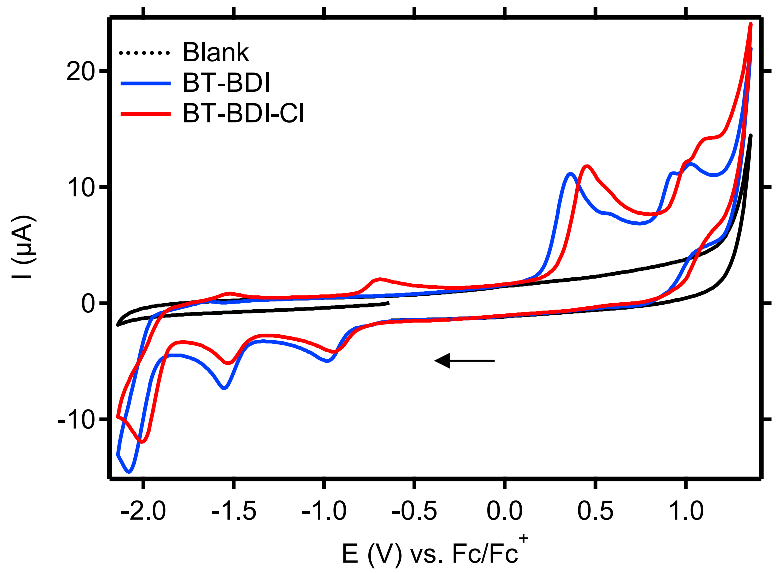

Figure S66: CV traces of ^{tBu}**BT-BDI** and ^{Cl}**BT-BDI** (both second scan). Measurement was performed on 1 mM solution of analyte in DCM, 0.1 M $N^nBu_4PF_6$ using a glassy carbon WE, a Pt wire CE and a $Ag/AgNO_3$ (10 mM) RE.

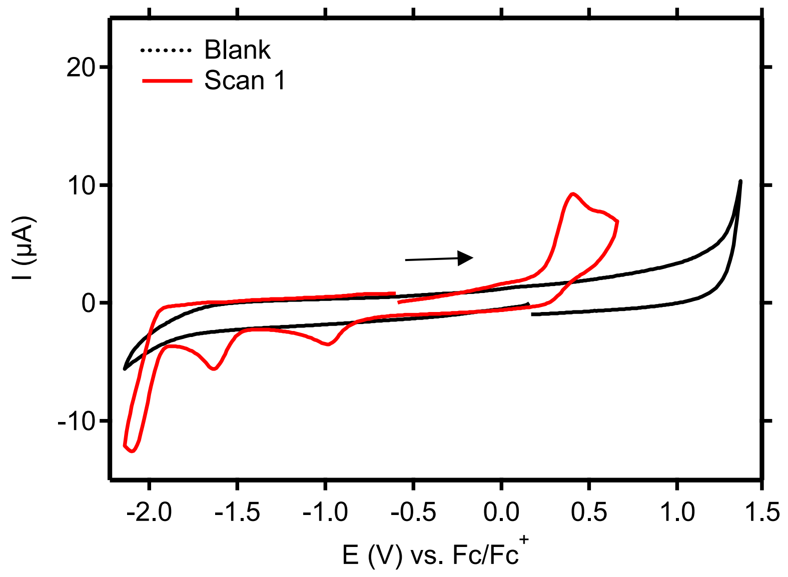

Figure S67: CV trace of ^{tBu}**BT-BDI** measured up to 0.5 V vs Fc/Fc^+ in the anodic direction. Measurement was performed on 1 mM solution of analyte in DCM, 0.1 M $N^nBu_4PF_6$ using a glassy carbon WE, a Pt wire CE and a $Ag/AgNO_3$ (10 mM) RE.

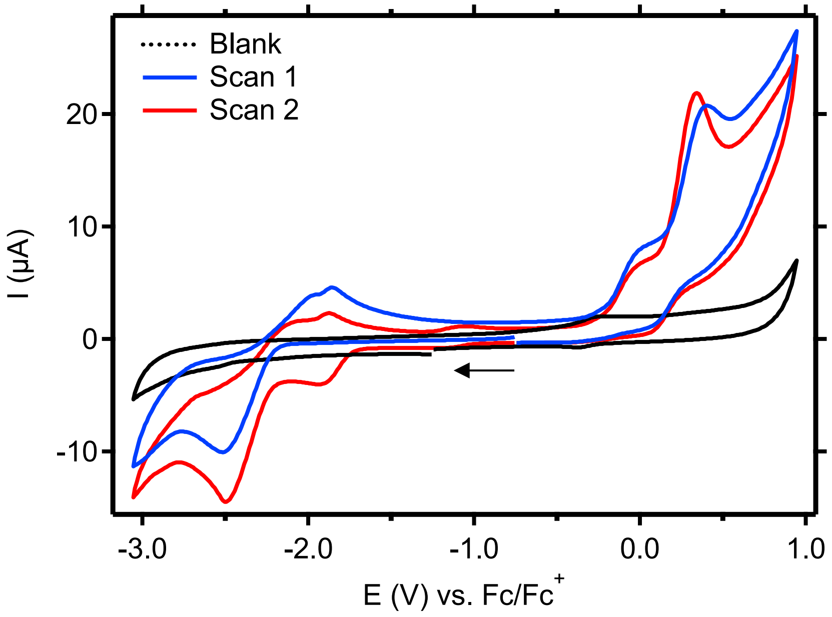

Figure S68: CV trace of **^{tBu}BT-BDI-ZnEt.** Measurement was performed on 1 mM solution of analyte in THF, 0.2 M $N^nBu_4PF_6$ using a glassy carbon WE, a Pt wire CE and a $Ag/AgNO_3$ (10 mM) RE.

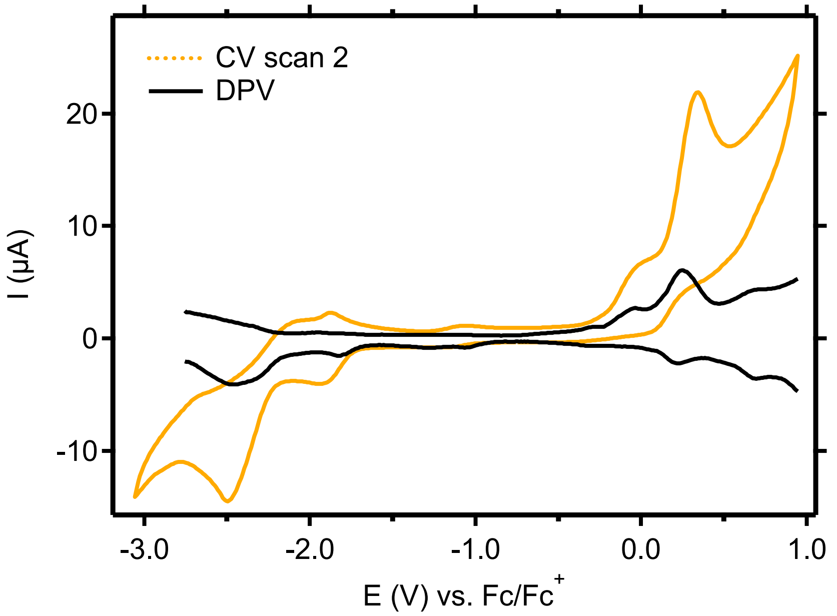

Figure S69: DPV and CV traces of **^{tBu}BT-BDI-ZnEt.** Measurement was performed on 1 mM solution of analyte in THF, 0.2 M $N^nBu_4PF_6$ using a glassy carbon WE, a Pt wire CE and a $Ag/AgNO_3$ (10 mM) RE.

S6. X-ray crystal structure determinations

^{TBP}Indole·$HMeSO_3$

$[C_{63}H_{69}N_2](CH_3SO_3) \cdot 2.5C_8Cl_2N_2O_2 \cdot C_7H_8 \cdot 2C_4H_{10}O$, Fw = 1757.16, black block, 0.50 × 0.41 × 0.24 mm^3, monoclinic, $P2_1/n$ (no. 14), a = 18.0287(5), b = 20.4042(5), c = 26.1301(5) Å, β = 97.486(1)°, V = 9530.3(4) $Å^3$, Z = 4, D_x = 1.225 g/cm^3, μ = 0.23 mm^{-1}. The diffraction experiment was performed on a Bruker Kappa ApexII diffractometer with sealed tube and Triumph monochromator (λ = 0.71073 Å) at a temperature of 150(2) K up to a resolution of $(\sin \theta/\lambda)_{max}$ = 0.61 $Å^{-1}$. The Eval15 software[8] was used for the intensity integration. The presence of diffuse streaks and additional small crystal fragments was ignored in the intensity determination. A multi-scan absorption correction and scaling was performed with SADABS[9] (correction range 0.64-0.75). A total of 175269 reflections was measured, 17722 reflections were unique (R_{int} = 0.056), 12485 reflections were observed [$I>2\sigma(I)$]. The structure was solved with Patterson superposition methods using SHELXT.[10] Structure refinement was performed with SHELXL-2019[11] on F^2 of all reflections. Non-hydrogen atoms were refined freely with anisotropic displacement parameters. C-H hydrogen atoms were introduced in calculated positions and refined with a riding model. The N-H hydrogen atom was located in difference Fourier maps and refined freely with an isotropic displacement parameter. Two t-butyl groups of the main molecule were refined with a disorder model. Additional disorder was present in a DDQ molecule on an inversion center and in a DDQ molecule on a general position. 1360 Parameters were refined with 1307 restraints (distances, angles and displacement parameters in the disordered groups and the co-crystallized solvent molecules). R1/wR2 [$I > 2\sigma(I)$]: 0.0606 / 0.1633. R1/wR2 [all refl.]: 0.0899 / 0.1867. S = 1.031. Residual electron density between -0.70 and 0.98 e/$Å^3$. Geometry calculations and checking for higher symmetry was performed with the PLATON program.[12]

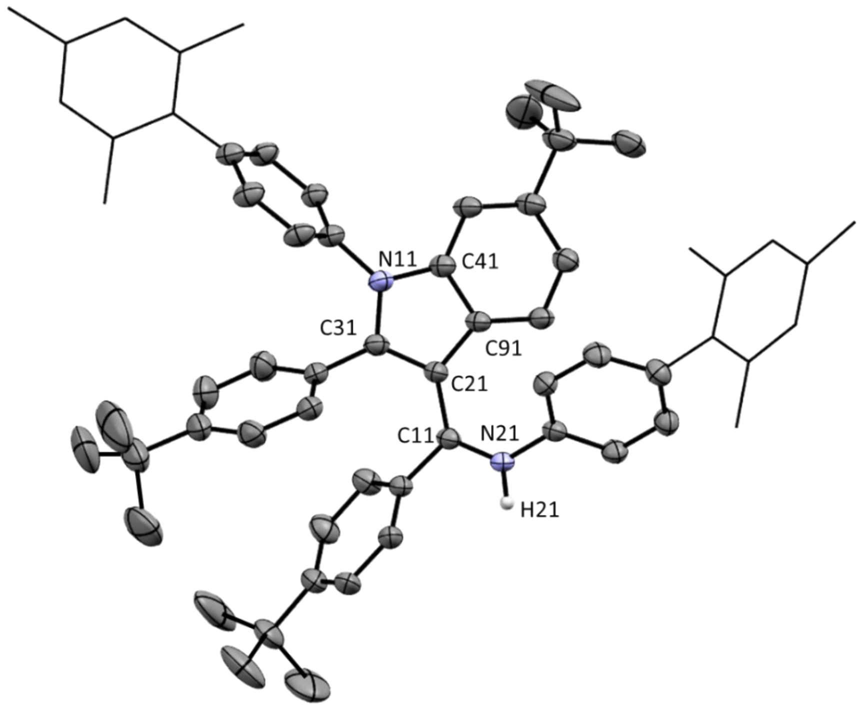

Figure S70: Displacement ellipsoid plot (50 % probability) of the asymmetric unit of **^{TBP}Indole·$HMeSO_3$**. Most hydrogen atoms, co-crystallized DDQ, toluene and Et_2O molecules, as well as $MeSO_3^-$ counterion are omitted; mesityl groups are shown as wireframe for clarity. Only the major disorder component is shown.

Table S1: Selected bond distances in the crystal structure of ^{TBP}**Indole·HMeSO$_3$**.

Bond	Distance (Å)
**N11-C31**	1.375(3)
**N11-C41**	1.396(3)
**N21-C11**	1.309(3)
**C11-C21**	1.445(3)
**C21-C91**	1.450(3)
**C41-C91**	1.403(3)

^{TBP}BDI-BF$_2$

$C_{63}H_{69}BF_2N_2$ + disordered solvent, Fw = 903.01*, colourless block, 0.62 × 0.23 × 0.20 mm^3, monoclinic, P2$_1$/n (no. 14), a = 12.7674(6), b = 25.8067(10), c = 19.4442(9) Å, β = 92.287(2)°, V = 6401.4(5) Å^3, Z = 4, D_x = 0.937 g/cm^{3*}, μ = 0.06 mm^{-1*}. The diffraction experiment was performed on a Bruker Kappa ApexII diffractometer with sealed tube and Triumph monochromator (λ = 0.71073 Å) at a temperature of 150(2) K up to a resolution of $(\sin \theta/\lambda)_{max}$ = 0.65 Å^{-1}. The Eval15 software[8] was used for the intensity integration. A multi-scan absorption correction and scaling was performed with SADABS[9] (correction range 0.58-0.75). A total of 78517 reflections was measured, 14718 reflections were unique (R_{int} = 0.052), 8554 reflections were observed [$I>2\sigma(I)$]. The structure was solved with Patterson superposition methods using SHELXT.[10] Structure refinement was performed with SHELXL-2018[11] on F^2 of all reflections. Non-hydrogen atoms were refined freely with anisotropic displacement parameters. Hydrogen atoms were introduced in calculated positions and refined with a riding model. The BF_2 moiety and one t-butyl group were refined with a disorder model. The structure contains large voids (1361 Å^3 / unit cell) filled with disordered CH_2Cl_2 molecules. Their contribution to the structure factors was secured by back-Fourier transformation using the SQUEEZE algorithm[13] resulting in 494 electrons / unit cell. 684 Parameters were refined with 190 restraints (distances, angles and displacement parameters in the disordered groups). R1/wR2 [$I > 2\sigma(I)$]: 0.0519 / 0.1357. R1/wR2 [all refl.]: 0.0916 / 0.1541. S = 1.066. Residual electron density between -0.18 and 0.20 e/Å^3. Geometry calculations and checking for higher symmetry was performed with the PLATON program.[12]

* Derived values do not contain the contribution of the disordered solvent.

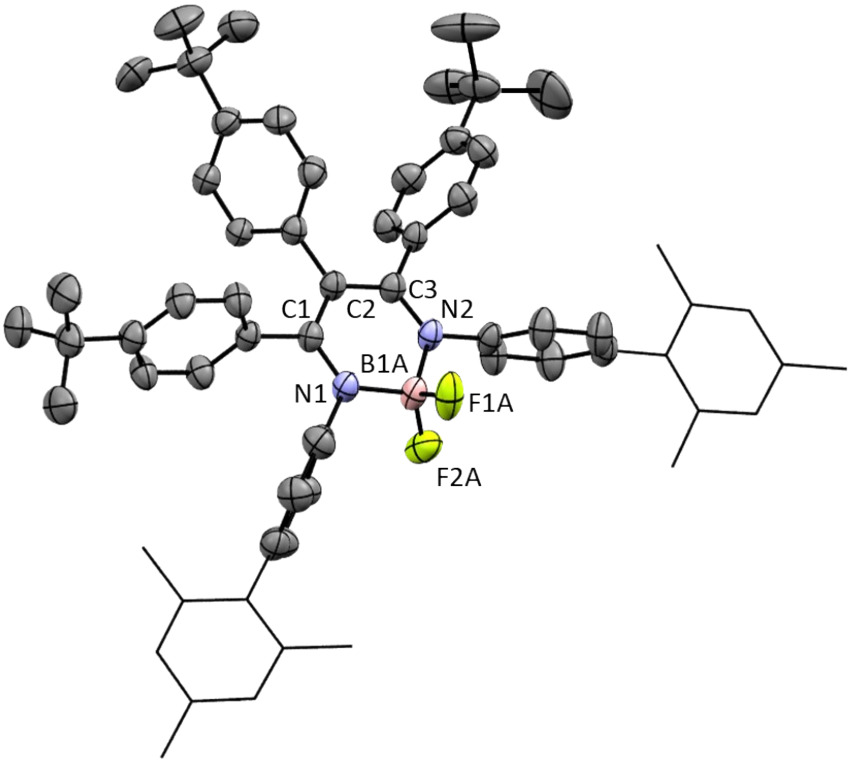

Figure S71: Displacement ellipsoid plot (50 % probability) of the asymmetric unit of ^{TBP}**BDI-BF$_2$**. Hydrogen atoms are omitted; mesityl groups are shown as wireframe for clarity. Only the major disorder component is shown.

Table S2: Selected bond distances and angles in the crystal structure of **^{TBP}BDI-BF$_2$.** Only the major disorder component of BF_2 is discussed.

Bond	Distance (Å) or angle (°)
**B1A-F1A**	1.391(5)
**B1A-F2A**	1.387(5)
**B1A-N1**	1.545(4)
**B1A-N2**	1.554(4)
**N1-C1**	1.341(2)
**N2-C3**	1.333(2)
**C1-C2**	1.403(2)
**C2-C3**	1.414(2)
**N1 – B1A – F1A**	108.1(7)
**N1 – B1A – F2A**	111.3(8)
**N2 – B1A – F1A**	110.6(9)
**N2 – B1A – F2A**	112.0(7)

BT-BDI-BF_2

$C_{60.68}H_{59.79}BCl_{0.58}F_2N_2$ + disordered solvent, Fw = 886.45*, orange block, 0.29 × 0.24 × 0.19 mm^3, monoclinic, I2/a (no. 15), a = 17.4445(5), b = 20.8422(6), c = 34.9654(8) Å, β = 95.518(1)°, V = 12653.9(6) $Å^3$, Z = 8, D_x = 0.931 g/cm^3*, μ = 0.08 mm^{-1}*. The diffraction experiment was performed on a Bruker Kappa ApexII diffractometer with sealed tube and Triumph monochromator (λ = 0.71073 Å) at a temperature of 150(2) K up to a resolution of $(\sin \theta/\lambda)_{max}$ = 0.61 $Å^{-1}$. The Eval15 software[8] was used for the intensity integration. A multi-scan absorption correction and scaling was performed with SADABS[9] (correction range 0.68-0.75). A total of 88224 reflections was measured, 11775 reflections were unique (R_{int} = 0.034), 7708 reflections were observed [I>2σ(I)]. The structure was solved with Patterson superposition methods using SHELXT.[10] Structure refinement was performed with SHELXL-2018[11] on F^2 of all reflections. Non-hydrogen atoms were refined freely with anisotropic displacement parameters. Hydrogen atoms were introduced in calculated positions and refined with a riding model. The main molecule showed substitutional disorder between a chloro substituent (57.9(4)% occupancy) and a t-butyl substituent (42.1(4)% occupancy). Another t-butyl group was refined with a model for rotational disorder. The structure contains large voids (3462 $Å^3$ / unit cell) filled with disordered methyl-t-butyl ether (MTBE) molecules. Their contribution to the structure factors was secured by back-Fourier transformation using the SQUEEZE algorithm[13] resulting in 843 electrons / unit cell. 651 Parameters were refined with 272 restraints (distances, angles and displacement parameters in the disordered groups). R1/wR2 [I > 2σ(I)]: 0.0600 / 0.1867. R1/wR2 [all refl.]: 0.0830 / 0.2038. S = 1.031. Residual electron density between -0.34 and 0.52 e/$Å^3$. Geometry calculations and checking for higher symmetry was performed with the PLATON program.[12]

* Derived values do not contain the contribution of the disordered solvent.

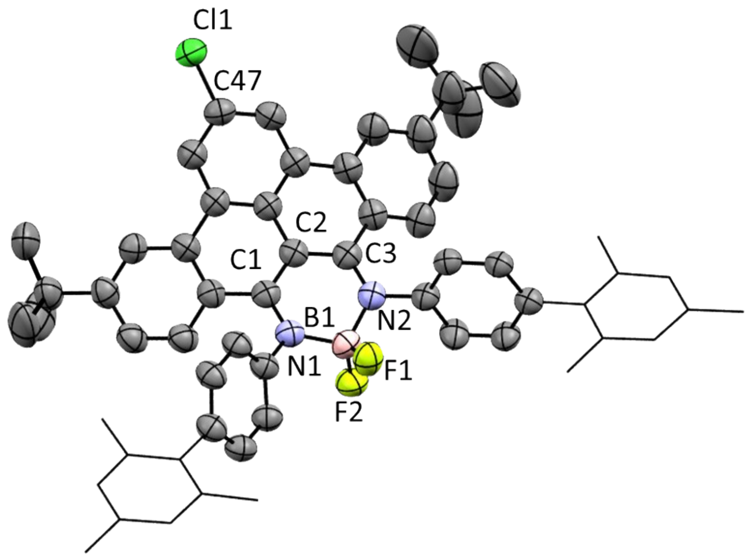

Figure S72: Displacement ellipsoid plot (50 % probability) of the asymmetric unit of **BT-BDI-BF_2**. Hydrogen atoms are omitted; mesityl groups are shown as wireframe for clarity. Severely disordered methyl t-butyl ether molecules are omitted. There is substitutional disorder for the substituents at C47 between chlorine and t-butyl in a ratio of 57.9(4):42.1(4).

Table S3: Selected bond distances and angles the crystal structure of **BT-BDI-BF$_2$.**

Bond	Distance (Å) or Angle (°)
**B1-F1**	1.388(3)
**B1-F2**	1.387(3)
**B1-N1**	1.535(3)
**B1-N2**	1.544(3)
**N1-C1**	1.345(3)
**N2-C3**	1.349(3)
**C1-C2**	1.417(3)
**C2-C3**	1.422(3)
**N1 – B1 – F1**	109.34(19)
**N1 – B1 – F2**	109.92(19)
**N2 – B1 – F1**	109.07(19)
**N2 – B1 – F2**	109.61(19)

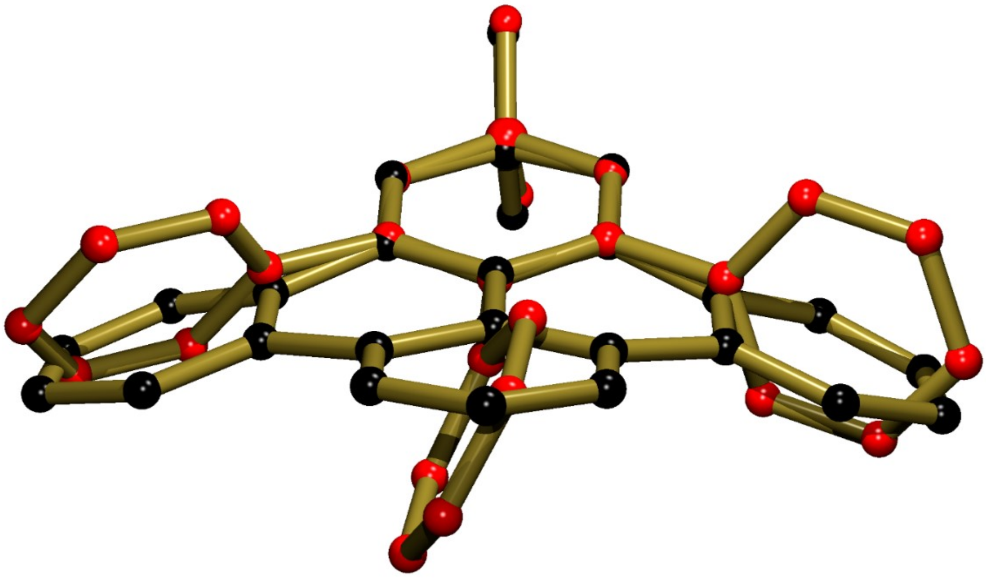

Figure S73: Overlay of the molecular cores of [TBP]**BDI-BF$_2$** (red) and **BT-BDI-BF$_2$** (black).

In Figure S74, metrics describing the curvature of the benzo[*f,g*]tetracene backbone are shown. Firstly, the distance between C57 and C37, which is 9.636(4) Å. In fully planar tetracene, the analogous distances are 9.738(5) and 9.754(5) Å for the two independent molecules in the unit cell.[14] Secondly, the distances between the average plane defined by C37, C38, C56, and C57 and C2 or C44, which are 1.022(2) Å and 0.878(2) Å respectively. These distances effectively show the "bowl depth" at these points.

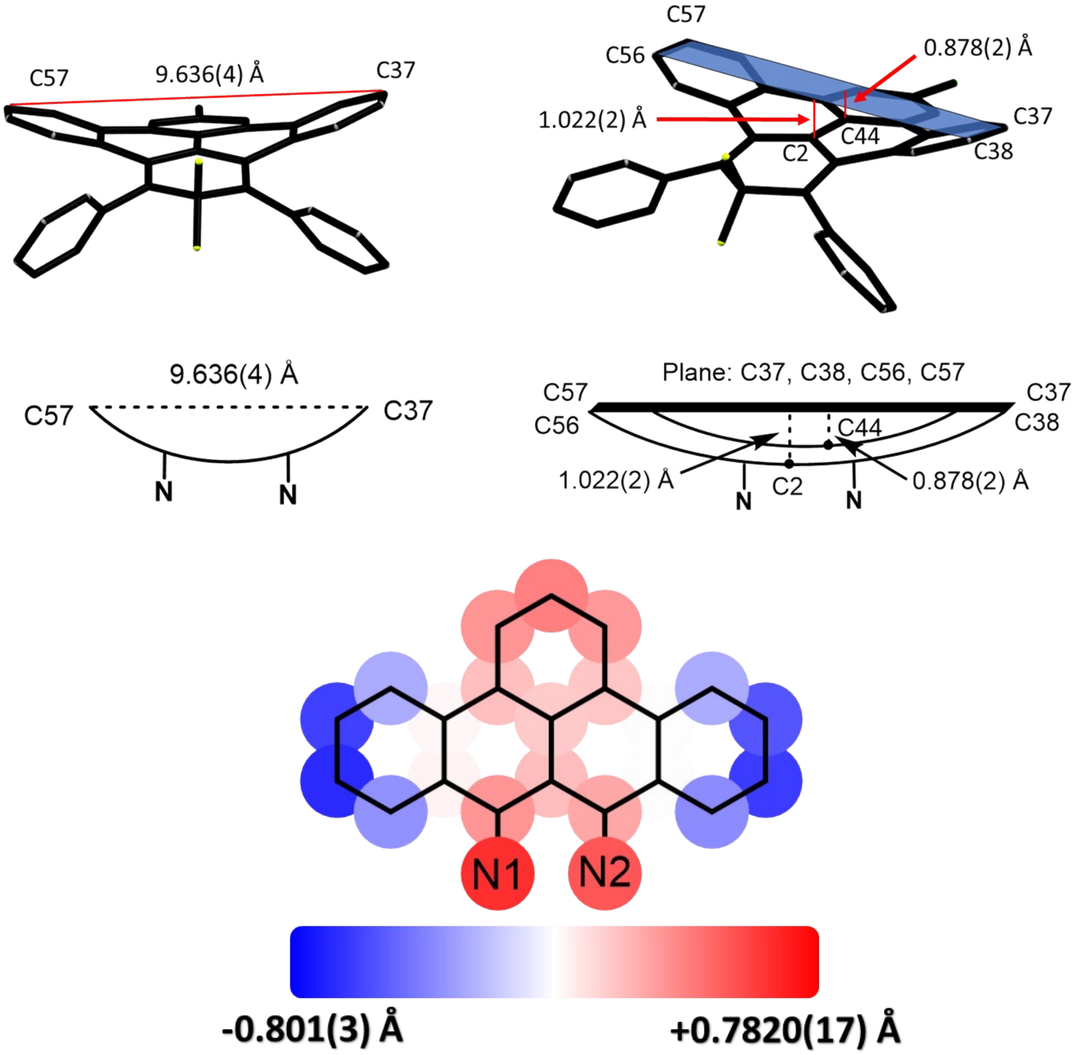

Figure S74: Depiction of the distances showing the curvature of the benzo[*f,g*]tetracene framework of in the crystal structure of **BT-BDI-BF$_2$** (top). A visual representation of the curvature of the **BT-BDI** backbone in the crystal structure of **BT-BDI-BF$_2$** is shown, defined by the distance between the atoms and the average plane through the all the atoms shown (bottom).

The substituents on the α-nitrogen, derived from 4-mesityl aniline, point down from the mean plane of the binding pocket. Centroids defined by the 6 aniline-derived carbon atoms on either side of the molecule (C4, C5, C6, C7, C8, C9 & C19, C20, C21, C22, C23, C24) and the average plane defined by the binding pockets (B1, C1, C2, C3, N1, N2) have distances of 0.9414(8) and 0.7336(9) Å, respectively as shown in Figure S75.

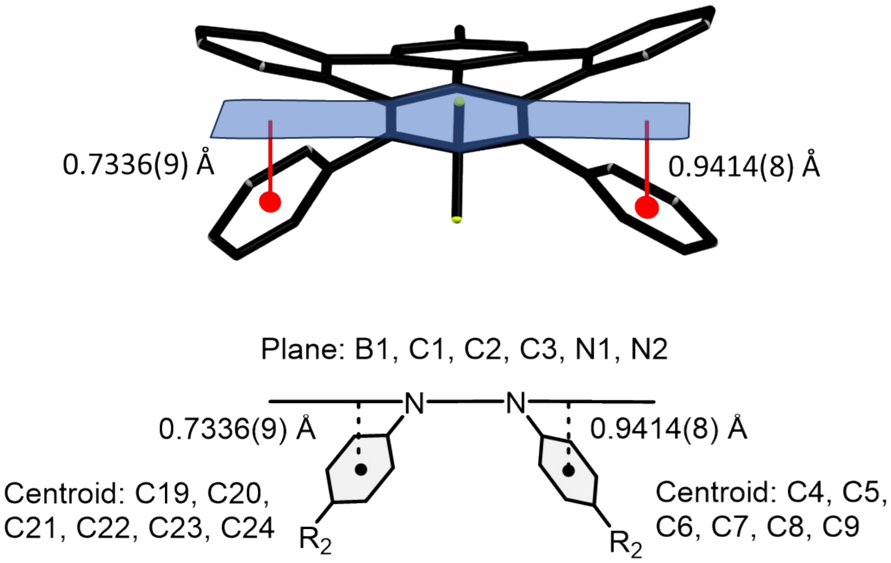

Figure S75: Depiction of the distances showing the bending of the 4-mesityl aniline-derived groups on the α-nitrogens out of the plane of the binding pocket in the crystal structure of **BT-BDI-BF$_2$**.

$[C_{59.87}H_{57.96}BCl_{0.78}F_2N_2][C_{60.70}H_{59.83}BCl_{0.58}F_2N_2] \cdot C_4H_{10}O$, Fw = 1842.58, orange block, 0.34 × 0.17 × 0.16 mm^3, triclinic, P**Error!** (no. 2), a = 13.9298(4), b = 16.9644(5), c = 22.9339(10) Å, α = 87.993(1), β = 89.410(1), γ = 71.606(1) °, V = 5139.5(3) $Å^3$, Z = 2, D_x = 1.191 g/cm^3, μ = 0.11 mm^{-1}. The diffraction experiment was performed on a Bruker Kappa ApexII diffractometer with sealed tube and Triumph monochromator (λ = 0.71073 Å) at a temperature of 150(2) K up to a resolution of $(\sin \theta/\lambda)_{max}$ = 0.61 $Å^{-1}$. The Eval15 software[8] was used for the intensity integration. A multi-scan absorption correction and scaling was performed with SADABS[9] (correction range 0.68-0.75). A total of 93386 reflections was measured, 19145 reflections were unique (R_{int} = 0.041), 11739 reflections were observed [$I>2\sigma(I)$]. The structure was solved with Patterson superposition methods using SHELXT.[10] Structure refinement was performed with SHELXL-2018[11] on F^2 of all reflections. Non-hydrogen atoms were refined freely with anisotropic displacement parameters. Hydrogen atoms were introduced in calculated positions and refined with a riding model. There are two independent molecules in the asymmetric unit which both show substitutional disorder between chlorine and t-butyl substituents. In one molecule, the ratio Cl:t-butyl is 78.2(5):21.8(5) and in the other molecule the ratio is 57.5(4):42.5(4). Additional disorder is in the co-crystallized diethyl ether molecule. 1345 Parameters were refined with 747 restraints (distances, angles and displacement parameters in the disordered groups). R1/wR2 [$I > 2\sigma(I)$]: 0.0684 / 0.1979. R1/wR2 [all refl.]: 0.1109 / 0.2263. S = 1.057. Residual electron density between -0.39 and 0.56 e/$Å^3$. Geometry calculations and checking for higher symmetry was performed with the PLATON program.[12]

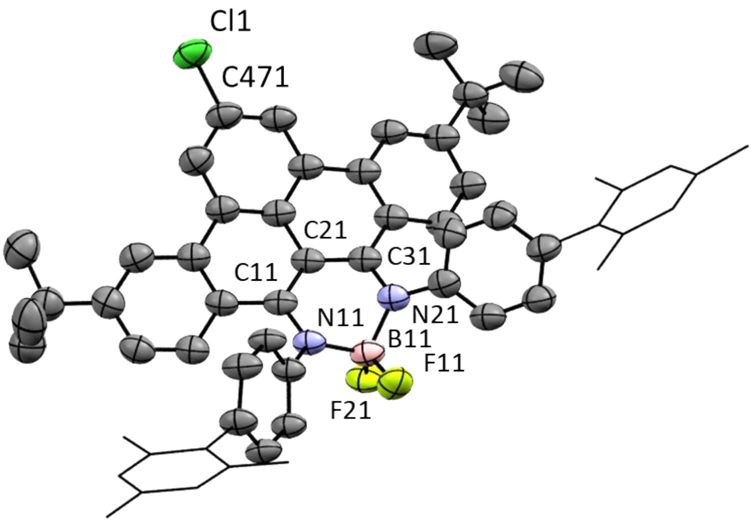

Figure S76: Displacement ellipsoid plot of **BT-BDI-BF₂** in the crystal (50 % probability level). Only one of two independent molecules is shown. There is substitutional disorder at C471 between chlorine and t-butyl in a ratio of 78.2(5):21.8(5). Hydrogen atoms and disordered diethyl ether are omitted in the drawing.

Table S4: Selected bond distances and angles in the crystal structure of **BT-BDI-BF_2.** The two independent molecules are indicated by x=1 or x=2.

Bond	Distance (Å) or Angle (°) x=1	Distance (Å) or Angle (°) x=2
**B1x-F1x**	1.365(4)	1.390(4)
**B1x-F2x**	1.392(4)	1.369(4)
**B1x-N1x**	1.552(5)	1.544(4)
**B1x-N2x**	1.539(4)	1.530(4)
**N1x-C1x**	1.358(4)	1.351(3)
**N2x-C3x**	1.358(4)	1.354(3)
**C1x-C2x**	1.429(4)	1.421(4)
**C2x-C3x**	1.418(4)	1.419(4)
**N1x – B1x – F1x**	110.5(3)	108.6(3)
**N1x – B1x – F2x**	109.2(3)	109.9(3)
**N2x – B1x – F1x**	111.4(3)	109.6(3)
**N2x – B1x – F2x**	108.8(3)	110.6(3)

S7. IR spectra

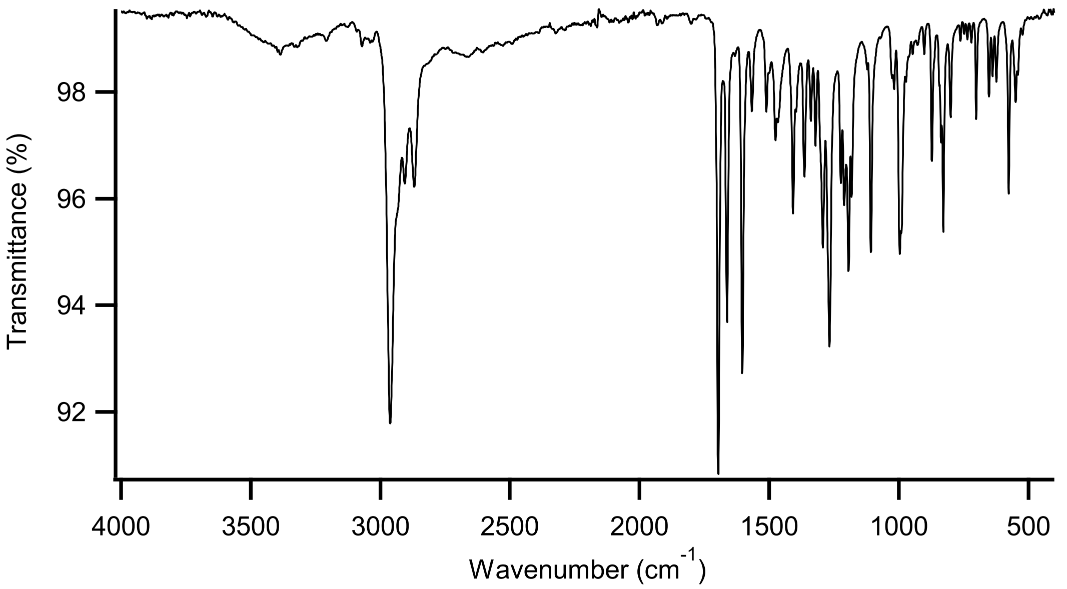

Figure S77: ATR-IR spectrum of 1,2,3-tris(4-(tert-butyl)phenyl)propane-1,3-dione (^{TBP}**AcAc**).

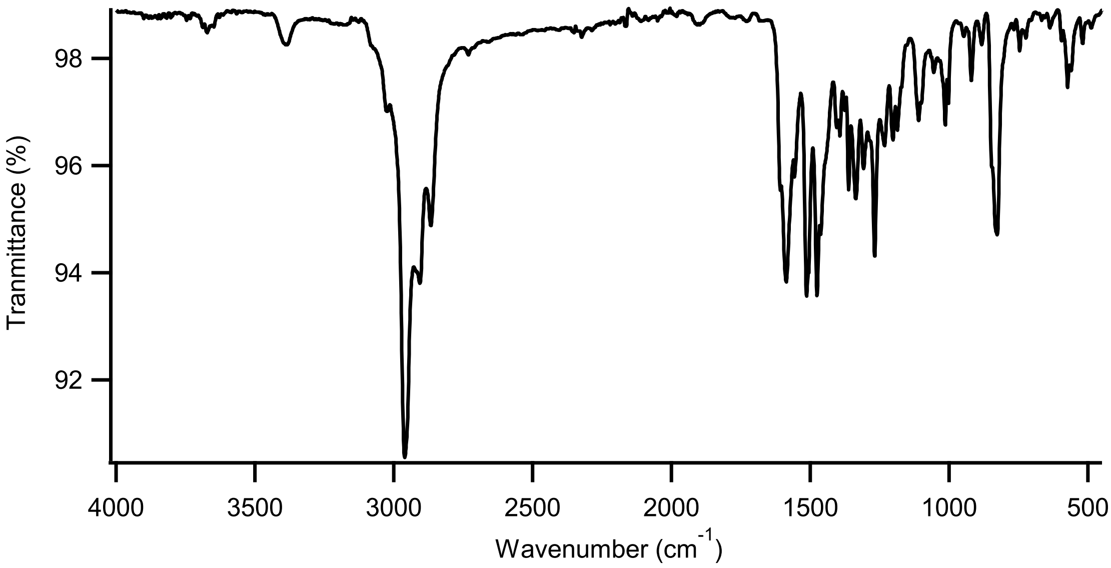

Figure S78: ATR-IR spectrum of ^{TBP}**BDI**.

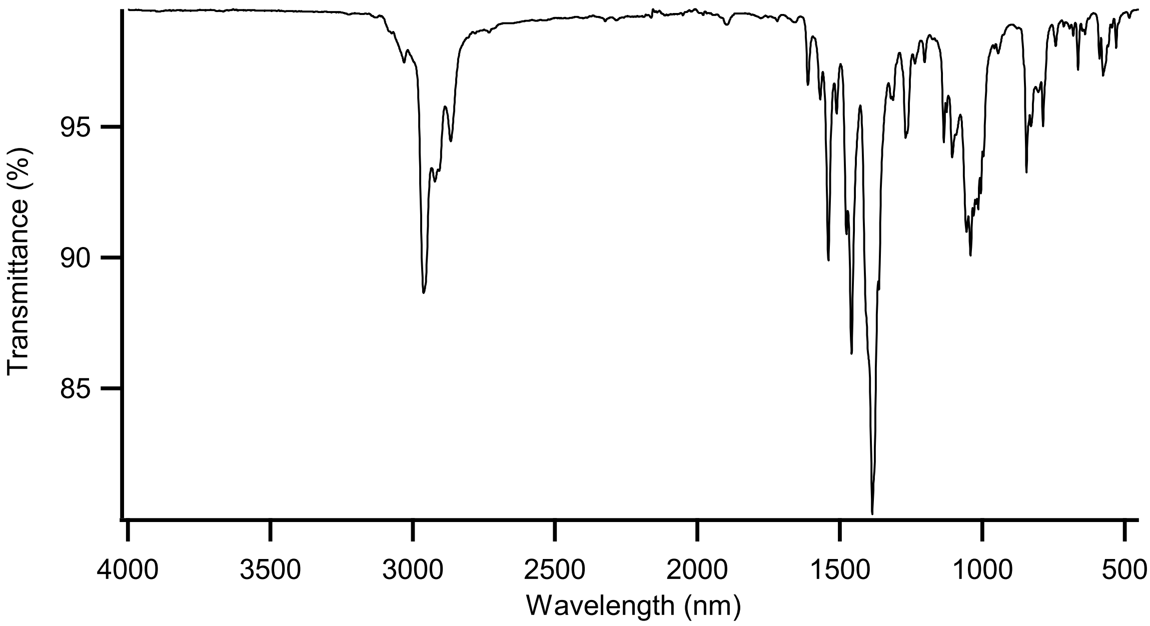

Figure S79: ATR-IR spectrum of ^{TBP}**BDI-BF$_2$**.

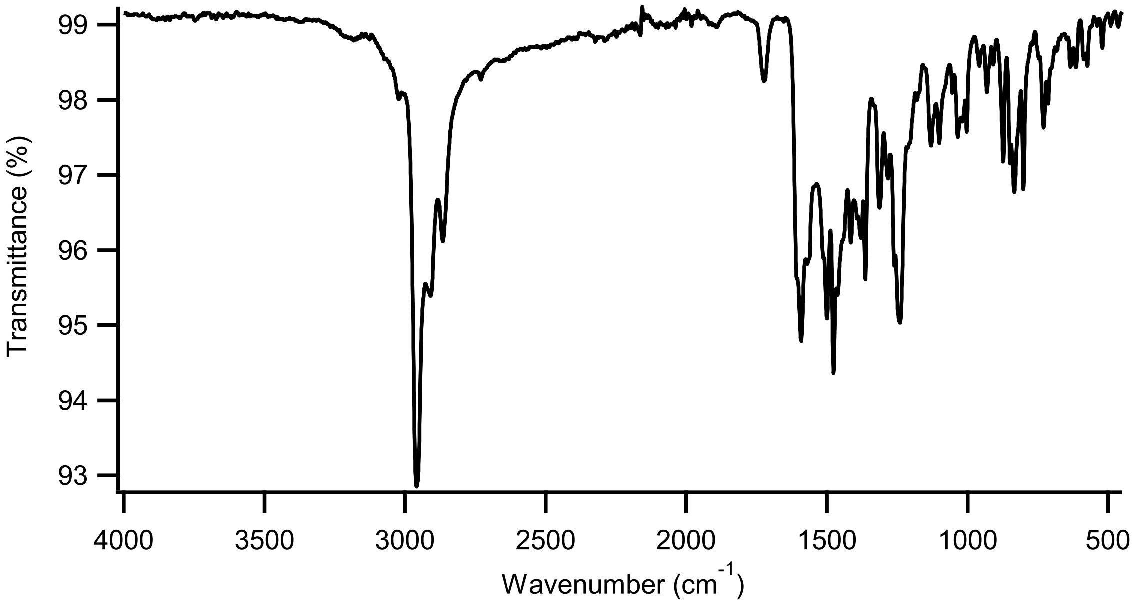

Figure S80: ATR-IR spectrum of ^{tBu}**BT-BDI**.

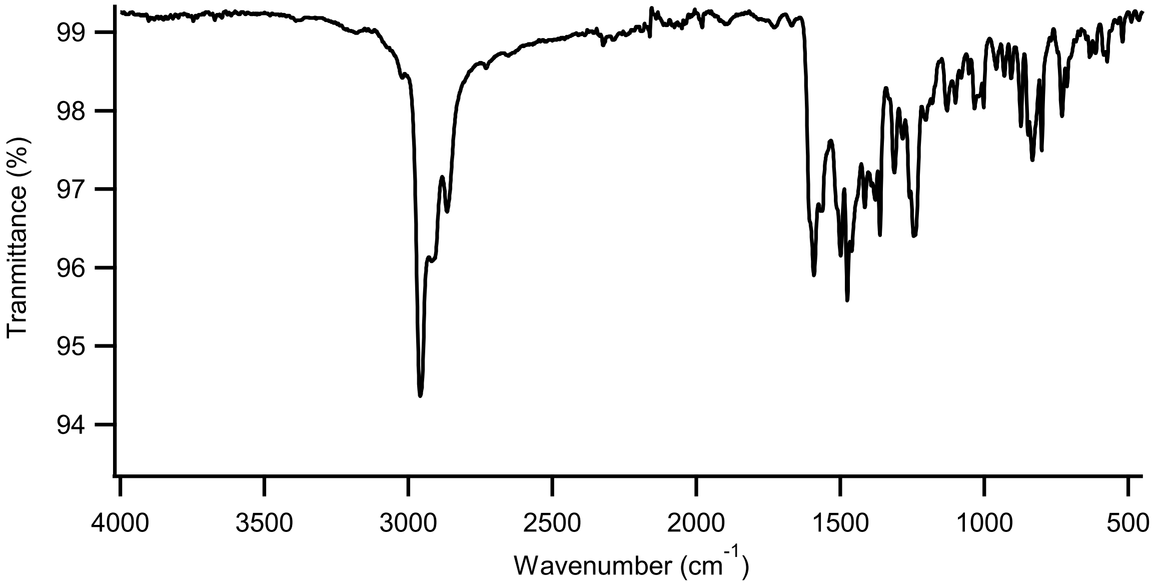

Figure S81: ATR-IR spectrum of ^{Cl}**BT-BDI**.

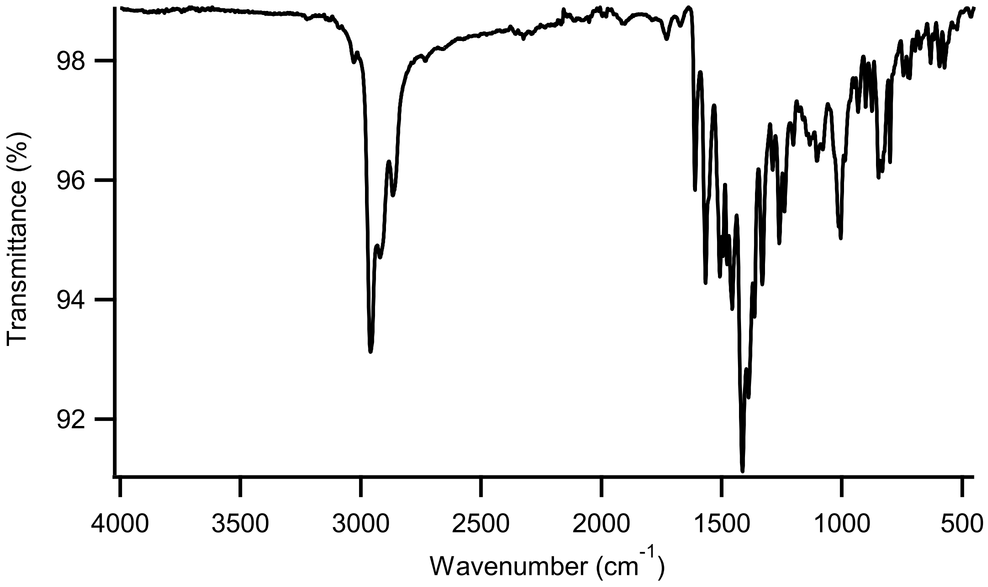

Figure S82: ATR-IR spectrum of **BT-BDI-BF$_2$**.

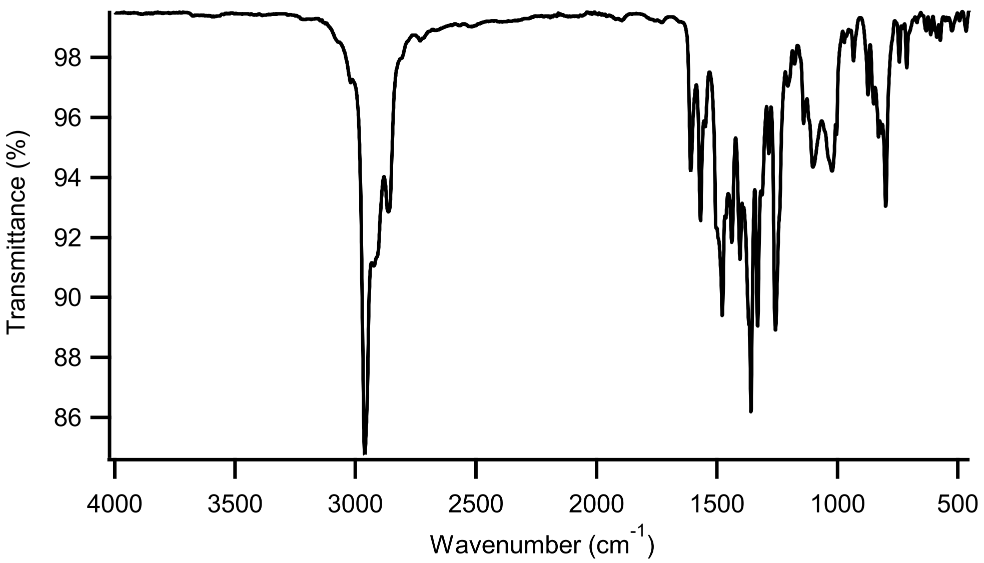

Figure S83: ATR-IR spectrum of tBu**BT-BDI-ZnEt**.

S8. HRMS spectra

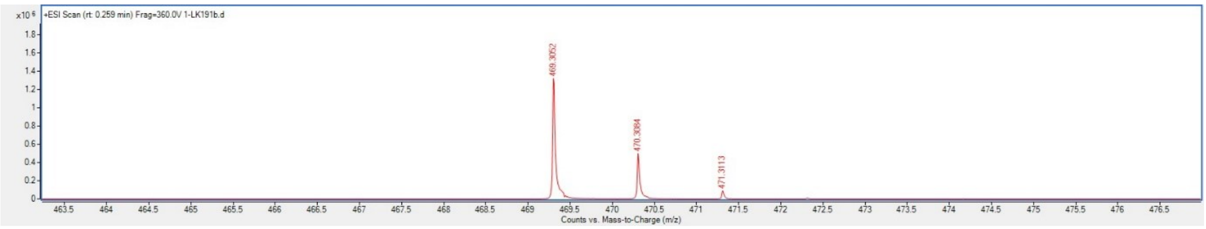

Figure S84: HRMS spectrum of **^{TBP}AcAc** {$[M+H]^+$, calc. 469.3107}.

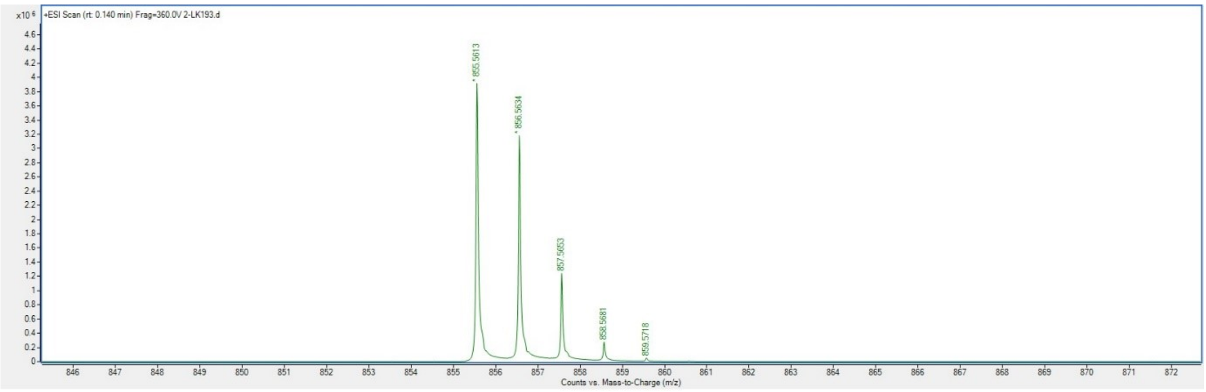

Figure S85: HRMS spectrum of **^{TBP}BDI** {$[M+H]^+$, calc. 855.5617}.

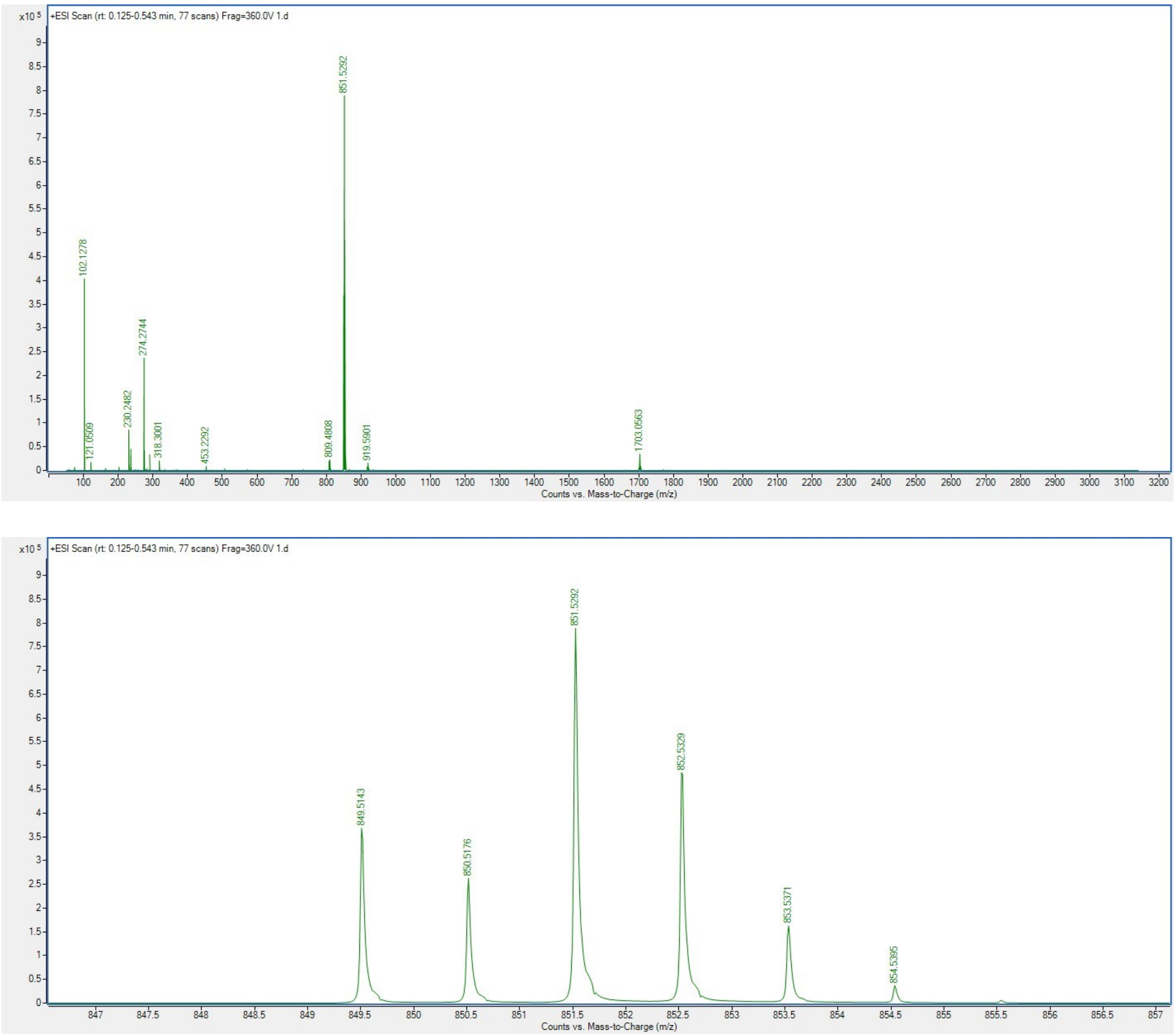

Figure S86: HRMS spectrum of ^{tBu}**BT-BDI** {[M+H]$^+$, calc. 851.5304}.

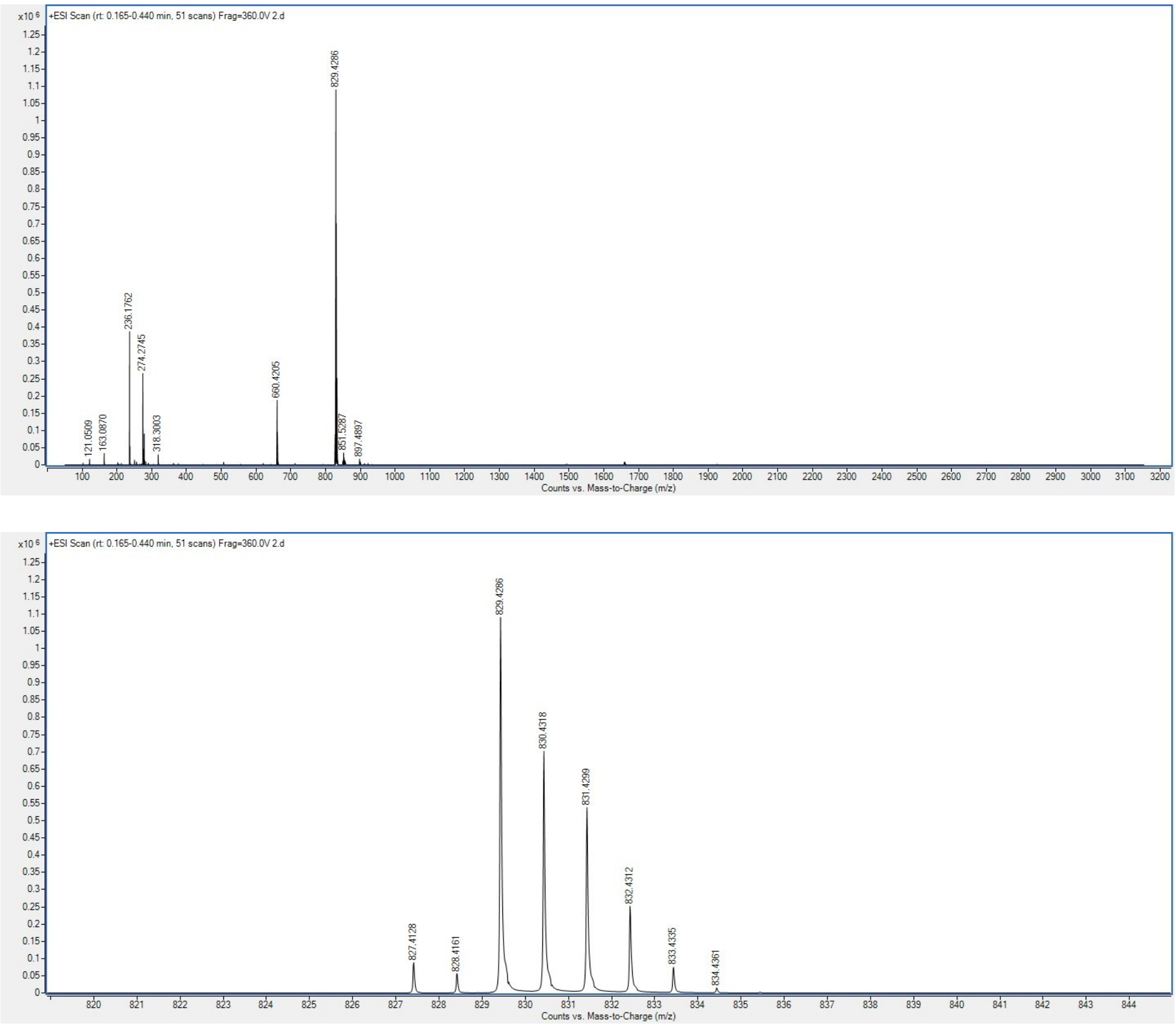

Figure S87: HRMS spectrum of **^{Cl}BT-BDI** {$[M+H]^+$, calc. 829.4289}.

References

1 G. R. Fulmer, A. J. M. Miller, N. H. Sherden, H. E. Gottlieb, A. Nudelman, B. M. Stoltz, J. E. Bercaw and K. I. Goldberg, *Organometallics*, 2010, **29**, 2176–2179.

2 X. Ren and H. Du, *J. Am. Chem. Soc.*, 2016, **138**, 810–813.

3 G. Zhang, Q. Liang, W. Yang, S. Jiang, Z. Wang, C. Zhang and G. Zhang, *Adv. Synth. Catal.*, 2022, **364**, 2951–2956.

4 Y. Nakagawa, R. Sekiguchi, J. Kawakami and S. Ito, *Org. Biomol. Chem.*, 2019, **17**, 6843–6853.

5 S. O. Aderibigbe and D. M. Coltart, *J. Org. Chem.*, 2019, **84**, 9770–9777.

6 E. Turin, R. M. Nielson and A. E. Merbach, *Inorganica Chim. Acta*, 1987, **134**, 67–78.

7 W. Sheng, Y.-Q. Zheng, Q. Wu, K. Chen, M. Li, L. Jiao, E. Hao, J.-Y. Wang and J. Pei, *Sci. China Chem.*, 2020, **63**, 1240–1245.

8 A. M. M. Schreurs, X. Xian and L. M. J. Kroon-Batenburg, *J. Appl. Cryst.*, 2010, **43**, 70–82.

9 L. Krause, R. Herbst-Irmer, G. M. Sheldrick and D. Stalke, *J. Appl. Cryst.*, 2015, **48**, 3–10.

10 G. M. Sheldrick, *Acta Cryst. A*, 2015, **71**, 3–8.

11 G. M. Sheldrick, *Acta Cryst. C*, 2015, **71**, 3–8.

12 A. L. Spek, *Acta Cryst. D*, 2009, **65**, 148–155.

13 A. L. Spek, *Acta Cryst. C*, 2015, **71**, 9–18.

14 D. Holmes, S. Kumaraswamy, A. J. Matzger and K. P. C. Vollhardt, *Chem. Eur. J.*, 1999, **5**, 3399–3412.
